# Many dynein teams collectively generate high forces during the transport of large organelles

**DOI:** 10.1016/j.bpj.2025.09.012

**Published:** 2025-09-10

**Authors:** Simon Wieland, Christina Steininger, David E. Gitschier, Marius M. Kaiser, Wolfgang Gross, Abdullah R. Chaudhary, Jana Ritschar, Christian Laforsch, Adam G. Hendricks, Holger Kress

**Affiliations:** 1 Biological Physics, University of Bayreuth, Bayreuth, Germany; 2 Animal Ecology I, University of Bayreuth, Bayreuth, Germany; 3 Department of Bioengineering, McGill University, Montreal, Quebec, Canada; 4 Laboratory of Molecular Parasitology, University of Bayreuth, Bayreuth, Germany

## Abstract

The transport of organelles is important to maintain cellular organization and function. Efficient retrograde transport of large organelles with a size of several micrometers requires high collective forces from multiple dynein motors. However, the exact transport forces and their dependence on the cargo size are unknown for large organelles. Furthermore, it is not known how many dynein motors are active during this transport and how they generate high collective forces sufficient to overcome the cytoplasmic drag. We measured forces generated during retrograde transport of phagosomes with diameters between 1 and 5 *μ*m. Forces increased with phagosome volume and ranged from under 10 pN for the smallest up to 160 pN for the largest phagosomes. These forces matched the cytoplasmic drag to achieve equally fast transport with a velocity of 25 ± 4 nm s^−1^ for phagosomes of all sizes. To confirm the need for many dynein motors to generate such high forces, we labeled and quantified dynein on isolated phagosomes. We found up to 250 dyneins on the largest phagosomes and a dynein surface density that was independent of the phagosome size. We connected the dynein numbers and transport forces with a theoretical model of the microtubule distribution around the organelles. The model implies that, because larger organelles displace and bend the microtubules, disproportionately large numbers of dyneins can be active and contribute to the high transport forces of large phagosomes. Our results indicate that, during the transport of large organelles, many dyneins interact with multiple microtubules in a cargo-size-dependent manner to achieve sufficiently large transport forces.

## Significance

Intracellular organelle transport is crucial for cell organization and function. Fast movement of large organelles from the cell periphery to the cell center requires many dynein motors. However, the exact transport forces and motor numbers for organelles with a diameter of several micrometers were unknown. We measured these parameters for organelles between 1 and 5 *μ*m. Our findings show that transport forces surprisingly increase proportionally with organelle volume, with up to 250 motors on the largest organelles. We explained this scaling of forces by the distribution of microtubules around the cargoes. Microtubules, which act as tracks for the motors, bend around the organelles, enabling a disproportionally large number of motors to assist in the transport of very large organelles.

## Introduction

Dynein-driven retrograde transport of organelles plays a key role in many cellular functions (1), such as the transport of endosomes and phagosomes (2,3), axonal transport (4), the nucleus (5), and the spatial organization of cells (6,7). Consequently, mutations in the dynein complex have been linked to a number of developmental, neuro-degenerative, and motor-neuron diseases (8,9). Due to its prominent role in retrograde organelle transport, many studies focused on investigating the mechanochemistry and biochemical regulation of dynein motility (1,10). In vitro force measurements on single mammalian dynein complexes indicate that dyneins produce forces between 1.1 pN (11,12) and 3–4 pN (13,14).

A reason for this large variability of measured forces is that purified cytoplasmic dynein is in an autoinhibited state that produces low forces and is incapable of processive runs (15,16). This weak processivity of single dynein complexes (17,18,19) can additionally lead to an underestimation of their force production in optical-tweezer measurements, because the stall force is not reached before the motors detach (20). However, when bound to cargo adaptors such as dynactin or BICD2, dynein switches from an autoinhibited state to a state that is much more processive and capable of producing higher forces (13,14).

Dynein’s processivity drastically increases in vitro when two or more dynein complexes are active, indicating that individual motors can work together to drive persistent transport (15,18,19,21,22). Moreover, the forces generated collectively by multiple dyneins are additive, enabling robust transport of cargoes in vitro (19,23). This ability to work together is likely caused by adaptor proteins that recruit multiple dyneins (22) and dynein’s large and flexible structure, allowing dynein to adapt its stepping in a load-dependent manner (23). For example, under a resisting load, reduced step sizes and increased backstepping of dynein have been observed, which enable dynein to function as a “gear mechanism” (12,23). Furthermore, a low unbinding rate from microtubules allows dynein to stay attached to microtubules even under strong hindering forces (24,25,26). Together, these molecular adaptations allow multiple dyneins to coordinate their movement and combine their forces (11,19,21).

In living cells, dynein likewise promotes robust and processive retrograde transport of organelles (11,27,28). In addition to the molecular adaptations enabling multiple dyneins to coordinate their stepping (11), dynein clusters into teams on the organelle’s surface (28,29). One of these dynein teams then interacts with a single microtubule so that all dynein molecules in this team contribute to the force production (28). For example, multiple dyneins on intraflagellar transport trains can jointly exert forces of more than 60 pN (30,31). Furthermore, cytoplasmic dynein in living cells can produce retrograde transport forces of about 2–20 pN, also indicating the activity of multiple dynein complexes (11,32,33,34,35).

All in vivo studies focused on small organelles with a size of 1 *μ*m or smaller (for example, latex bead compartments (11,32,33,34) and lipid droplets (35)). However, many organelles that are transported by dynein are much larger, such as nuclei (5,36) or some mitochondria (37). Especially in phagocytic cells, which clear pathogens by phagocytosis, robust transport of the phagosomes is crucial for their degradation (38,39,40). The size of the phagosomes is determined by the size of the internalized object. Often, the phagosomes contain bacteria (41), aggregates of bacteria (42), and particulate contaminants such as microplastic particles (43). Therefore, phagosomes span a large size range of 0.5 *μ*m (small bacteria, atmospheric particulate pollution) to more than 20 *μ*m (bacterial aggregates, microplastic particles) (39,42,44,45).

For organelles with a diameter of several micrometers, cytoplasmic viscosities in the order of 10–100 Pa s have been reported (46,47). Consequently, drag forces on the order of several 100 pN can oppose the intracellular transport of larger organelles (38,48). These drag forces slow down average transport velocities of *μ*m-sized cargoes to 20–60 nm s^−1^ (38,40) compared to the 500–1000 nm s^−1^ that was measured for dynein in vitro (14,23,49). Due to these strong drag forces, the force generated by one team of dyneins moving on a single microtubule are insufficient to overcome the viscous drag of the cytoplasm for larger organelles. Nevertheless, there is fast transport of organelles of different sizes in living cells. For example, the retrograde transport of larger 3-*μ*m organelles is even more persistent than the retrograde transport of smaller 1-*μ*m organelles, which show a very bidirectional motion (38). Possibly, the numbers of involved motors and the resulting transport forces are responsible for this size-dependent transport behavior. However, up to now, neither the magnitude of the transport forces for large cargoes nor their dependence on the cargo size are known. Furthermore, it is currently not known how dyneins work together to generate forces high enough to drive the transport of such large organelles.

We investigated dynein’s ability to collectively drive the transport of large organelles. Using magnetic tweezers, we measured transport forces during the retrograde motion of phagosomes with a diameter of 1–5 *μ*m in living J774A.1 macrophages. Furthermore, we analyzed the motion of the phagosomes to determine the viscous drag opposing retrograde transport of these organelles. To connect the observed transport forces to the underlying molecular machinery, we labeled dynein on isolated phagosomes and quantified the motor numbers using stepwise photobleaching. With a theoretical model taking the displacement of microtubules by the phagosomes into account, we linked the observed transport forces with the corresponding dynein numbers for the phagosomes of different sizes. Our results suggest that, on large organelles, hundreds of dyneins cluster into many teams that likely interact with multiple microtubules. The high forces generated by these dynein teams are required to overcome the drag of the cytoplasm, enabling robust and fast retrograde transport of the organelles.

## Materials and methods

### Cell culture

J774A.1 cells (DSMZ, Braunschweig, Germany) were cultured in 25-cm^2^ flasks (BioLite Cell Culture Treated Flasks, Thermo Fisher Scientific, Waltham, MA) at 37°C, 5% CO_2_, humidified, in DMEM (Gibco life technologies, Carlsbad, CA) with 10% (v/v) fetal bovine serum (Gibco life technologies, Carlsbad, CA) and 1% penicillin/streptomycin (Thermo Fisher Scientific, Waltham, MA). Cells were passaged three times per week. After 35 passages, the cells were replaced.

### Bead opsonization

Carboxylated magnetic microbeads (Dynabeads MyOne, D = 1 *μ*m, catalog no. 65011, Thermo Fisher Scientific, Waltham, MA; Dynabeads M-270, D = 2.8 *μ*m, catalog no. 14305D, Thermo Fisher Scientific, Waltham, MA; micromer-M, D = 2, 4, and 5 *μ*m, catalog nos. 08-02-203, 08-02-403, and 08-02-503, micromod, Rostock, Germany) were opsonized with mouse serum immunoglobulin (Ig)G (EMD Millipore, Darmstadt, Germany) following the procedure in (38,50). Opsonization was verified by immunofluorescence (DyLight-488 goat anti-mouse secondary antibody, Thermo Fisher Scientific, Waltham, MA).

### Magnetic tweezers

The setup, previously described in (51), consists of an inverted microscope (Nikon Eclipse Ti-U, Nikon, Japan) placed on an optical table (PFA51504, Thorlabs, Newton, NJ) with a 60× oil immersion objective (CFI Plan Apochromat *λ* 60× oil objective, NA 1.40, Nikon) and a 20× objective (CFI Plan Achromat 20× objective, NA 0.4, Nikon). Images were acquired using a CMOS camera (Orca Flash V4.0 v2, Hamamatsu, Shizuoka, Japan) under bright-field illumination. The magnetic tweezers consist of a solenoid with a high-permeability soft-iron core that is sharpened to a tip to generate a magnetic gradient field. The coil consists of 1420 loops of a 0.5-mm-thick copper wire looped around a macrolon cylinder with a length of 50 mm and a radius of 10 mm. The *μ*-metal core (Vacuumschmelze Hanau, Germany) has a length of approximately 14.5 cm and a diameter of 5.5 mm. The tip was sharpened to an angle of 40°. The magnetic tweezers were operated at a coil current of 0.1 A (PSI18032-10T, Elektroautomatik, Germany). To this setup, high-precision linear stages (Physik Instrumente, Karlsruhe, Germany: L511 20DG10, *x* and *y* position; M403 10DG, *z* position) for positioning of the tip were added, allowing automated movement and tracking of the tip position during measurements. The force exerted on a magnetic bead was regulated by adjusting the distance between tip and bead. Samples were kept at 37°C during measurements using a custom-built heating system. Evaporating water was replaced at a rate of 180 *μ*L h^−1^ by a syringe pump (LA 30, Landgraf Laborsysteme, Germany). The magnetic force on the beads $F_{\text{MT}}$ depended on the tip-bead distance d and was calibrated with beads suspended in a glycerol-water mixture with known viscosity η. When moving toward the tweezers’ tip, their velocity v(d) was used to determine the magnetic force with Stokes’ law of friction $F_{\text{MT}}\left(d\right)=6\pi \;\eta \;R\;v\left(d\right)$. The calibration procedure is described in detail in the supporting material appendix.

### Transport-force measurements

**Figure 1:**
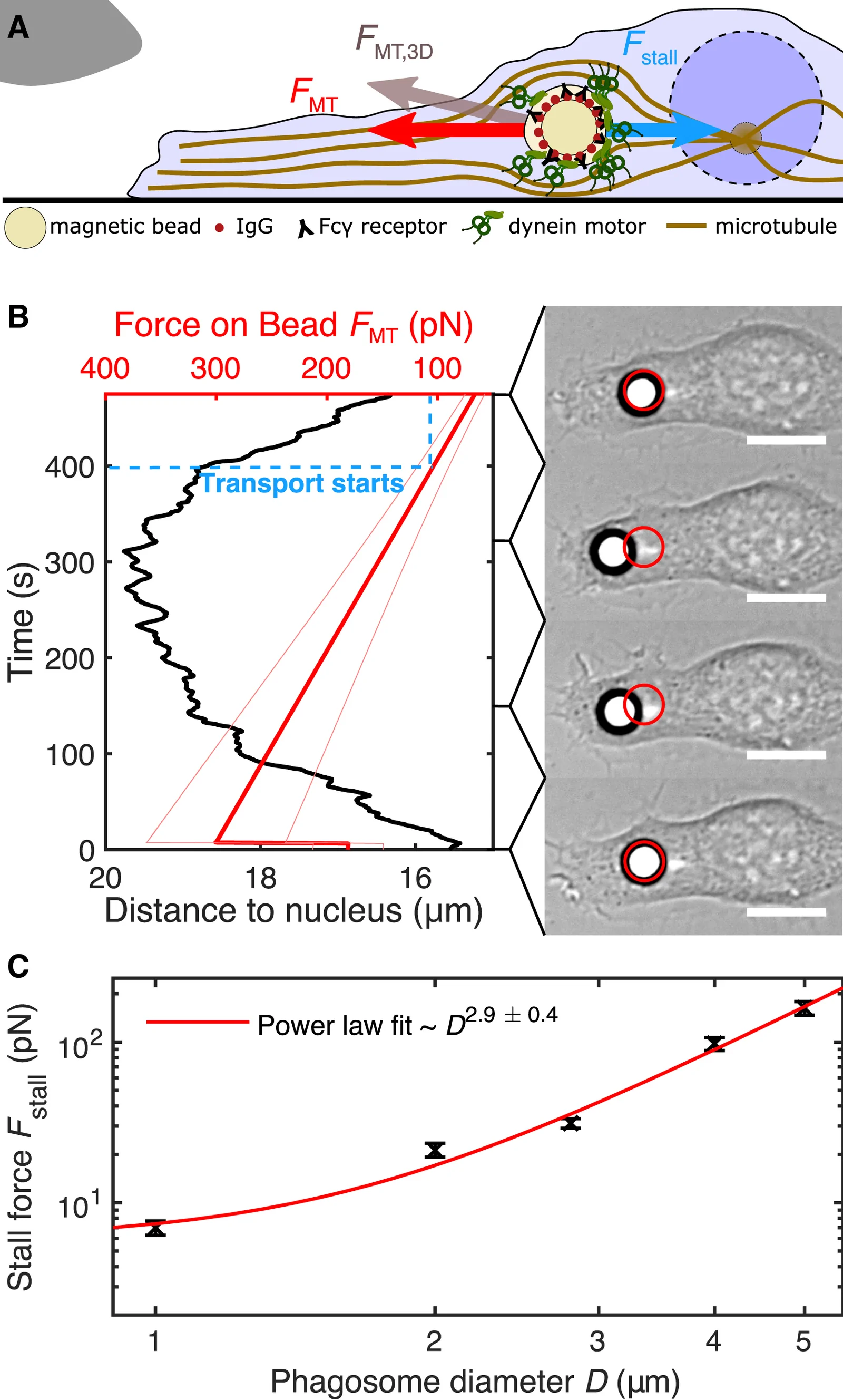
Stall-force measurements using magnetic tweezers. (*A*) In the magnetic gradient field generated by the magnetic tweezers, a force $F_{\text{MT},3D}$ acted on these phagosomes. The projection onto the x-y plane of this magnetic force $F_{\text{MT}}$ was directed against the force $F_{\text{stall}}$ exerted by the molecular motors during retrograde transport of the phagosomes. At $F_{\text{MT}}\approx {-F}_{\text{stall}}$, the angle between $F_{\text{MT},3D}$ and $F_{\text{MT}}$ was approximately 10°–15°. (*B*) Here, an example of a stall-force measurement is shown. At the beginning of each measurement, the phagosomes were pulled into the cell periphery with a high magnetic force $F_{\text{MT}}$. Then, we lowered the force on the phagosome continuously until the force balance $F_{\text{MT}}\approx {-F}_{\text{stall}}$ was crossed and the retrograde transport of the phagosome started. By identifying the onset of transport, we determined the stall force of the phagosome $F_{\text{stall}}$. The red circle in the bright-field images of the measurement marks the original position of the phagosome. Scale bar: 10 *μ*m. (*C*) The measured stall forces strongly depended on phagosome size. We measured average stall forces between 7.0 pN (95% confidence interval: 6.3–7.7 pN) (1-*μ*m phagosomes) and 160 pN (95% confidence interval: 150–180 pN) (5-*μ*m phagosomes). A power-law fit revealed a scaling exponent of 2.9 ± 0.4 of the stall forces $F_{\text{stall}}$ with the phagosome diameter D. The fit included a constant offset to acknowledge that at least one force-producing unit was active during stall-force measurements due to our measurement method. Sample size n= 116 (1-*μ*m phagosomes), 73 (2 *μ*m), 67 (2.8 *μ*m), 46 (4 *μ*m), and 44 (5 *μ*m). Error bars represent the 95% confidence interval estimated by bootstrapping.

The day before the experiments, 40,000 cells were seeded onto a 22 × 40 mm^2^ coverslip (VWR, Radnor, PA). Before the experiments, opsonized beads were diluted 1:1000 in image medium (MEM, 5% HEPES, 1% penicillin/streptomycin) and washed to remove detached IgG. 100 *μ*L of this solution was added to a sample and incubated for 25 min to enable phagocytosis of the beads. The sample was mounted to the setup, and a cell with a suitable phagosome was chosen. The tip of the magnetic tweezers was positioned 25 *μ*m above the phagosome. In this way, the magnetic force was directed parallel to the microtubules and therefore pulled in the opposite direction to the molecular motors (Fig. S1). The magnetic force $F_{\text{MT}}$ was the projection of the three-dimensional magnetic force $F_{\text{MT},3D}$ into the x-y plane (Fig. 1 *A*). The measurements were taken at 3–6 fps, and the phagosome position was tracked in real time using a cross-correlation tracker as previously described (38,52,53). If tracking failed due to changes in the focal plane, radial symmetry detection was used to resume tracking (54). Together with the tracking of the tip position, this enabled a live measurement of the tip-bead distance and therefore of the force acting on the phagosome. With a feedback loop, the tip position was automatically set for each frame of the measurement so that the force on the particle decreased linearly in time. A measurement started with a magnetic force $F_{\text{MT}}$ higher than the expected stall force $F_{\text{stall}}$, pulling the phagosome into the cell periphery. During this phase of the measurement, we ensured that the magnetic forces were not higher than necessary to avoid potential damage to the microtubule cytoskeleton. Usually, forces were chosen such that the velocity of anterograde phagosome movement was not substantially higher than that of the natural retrograde motion mediated by dynein.

After phagosomes were pulled into the cell periphery, the force was decreased constantly over a period of about 5 min, until the maximum tip-bead distance of 500 *μ*m was reached. This relatively long time between reaching the maximal $F_{\text{MT}}$ and the onset of transport ensured that, even if damage to microtubules occurred, they had time to either self-repair (55,56,57) or be replaced by new microtubules (58,59). We defined transport as the movement of a phagosome toward the nucleus that persisted for at least 1 *μ*m. Measurements in which disturbances such as cell migration or drastic changes in cell morphology occurred were excluded, as illustrated in Fig. S2. The time point at which transport toward the nucleus started corresponds to the force balance $F_{\text{stall}}={-F}_{\text{MT}}$, enabling us to determine the stall force $F_{\text{stall}}$. Although during retrograde transport of phagosomes some fluctuations of phagosomes in the y direction occurred, these were always small compared to the transport along the x direction (compare, e.g., Video S3). Furthermore, no considerable motion along the z axis, which would lead to defocusing of the phagosomes, occurred.

Because we quantified retrograde stall forces by observing retrograde transport of phagosomes, we could not measure stall forces of zero, because at least one force-producing unit had to be actively driving retrograde transport. In particular, we did not include stall-force measurements from phagosomes without retrograde transport, since the absence of movement could have multiple causes beyond a stall force. Most importantly, phagosomes that showed no retrograde transport after moving them toward the cell periphery were potentially not completely internalized. Because stall-force assays were done in untransfected, unlabeled cells, we could not directly confirm internalization (e.g., via F-actin dynamics during phagocytosis) and instead relied on active transport as an indicator.

### Cytoplasmic drag

Unloaded phagosome velocities have been shown to be much lower than the velocity of individual dyneins in in vitro assays (38,40), suggesting that motor forces are balanced by viscous drag forces of the cytoplasm. We aimed to quantify the cytoplasmic drag by analyzing the phagosomes’ movement in cells. The average transport velocity $v_{\text{avg}}$ was determined by fitting a line to the organelle trajectories in time intervals of 30 s, yielding $v_{\text{avg}}$ as a function of the average magnetic force $F_{\text{MT},\text{avg}}$ exerted on the organelle during that time interval (Fig. S3). Assuming Stokes’ law of friction, the average velocity depends linearly on the average force $F_{\text{MT},\text{avg}}$:

(1) $$v_{\text{avg}}\left(F_{\text{MT},\text{avg}}\right)=\frac{1}{3\pi \;\eta_{\text{eff}}\;D}\left(F_{\text{stall}}-F_{\text{MT},\text{avg}}\right)\text{.}$$

This equation was fitted to obtain the effective cytoplasmic drag $\eta_{\text{eff}}$ and to verify the analysis of the average stall force $F_{\text{stall}}$. The uncertainty of the fitted parameters was estimated by bootstrapping. For each phagosome size, random subsets of 100 data points were sampled from all data points and fitted with Eq. 1. This was repeated 1000× each, which was sufficient for the mean of all fit results to converge. The standard deviation of the fitted parameters corresponded to their uncertainty.

### siRNA knockdown of dynein

We reduced dynein expression with a small interfering RNA (siRNA) knockdown of the dynein intermediate chain *dync1i2*. A day before treatment, 80,000 cells were seeded on 18-mm coverslips in a 12-well plate. Per well, 3 *μ*L of the transfection reagent (Lipofectamine RNAiMAX, Thermo Fisher Scientific, Waltham, MA) was diluted in 50 *μ*L of Opti-MEM medium (Thermo Fisher Scientific, Waltham, MA). Then, per well, 1 *μ*L of 10 *μ*M siRNA stock solution (*dync1i2* siRNA: Silencer Select pre-designed siRNA, siRNA ID s65062, sequence (sense strand): 5′-GCAUCGAGUUGUUAGUUGUtt-3’. Negative-control siRNA: Silencer Select Negative Control #2 siRNA (sequence undisclosed). Thermo Fisher Scientific, Waltham, MA) was added to 50 *μ*L of Opti-MEM, combined with the diluted transfection reagent and incubated for 5 min at room temperature (RT). Finally, 100 *μ*L of this transfection solution (10 pmol siRNA) was added to each well and incubated for 48 h.

Dynein expression was quantified by western blotting following standard western blot protocol (given in the supporting material appendix) and using a mouse anti-dynein intermediate-chain primary antibody (Invitrogen Dynein Monoclonal Antibody (74.1) MA1-070, Thermo Fisher Scientific, Waltham, MA) and mouse anti *α*-tubulin antibody as loading control (alpha-tubulin (DM1A) mouse monoclonal antibody, Cell Signaling Technology, Cambridge, UK). The secondary antibody was horseradish peroxidase-conjugated goat anti-mouse (Immun-Star Goat Anti-Mouse (GAM)-HRP Conjugate #1705047, Bio-Rad Laboratories, Hercules, CA).

### Ciliobrevin D treatment of cells

Complementary to the siRNA knockdown, we measured transport forces in cells treated with ciliobrevin D (EMD Millipore, Merck, Darmstadt, Germany), a small-molecule inhibitor of the ATPase activity of dynein. 20 min before an experiment, the medium in the samples (prepared analogously to the wild-type samples) was exchanged for image medium containing 50 *μ*M ciliobrevin D. At this concentration, the ATPase activity of dynein is reduced to about 50% (60).

### Phagosome isolation

For the isolation of phagosomes, we followed the procedure described in (34,61). Cells were seeded in four culture flasks and grown to 75% confluency. Before the procedure, 500 *μ*L of the respective carboxylate-bead stock solution (micromer-M, D = 2 *μ*m, 3 *μ*m, 4 *μ*m, 5 *μ*m, micromod, Rostock, Germany; 1- and 2.8-*μ*m Dynabeads were excluded due to strong autofluorescence) was washed 3× in PBS and dispersed in 11.5 mL of cell culture medium. The old medium was replaced with 3 mL of the bead-medium solution per flask. The cells were incubated for 60 min at 37°C and 5% CO_2_ to allow phagocytosis. After incubation, the cells were washed with PBS to remove unphagocytosed particles. Cells were scraped from the flasks and centrifuged at 200 × *g* for 2 min. The supernatant was discarded, and the cells were suspended in 1 mL of HB (homogenization buffer, consisting of motility assay buffer [MAB; 10 mM PIPES, 50 mM K-Acetate, 4 mM MgCl_2_, and 1 mM EGTA; pH 7.0] with 10% sucrose, 1× cOmplete EDTA-free protease inhibitors [Sigma Aldrich, Darmstadt, Germany], 10 µM ATP, and 30 µM DTT) . The cells were homogenized by pressing them 40–50 times through a syringe with a 27G needle monitored under a bright-field microscope. After sufficient homogenization, the phagosomes were separated using a strong magnet and washed 3× in HB with all steps performed on ice to maintain protein integrity. Finally, the isolated phagosomes were suspended in 600 *μ*L of homogenization buffer, aliquoted into 30 *μ*L, and stored at −80°C until needed.

### Immunofluorescence of dynein

Before the immunofluorescence experiments, coverslips were treated with a hydrophobic coating. Coverslips were cleaned for 10 min in acetone, sonicated for 20 min in methanol, sonicated for another 20 min in 0.5 M potassium hydroxide (KOH), and rinsed 3× with MilliQ water before drying overnight. Dried coverslips were soaked in Rain X 26014 rain repellent for 5 min, dried for 15 min, and gently rubbed with a Kimwipe. The hydrophobicity was checked by placing a small droplet of MilliQ water on a treated coverslip, showing contact angles of 90°–100°, indicating a successful hydrophobic treatment. Then, small flow channels were assembled from the coverslips and the phagosomes flowed into the flow channels, fixed with 4% paraformaldehyde, and labeled with the primary (Invitrogen Dynein Monoclonal Antibody (74.1) MA1-070, Thermo Fisher Scientific, Waltham, MA) and secondary antibody (Invitrogen Goat Anti-Mouse IgG, DyLight 488 conjugated, Thermo Fisher Scientific, Waltham, MA) following standard protocol (supporting material appendix).

Imaging was performed using a Epi/ total internal reflection fluorescence (TIRF) microscope (Leica Microsystems, Wetzlar, Germany) with an HC PL Apo 100×/1.47 NA oil immersion objective (Leica) and a Leica DFC 9000 GT sCMOS (scientific complementary metal-oxide semiconductor) camera. The samples were illuminated using a 488-nm laser at 4.4-mW power and imaged with an exposure time of 500 ms and 2 × 2 binning.

We validated our immunofluorescence assay by completely replicating our experiments with another batch of isolated phagosomes, different types of primary and secondary antibodies, a different fluorophore, and a different microscopy setup (Fig. S4). In addition, we labeled kinesin-2. An overview of the replication experiment is given in the supporting material appendix.

### Dynein quantification

The phagosomes were imaged in the epifluorescence mode of the microscope. Since, occasionally, individual phagosomes were not completely isolated from cell debris, they were very bright and overexposed. These phagosomes were not considered in the evaluation. The integrated dynein intensity $I_{\text{dyn}}$ was measured in a circular region of interest with a diameter of 1.5×D. For each phagosome, the background intensity was measured in a similarly sized region of interest nearby and subtracted from the dynein signal. Unspecific binding of the primary antibody to the carboxylate beads was corrected by subtracting the fluorescence of the stained unphagocytosed beads from the dynein fluorescence. The values for $I_{\text{dyn}}$ are provided in Table S4.

For each phagosome sample, photobleaching experiments were performed by imaging the field of view for 10 min with a rate of 2 fps. We detected steps in the bleaching curves using an algorithm established by Chen et al. (62), based on a two-sample *t*-test to identify time intervals with constant mean and variance, fitting a piecewise constant function to the photobleaching curves (Figs. S5 *A*, *B*, and S6). The resulting step-size distributions (Fig. S5, *C* and *D*) were fitted using a Gaussian mixture model (34,62), yielding a unitary step size $I_{\text{unitary}}^{\text{epi}}=$ (160 ± 30) arbitrary units and $I_{\text{unitary}}^{\text{TIRF}}=$ (235 ± 50) arbitrary units. Details of the Gaussian mixture model fit are given in the supporting material appendix. We validated the photobleaching procedure by determining the degree of labeling of the secondary antibodies by stepwise photobleaching and comparing this value to the degree of labeling measured spectroscopically (supporting material appendix).

We then quantified the number of fluorophores bound to a primary-secondary antibody complex. We diluted the primary antibodies 1:10,000 in 60 *μ*L of MAB, flowed them into the sample chamber, and let them attach to the coverslip overnight at 4°C. Then, we blocked the coverslip surface with Pluronic F-127. We incubated the sample for 45 min at RT with the secondary antibodies, in the same way that we treated the phagosome samples (Fig. S5 *G*).

The samples were imaged in the TIRF mode of the microscope. We measured the fluorescence intensity of each individual spot on the coverslip and corrected it for background. Then, we calculated the number of fluorophores in a single primary-secondary antibody complex:

(2) $$N_{\text{pAB}}=\frac{I_{\text{pAB}}}{I_{\text{unitary}}^{\text{TIRF}}}\text{.}$$

On average, we found that 70 ± 40 fluorophores were bound to a single primary antibody (Fig. S5 *H*). The number is higher than in previous studies because multiple secondary antibodies bind to one primary antibody.

With the number of fluorophores per primary/secondary antibody complex $N_{\text{pAB}}$, the total intensity of a single dynein-antibody complex is $I_{\text{singledyn}}=2\times N_{\text{pAB}}\times I_{\text{unitary}}^{\text{epi}}$, since dynein has two copies of the intermediate chain and thus two binding sites for the monoclonal primary antibody. Consequently, the number of dyneins on a phagosome is $N_{\text{dyn}}=I_{\text{dyn}}/I_{\text{singledyn}}$. Examples of photobleaching curves, phagosomes, their corresponding intensities, and motor numbers are shown in Fig. S6.

Furthermore, we prepared samples in which we stained the membrane of the isolated phagosomes with FM 4-64 (Thermo Fisher Scientific, Waltham, MA). The intensity of the phagosomal membrane $I_{\text{mem}}$ was evaluated analogously to the dynein intensity $I_{\text{dyn}}$. We evaluated the scaling behavior of the motor-area density by evaluating $I_{\text{dyn}}/I_{\text{mem}}$ for all phagosome diameters D.

### Confocal images of the microtubule skeleton

100,000 cells were seeded on an 18-mm round coverslip (VWR, Radnor, PA) 1 day before the sample preparation. The beads (*D* = 2, 4, and 5 *μ*m) were washed 3× in PBS, diluted 1:1000 in cell culture medium, and 100 *μ*L of the diluted bead solution was added to the cells and incubated for 60 min to allow phagocytosis. Then, cells were washed 3× with PBS, fixed with 4% paraformaldehyde in PBS for 15 min at RT, and labeled with the primary antibody (*α*-tubulin (DM1A) mouse monoclonal antibody, Cell Signaling Technology, Cambridge, UK) and secondary antibody (Invitrogen goat anti-mouse IgG, DyLight 488 conjugated, Thermo Fisher Scientific, Waltham, MA) according to standard protocol (supporting material appendix). Imaging was performed using a custom-built spinning-disk confocal microscope (63) consisting of a Leica DMI 6000 body (Leica Microsystems, Wetzlar, Germany) equipped with a CSU-X1 spinning-disk unit (Yokogawa Microsystems, Tokyo, Japan), a HC PL APO 100×/1.4 NA oil immersion objective (Leica Microsystems), and a Photometrics Evolve 512 charge-coupled device camera (Photometrics, Tucson, AZ). The images show the displacement and bending of the microtubule cytoskeleton caused by the phagosomes without an external magnetic force.

### Modeling of elastic forces due to deformations of the cell cortex

We quantified possible forces due to elastic deformations (Fig. S7 *A*) of the cell cortex by the phagosomes by modeling the deformations heuristically as cosine shaped with smooth transitions to the undeformed cell at its edges and a radius of curvature that is equal to the bead radius at its maximum (Fig. S7, *B* and *C*). For a bead with radius R=D/2 that is at position $x_{B}$ along the x axis, the deformation of the cell cortex along the x’ axis can be written as

(3) $$f\left(x^{\prime }\right)=h\left[\frac{1}{2}\cos\left(\frac{1}{a}\left(x^{\prime }-x_{B}^{\prime }\right)\right)+\frac{1}{2}\right]\text{,}$$

where h is the height of the deformation perpendicular to the undeformed cortex, and πa is the radius of the deformed region of the cell cortex. To determine the width a of the deformation, we used the condition that the radius of curvature of the deformation at $x’=x{’}_{B}$ must be equal to the bead radius R.

Assuming that the deformation of the cell cortex is rotationally symmetrical around $x{’}_{B}$, we calculated the area of the deformed cell cortex. With the known tension of the cell cortex of J774A.1 cells σ= 140 ± 20 pN *μ*m^−1^ (64), we determined the energy $W_{\text{deformation}}$ required for a given deformation. The force that pushes the bead along the x axis is the spatial derivative of this energy, which was evaluated numerically:

(4) $$F_{\text{Actin}}=-\frac{\partial }{\partial x_{B}}W_{\text{deformation}}\text{.}$$

A step-by-step derivation of the model is given in the supporting material appendix.

### Creep-recovery measurements

To analyze creep of the cell cortex under sustained stress, samples were prepared in the same way as for the transport-force measurements. The 2.8-*μ*m beads opsonized with IgG were added to the cells, incubated for 25 min to enable phagocytosis, the sample was mounted to the magnetic tweezers setup and a suitable cell was chosen. Then a measurement was started and images were acquired with a frame rate of 3–6 fps. During the measurements, real-time tracking was used to follow the phagosome’s position. Using the feedback loop of the magnetic tweezers, the MT tip was positioned automatically to keep the force on the phagosomes constantly at 100 pN. The current of the magnetic tweezers was turned on and off periodically with a period of 60 s to measure the step response of the system (magnetic force on, creep; magnetic force off, recovery).

## Results

### Transport forces increase with the third power of the organelle diameter

Murine macrophages were incubated with IgG-opsonized magnetic beads to generate phagosomes with a defined diameter D. We measured the stall forces $F_{\text{stall}}$ of these phagosomes during their retrograde transport using magnetic tweezers (Figs. 1 *A*, *B*, S2, and S9; Videos S1, S2, S3, S4, and S5). To this end, phagosomes containing magnetic beads were first pulled away from the nucleus toward the cell periphery with a high magnetic force $F_{\text{MT}}$. We positioned the tip of the magnetic tweezers in a line with the cells’ long axes, such that the magnetic forces and phagosome motions were parallel to the orientation of the microtubule cytoskeleton (Fig. S1) and opposite to the direction of motion of dynein (65). Then, the magnetic force was gradually lowered until transport of the phagosome started. At the onset of transport, the force balance between the magnetic force and the stall force generated by the molecular motors $F_{\text{MT}}=-F_{\text{stall}}$ was crossed, which allowed us to quantify $F_{\text{stall}}$ (Figs. 1, *B* and S2). The results of the force measurements for phagosomes with diameters D of 1, 2, 2.8, 4, and 5 *μ*m (Fig. 1 *C*) are summarized in Table 1. We found that the stall forces of the phagosomes strongly depended on their size. For the smallest phagosomes, we measured an average transport force of 7.0 pN (95% confidence interval: 6.3–7.7 pN). The forces continuously increased with phagosome size up to 160 pN (95% confidence interval: 150–180 pN) for the largest phagosomes. To quantify the size dependence of the observed stall forces, we fitted a power law of the form

(5) $$F_{\text{stall}}\left(D\right)=a\times {\left(\frac{D}{1\;\mu m}\right)}^{\alpha }+c\text{.}$$

**Table 1:** Summary of the Measured Stall forces $F_{\text{stall}}$, Effective Cytoplasmic Viscosities $\eta_{\text{eff}}$, and the Number of dyneins per Phagosome for all Phagosome Sizes

D (*μ*m)	$F_{\text{stall}}$ (pN)	$\eta_{\text{eff}}$ (Pa s)	$N_{\text{dyn}}$
1	7.0 (6.3–7.7)	10 ± 7	–
2	21 (19–23)	25 ± 10	30 (10–50)
2.8	31 (29–33)	28 ± 3	60 (0–140)
4	100 (90–110)	100 ± 30	150 (40–260)
5	160 (150–180)	100 ± 20	240 (70–410)

*F*_stall_ and number of dyneins per phagosome are presented as mean and 95% confidence interval estimated by bootstrapping.

Video S1. An example measurement of the stall force $F_{\text{stall}}$ for a phagosome with a diameter of 1 μm At the beginning of the measurement, a high magnetic force $F_{\text{magn}}$ is exerted on the phagosome, leading to an increasing distance between the phagosome and the nucleus. Then, we lower the force on the phagosome linearly with time until the force balance $F_{\text{magn}}\approx {-F}_{\text{stall}}$ (indicated by the dashed blue line) is crossed. Then, retrograde transport of the phagosome starts and the distance to the nucleus decreases. In the case shown here, $F_{\text{stall}}$ is about 4.7 ± 0.5 pN. The magnitude and direction of the magnetic pulling force are highlighted by the red arrow, whereas the blue arrow indicates magnitude and direction of the cellular transport forces.

Video S2. An example measurement of the stall force $F_{\text{stall}}$ for a phagosome with a diameter of 2 μm At the beginning of the measurement, a high magnetic force $F_{\text{magn}}$ is exerted on the phagosome, leading to an increasing distance between the phagosome and the nucleus. Then, we lower the force on the phagosome linearly with time until the force balance $F_{\text{magn}}\approx {-F}_{\text{stall}}$ (indicated by the dashed blue line) is crossed. Then, retrograde transport of the phagosome starts and the distance to the nucleus decreases. In the case shown here, $F_{\text{stall}}$ is about 13 ± 4 pN. The magnitude and direction of the magnetic pulling force are highlighted by the red arrow, whereas the blue arrow indicates magnitude and direction of the cellular transport forces.

Video S3. An example measurement of the stall force $F_{\text{stall}}$ for a phagosome with a diameter of 2.8 μm At the beginning of the measurement, a high magnetic force $F_{\text{magn}}$ is exerted on the phagosome, leading to an increasing distance between the phagosome and the nucleus. Then, we lower the force on the phagosome linearly with time until the force balance $F_{\text{magn}}\approx {-F}_{\text{stall}}$ (indicated by the dashed blue line) is crossed. Then, retrograde transport of the phagosome starts and the distance to the nucleus decreases. In the case shown here, $F_{\text{stall}}$ is about 27 ± 4 pN. The magnitude and direction of the magnetic pulling force are highlighted by the red arrow, whereas the blue arrow indicates magnitude and direction of the cellular transport forces. In this video, some fluctuations of the phagosome perpendicular to its primary transport directions are noticeable. However, these fluctuations were always small compared to the main retrograde motion.

Video S4. An example measurement of the stall force $F_{\text{stall}}$ for a phagosome with a diameter of 4 μm At the beginning of the measurement, a high magnetic force $F_{\text{magn}}$ is exerted on the phagosome, leading to an increasing distance between the phagosome and the nucleus. Then, we lower the force on the phagosome linearly with time until the force balance $F_{\text{magn}}\approx {-F}_{\text{stall}}$ (indicated by the dashed blue line) is crossed. Then, retrograde transport of the phagosome starts and the distance to the nucleus decreases. In the case shown here, $F_{\text{stall}}$ is about 68 ± 15 pN. The magnitude and direction of the magnetic pulling force are highlighted by the red arrow, whereas the blue arrow indicates magnitude and direction of the cellular transport forces.

Video S5. An example measurement of the stall force $F_{\text{stall}}$ for a phagosome with a diameter of 5 μm At the beginning of the measurement, a high magnetic force $F_{\text{magn}}$ is exerted on the phagosome, leading to an increasing distance between the phagosome and the nucleus. Then, we lower the force on the phagosome linearly with time until the force balance $F_{\text{magn}}\approx {-F}_{\text{stall}}$ (indicated by the dashed blue line) is crossed. Then, retrograde transport of the phagosome starts and the distance to the nucleus decreases. In the case shown here, $F_{\text{stall}}$ is about 170 ± 30 pN. The magnitude and direction of the magnetic pulling force are highlighted by the red arrow, whereas the blue arrow indicates magnitude and direction of the cellular transport forces.

We found a scaling exponent α of 2.9 ± 0.4. For the scaling factor a, we observed a value of (1.5 ± 0.5) pN, and for the offset c a value of (6 ± 4) pN. The offset was included to acknowledge that at least one force-producing unit was active during stall-force measurements due to our measurement method.

### High transport forces are necessary to overcome the drag of the cytoplasm

**Figure 2:**
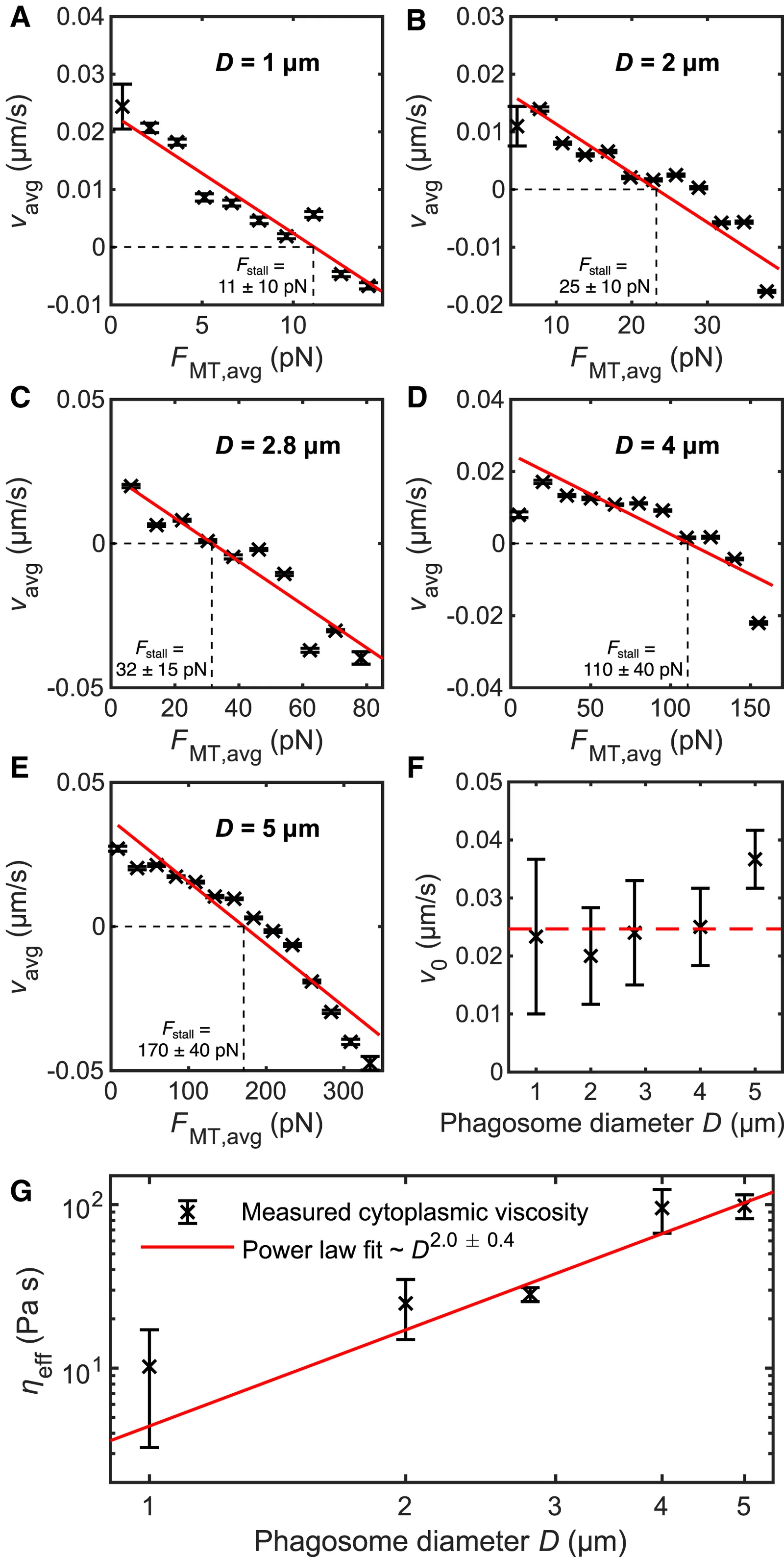
Analysis of the cytoplasmic drag. (*A*–*E*) We determined the effect of the drag of the cytoplasm during intracellular organelle transport by analyzing the average transport velocity $v_{\text{avg}}$ as a function of the average magnetic force $F_{\text{MT},\text{avg}}$. The $v_{\text{avg}}\left(F_{\text{MT},\text{avg}}\right)$ data followed approximately a linear relationship, which we fitted with Stokes’ law of friction (Eq. S1, red lines). From the fit we obtained the average stall forces and the effective cytoplasmic drag. The average stall force $F_{\text{MT},\text{avg}}\left(v_{\text{avg}}=0\right)$ is very consistent with $F_{\text{stall}}$ obtained from the individual analysis of the trajectories (Table 1), supporting the validity of our transport criterion. Sample size n= 840 derived from 116 individual trajectories (1-*μ*m phagosomes), 1613 from 73 (2 *μ*m), 862 from 67 (2.8 *μ*m), 977 from 46 (4 *μ*m), and 764 from 44 (5 *μ*m). For clarity, data points were displayed as average over intervals of 1.5 pN (1-*μ*m phagosomes), 3 pN (2 *μ*m), 8 pN (2.8 *μ*m), 15 pN (4 *μ*m), and 25 pN (5 *μ*m). Error bars represent the SE of mean. (*F*) The free-transport velocity $v_{0}=v_{\text{avg}}\left(F_{\text{MT},\text{avg}}=0\right)$ did not systematically depend on the diameter D. The average $v_{0}$ was (25 ± 4) nm s^−1^. (*G*) The effective cytoplasmic drag $\eta_{\text{eff}}$ strongly depended on D. We observed effective cytoplasmic drag coefficients between (10 ± 7) Pa s and (100 ± 20) Pa s. The scaling exponent of the size-dependence of $\eta_{\text{eff}}$ was 2.0 ± 0.4.

To enable robust and fast intracellular transport of organelles, the forces generated by the molecular motors need to be high enough to overcome the drag of the cytoplasm. We therefore determined the effective cytoplasmic drag coefficient $\eta_{\text{eff}}$ by analyzing the average transport velocity $v_{\text{avg}}$ of the phagosomes in the transport-force measurements as a function of the average magnetic force $F_{\text{MT},\text{avg}}$ over intervals of 30 s (Fig. S3). In these measurements, the phagosomes were first pulled toward the cell periphery with a high magnetic force $F_{\text{MT},\text{avg}}$ (increasing distance to the nucleus, $v_{\text{avg}}<0$). Then, the magnetic force was gradually lowered, and dynein-mediated transport to the nucleus was started (distance to the nucleus decreased, $v_{\text{avg}}>0$). In each 30-s interval, the magnetic force $F_{\text{MT},\text{avg}}$ changed only slightly and was assumed to be constant. Overall, $v_{\text{avg}}\left(F_{\text{MT},\text{avg}}\right)$ approximately followed a linear relationship (Fig. S3 *B*; Fig. 2 *A*–*E*). We fitted these data with Stokes’ law of friction (Eq. 1) to obtain the stall force and to quantify the cytoplasmic drag. Since the cytoplasm is not a simple Newtonian fluid, we had to introduce an effective cytoplasmic drag coefficient $\eta_{\text{eff}}$, which depends on the phagosome diameter D (Table S1).

The average stall forces $F_{\text{MT},\text{avg}}\left(v_{\text{avg}}=0\right)$ extracted from this analysis agree with the stall forces extracted directly from the individual phagosome trajectories. From the cytoplasmic viscosity analysis, we determined stall forces (Table S1) between (11 ± 10) pN for the smallest phagosomes (D = 1 *μ*m) and (170 ± 40) pN for the largest phagosomes (D = 5 *μ*m). These stall forces provide a verification of the first analysis method since they do not require the identification of the onset of transport. Therefore, these results confirm the stall-force values shown in Table 1. Here, the relatively large uncertainty of the stall force observed for the 1-*μ*m phagosomes was likely related to their low transport persistence, which was also reported previously (38). Due to the lower persistence of retrograde motion, individual $v_{\text{avg}}\left(F_{\text{MT},\text{avg}}\right)$ data points had a larger variance for the 1-*μ*m phagosomes compared to larger phagosomes, and therefore fitting Eq. 1 was associated with larger uncertainties.

The free-transport velocity $v_{0}=v_{\text{avg}}\left(F_{\text{MT},\text{avg}}=0\right)$, which is the transport velocity of the organelles without an external load, did not systematically depend on phagosome size (Fig. 2 *F*; Table S1). We measured an average free-transport velocity of (25 ± 4) nm s^−1^. Similar studies on retrograde transport of *μ*m-sized cargoes consistently reported similar average transport velocities of 20–60 nm s^−1^ (38,40).

In contrast, the effective cytoplasmic drag $\eta_{\text{eff}}$ depended strongly on the phagosome size (Fig. 2 *G*; Table 1), which is likely related to the fact that the cytoplasm is a complex fluid (48,66). Although the $v_{\text{avg}}\left(F_{\text{MT},\text{avg}}\right)$ relationship heuristically followed Stokes’ law of friction, the size dependence of the drag coefficient obtained in this way indicated the non-Newtonian nature of the cytoplasm. We assume that this size dependence of the drag coefficient was caused by a combination of viscous forces of the cytoplasm and inelastic deformations of elements, such as actin filaments and microtubules; however, more research would be needed to obtain a mechanistic model of this cytoplasmic drag coefficient $\eta_{\text{eff}}$. Nevertheless, for the smallest phagosomes, we observed an effective cytoplasmic viscosity of (10 ± 7) Pa s, which continuously increased up to (100 ± 20) Pa s for the largest phagosomes. We quantified the size dependence of the cytoplasmic viscosity heuristically using a power law:

(6) $$\eta_{\text{eff}}\left(D\right)=d\times {\left(\frac{D}{1\mu m}\right)}^{\beta }\text{.}$$

For the scaling factor d, we found (4.3 ± 1.3) Pa s. We observed a scaling exponent β of 2.0 ± 0.4, which agrees very well with the scaling behavior of the stall forces. Since the free-transport velocity $v_{0}$ was independent of D, the scaling behavior of the observed stall forces can be written as

(7) $$F_{\text{stall}}\left(D\right)\;\propto \;aD^{\alpha }\;\propto \;dD^{\beta }\times D\;\Rightarrow \;\alpha =\beta +1$$

using Stokes’ law. Therefore, the independently observed scaling exponents α and β are very consistent. Overall, this analysis shows that the very high transport forces measured for the large organelles are necessary to overcome the drag of the cytoplasm.

### Dynein is the main driver of retrograde phagosomal transport

To investigate the underlying molecular mechanisms of the observed transport forces, we examined the role of dynein during the retrograde transport of the phagosomes. We tested the hypothesis that dynein is primarily responsible for driving the observed retrograde organelle transport by reducing the expression level of dynein and by inhibiting its ATPase activity. We reduced dynein intermediate-chain *dync1i2* expression levels by 50% using RNAi (Fig. S10 *A*). The corresponding stall forces measured for phagosomes with a diameter of 5 *μ*m were reduced to about 50% compared to wild-type cells and cells treated with negative-control siRNA. Consistently, the inhibition of dynein’s ATPase activity with ciliobrevin D at a concentration at which the ATPase activity of dynein is reduced to about 50% (60) led to a 50% decrease in the stall force of the phagosomes (Fig. S10 *B*). Therefore, these results indicate that dynein is the main driver of retrograde transport of the phagosomes and that its force production is directly related to the number of active dynein complexes.

Alternatively, the measured forces during retrograde transport could originate from elastic deformations of the cell cortex by the phagosomes (Fig. S7 *A*). To evaluate this, we modeled the cortex deformations by phagosomes of different sizes (supporting material materials and methods; Fig. S7, *B* and *C*). With the known cortical tension of J774A.1 (64), we derived the maximal elastic force that might push the phagosomes toward the nucleus (Fig. S7 *D*). This maximal elastic force would only be reached if the cell had no possibility to adapt to extracellular forces (i.e., if it behaved as a purely elastic material). The elastic forces, dependent on phagosome size and position, ranged from 10 to 400 pN, similar to the measured transport forces. However, the elastic forces increased linearly with phagosome size, not matching the $D^{3}$ scaling of the measured transport forces (Fig. S7, *E* and *F*).

Furthermore, the cell cortex is not purely elastic but has both elastic and viscous properties (67,68,69), as also shown by our creep-recovery measurements (Fig. S11). We observed that, under a sustained magnetic force, the cell cortex is subjected to creep, which leads to dissipation of the cortical tension, so that the phagosome does not fully relax to its original position once the magnetic force is released. Our measurements indicate that this creep occurs on timescales of about 15–20 s, which coincides with the time for actin turnover and remodeling of the cell cortex (67,68). It is well established that, on timescales shorter than the actin turnover time, the cell cortex behaves like an elastic solid, and, on longer timescales, it transitions to a viscous behavior (67). In our measurements of the transport force, the time between reaching the maximal magnetic force and the onset of retrograde transport is much longer than the remodeling time of the cell cortex, ranging from 250 s (2.8-*μ*m phagosomes) to 400 s (1- and 2-*μ*m phagosomes). Therefore, we conclude that most elastic forces of the cell cortex were already dissipated at the onset of transport and only molecular motors contributed to the measured forces.

### The dynein surface density does not depend on the phagosome diameter

**Figure 3:**
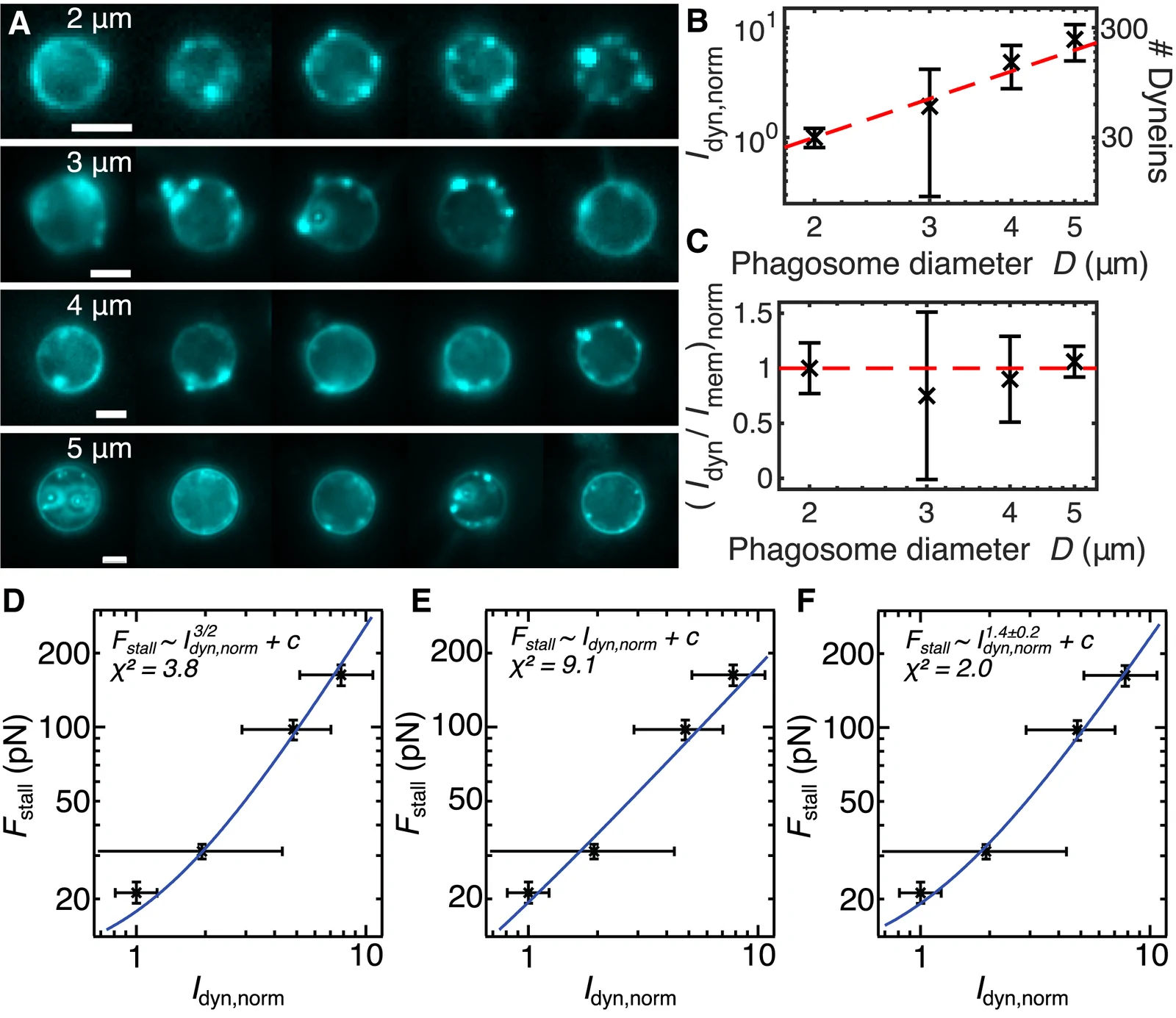
Labeling and quantification of dynein on isolated phagosomes. (*A*) Dynein forms several distinct clusters on phagosomes of all sizes, indicating that multiple teams of dyneins are present on the phagosomes’ surfaces. Scale bars: 2 *μ*m. (*B*) The normalized fluorescence intensity of the labeled dynein $I_{\text{dyn},\text{norm}}$ increased with $D^{2}$, showing that the dynein number is proportional to the phagosome surface. Sample size n= 92 (2-*μ*m phagosomes), 25 (3 *μ*m), 31 (4 *μ*m), and 22 (5 *μ*m). Error bars represent the 95% confidence interval estimated by bootstrapping. (*C*) We confirmed the result by evaluating the fluorescence intensity of dynein relative to the fluorescence of the labeled phagosomal membrane $I_{\text{dyn}}/I_{\text{mem}}$. This ratio did not depend on D, indicating that the dynein surface density was independent of the phagosome size. Sample size n= 182 (2 *μ*m phagosomes), 272 (3 *μ*m), 224 (4 *μ*m), and 327 (5 *μ*m). (*D*–*F*) Force-motor scaling relationships fitted with $F_{\text{stall}}\;∼\;I_{\text{dyn},\text{norm}}^{3/2}+c$ (*D*), $F_{\text{stall}}\;∼\;I_{\text{dyn},\text{norm}}+c$ (*E*), and a free scaling exponent (*F*). The constant offset c was included to acknowledge that, due to our measurement method, at least one force-producing unit was active during stall-force measurements. The $\chi^{2}$ goodness-of-fit values seem to suggest a better fit for $F_{\text{stall}}\;∼\;I_{\text{dyn},\text{norm}}^{3/2}+c$, which is consistent with the scaling exponent of 1.4 ± 0.2 for the free fit.

Next, we aimed to connect the measured transport forces to the number of dyneins present on the phagosomes. To this end, we isolated phagosomes from the cells and immunofluorescently labeled dynein on their surface (Fig. S4). The dynein fluorescence on the phagosomes was enriched at discrete spots, indicating that dynein clustered into multiple dynein teams (Fig. 3 *A*), consistent with previous studies investigating the distribution of dyneins on organelles (28,29). We were able to replicate these experiments with another set of isolated phagosomes and different types of primary and secondary antibodies (Fig. S12). Additionally, we labeled kinesin-2 on the isolated phagosomes, which was reported to be present on isolated phagosomes previously (32,34). Kinesin did not form clusters on phagosomes (Fig. S12 *C*), indicating that the punctate localization is specific to dynein.

We quantified the scaling behavior of the amount of dynein attached to the phagosomes by evaluating their fluorescence intensity. The dynein fluorescence increased approximately with $D^{2}$ (Fig. 3 *B*). Fitting a power law $I=I_{0}D^{\gamma }$ to the dynein fluorescence revealed a scaling exponent γ of 2.30 ± 0.15, which is distinct from the scaling exponent α= 2.9 ± 0.4 observed for the transport forces. We replicated this result with another set of isolated phagosomes and different types of primary and secondary antibodies and fluorophores (Fig. S13). To further exclude possible artifacts (e.g., size-dependent damage of the phagosomes caused by the isolation process), we additionally labeled the phagosomal membrane to quantify the amount of dynein per membrane area. We found that the dynein intensity per membrane intensity did not depend on D, indicating that the dynein surface density of the phagosomes is size independent (Fig. 3 *C*).

In addition to the scaling behavior of the amount of dynein, we also quantified the number $N_{\text{dyn}}$ of individual dynein motors attached to phagosomes in the size range 2–5 *μ*m using stepwise photobleaching (34,62) (Figs. S5 and S6). The stepwise photobleaching provides a calibration of the measured dynein intensity of the phagosomes to determine the absolute number of molecular motors. Therefore, the observed $D^{2}$ scaling behavior of the dynein numbers is independent of this calibration. We estimated 30 dyneins (95% confidence interval: 10–50) were bound to the smallest phagosomes (D = 2 *μ*m). The number of motors continuously increased with increasing D, up to 250 dyneins (95% confidence interval: 70–410) for the largest phagosomes with a diameter of D = 5 *μ*m (Table 1). On average, we measured a dynein surface density $\rho_{\text{Dyn}}$ of 3 ± 1 dyneins *μ*m^−2^. Two earlier studies measured the numbers of dyneins attached to phagosomes isolated from J774 cells and observed similar dynein surface densities. For early phagosomes with a diameter of 0.5 *μ*m, a dynein density of 4 dyneins *μ*m^−2^ was found (65). Similarly, for late phagosomes, dynein densities of 3–6 dyneins *μ*m^−2^ (34) and 6.5 dyneins *μ*m^−2^ (65) were measured. These numbers further reinforce our hypothesis that the dynein surface density is independent of phagosome size over a large range (Fig. S14). The resulting force generated per dynein $F_{\text{dyn}}=F_{\text{stall}}/N_{\text{dyn}}$ was approximately 0.5–0.7 pN, indicating that not all dyneins attached to a phagosome were active during retrograde transport. Similar to this result, Ucar and Lipowsky observed in simulations of collective force generation by up to 7 dyneins that the collective force increased linearly by ca. 0.7 pN per dynein (70).

To test whether the scaling behavior of the dynein numbers on the phagosomes and the transport forces were indeed distinct from each other, we analyzed the stall forces as a function of the dynein fluorescence. If $F_{\text{stall}}∼D^{3}$ and $I_{\text{dyn}}∼D^{2}$, then it should be expected that $F_{\text{Stall}}\;∼\;I_{\text{dyn}}^{3/2}$. Otherwise, if the transport forces scaled purely with the number of dyneins attached to the organelles, as observed for small dynein numbers <10 dyneins, where all dyneins participate in the transport (11,70), then $F_{\text{stall}}∼I_{\text{dyn}}$ should be expected. We fitted $F_{\text{stall}}$ to both $I_{\text{dyn}}^{3/2}$ and $I_{\text{dyn}}$ (Fig. 3, *D* and *E*). Like the power law fit in Eq. 5, these fits included a force offset, because our method required the phagosomes to be actively transported toward the nucleus to measure a stall force. Therefore, we always measured the stall force of at least one force-producing unit. Both the $F_{\text{stall}}\;∼\;I_{\text{dyn}}^{3/2}$ and $F_{\text{stall}}\;∼\;I_{\text{dyn}}$ fit intersect all datapoints within their margin of errors. However, the $\chi^{2}$ goodness-of-fit values were more in favor of $F_{\text{stall}}\;∼\;I_{\text{dyn}}^{3/2}$ ($\chi^{2}$: 3.8) than of $F_{\text{stall}}\;∼\;I_{\text{dyn}}$ ($\chi^{2}$: 9.1). When leaving the exponent as a free fit parameter, the model converges to an exponent of 1.4 ± 0.2 ($\chi^{2}$: 2.0), which is more consistent with $F_{\text{stall}}\;∼\;I_{\text{dyn}}^{3/2}$ (Fig. 3 *F*). The replication dataset further supports a force-motor scaling with the exponent 3/2, with a $\chi^{2}$ value of 4.5 for the $F_{\text{stall}}\;∼\;I_{\text{dyn}}^{3/2}$ fit, compared to a $\chi^{2}$ value of 17.7 for the $F_{\text{stall}}\;∼\;I_{\text{dyn}}$ fit (Fig. S15).

### Large organelles displace microtubules, enabling more dyneins to be active

**Figure 4:**
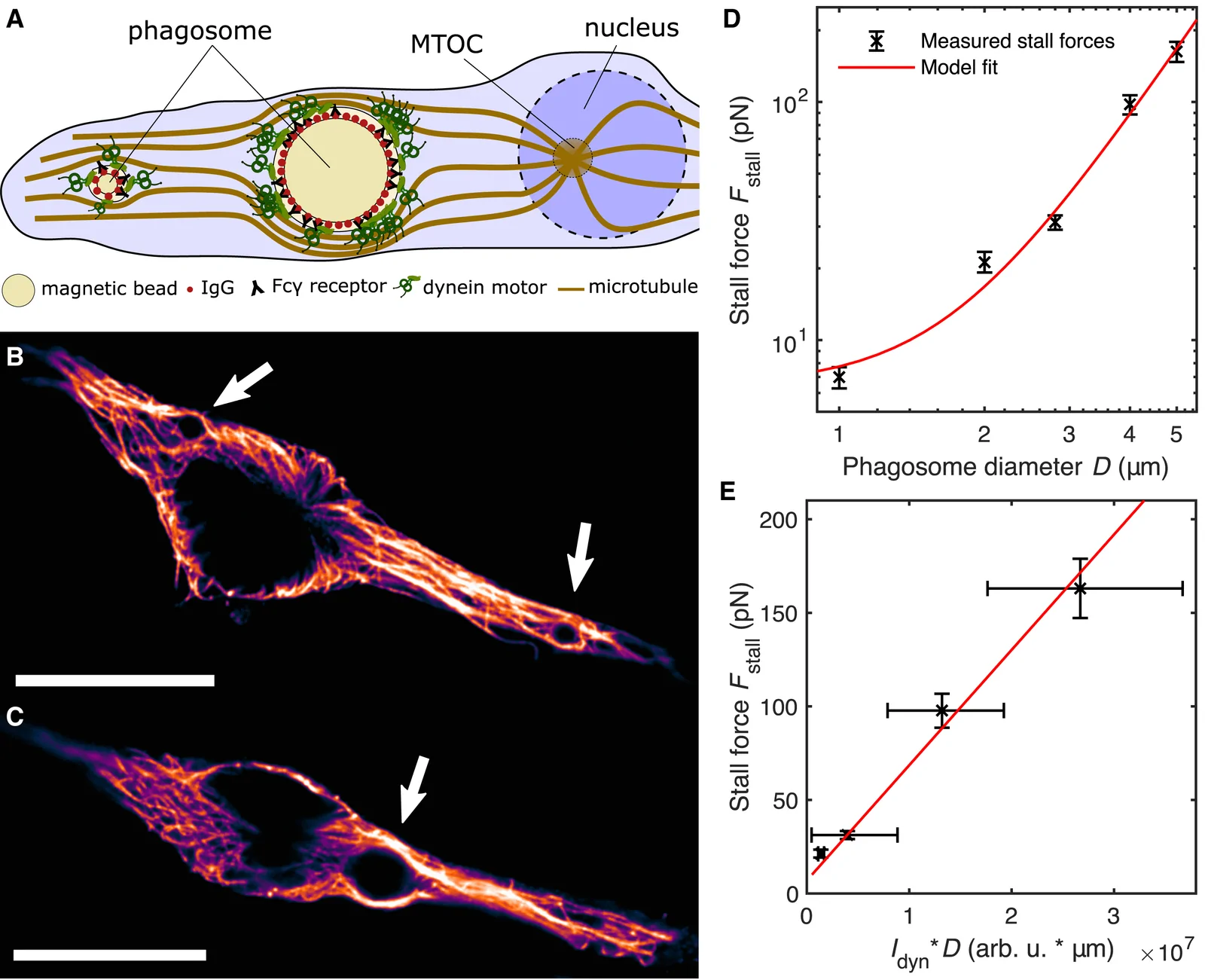
Modeling the displacement of microtubules. Because the number of dyneins attached to a phagosome only increases with $D^{2}$, whereas the stall forces increase with $D^{3}$, a larger fraction of the motors attached to larger phagosomes need to interact with microtubules to explain the observed scaling behavior of $F_{\text{stall}}$. (*A*) The enhanced motor-microtubule interactions can be explained taking the displacement of microtubules by the phagosomes into account. Larger phagosomes displace more microtubules compared to the smaller phagosomes, leading to an increased microtubule density at the phagosomes’ surface. Therefore, more binding sites for dynein are available around the larger phagosomes. (*B* and *C*) In confocal images of the microtubule cytoskeleton of fixed cells (no external magnetic force), small phagosomes (white arrows) with D = 2 *μ*m (*B*) only displaced a few microtubules, barely enhancing the microtubule density on their surface. In contrast, the large phagosomes (white arrow) with D = 5 *μ*m (*C*) displaced many more microtubules in the cytoplasm, leading to a visibly increased microtubule surface density. Scale bar: 15 *μ*m. (*D*) Modeling the displacement of microtubules yields $F_{\text{stall}}\left(D\right)=fD^{3}+c_{m}$ for the stall forces. This simple model provides an explanation for the scaling behavior of the observed stall forces and fits the force data very well. We measured a force density f of (1.3 ± 0.1) pN *μ*m^−3^ and an offset $c_{m}$ of (7 ± 2) pN. (*E*) The same model can be written in terms of the measured dynein intensity on phagosomes (compare Eq. 12). Plotting data and model in this way demonstrates the agreement of our model with both measured stall forces and dynein intensities. Sample size n= 116 (1 *μ*m phagosomes), 73 (2 *μ*m), 67 (2.8 *μ*m), 46 (4 *μ*m), and 44 (5 *μ*m). Error bars represent the 95% confidence interval estimated by bootstrapping.

To resolve this discrepancy, we developed a model that assumes that, in the cytoplasm, organelles displace microtubules from their original positions, increasing the microtubule density on the organelles’ surface. Due to the microtubule displacement, more binding sites are available for dynein at the surface of larger organelles compared to smaller organelles that only interact with a few microtubules (Fig. 4 *A*). This assumption is backed by confocal images of the microtubule cytoskeleton. The smaller phagosomes (2-*μ*m diameter) displaced only a few microtubules, barely increasing the microtubule surface density. However, the large phagosomes (5-*μ*m diameter) displaced many more microtubules in the cytoplasm, leading to a visibly increased microtubule density at their surface (Fig. 4, *B* and *C*). Due to this increased microtubule density, more dyneins can actively contribute to transport forces of larger organelles. Our model assumes, consistent with previous results (70), that active dyneins combine their forces linearly.

In this paragraph, we explain the key ideas of the model. A step-by-step derivation of the model is provided in the supporting material materials and methods. The model assumes that the total number of microtubules that are displaced by a phagosome is proportional to the organelle’s cross-sectional area (constant microtubule area density $\rho_{\text{MT}}$). The displaced microtubules bend around the phagosome, depending on their distance r to the phagosome center. Therefore, the interaction length $l_{\mathrm{int}}\left(r\right)$, on which a microtubule that would cross the phagosome at a given radius r will interact with it, is

(8) $$l_{\mathrm{int}}\left(r\right)=\frac{D}{2}\theta_{\mathrm{int}}\left(r\right)\text{,}$$

with the interaction angle $\theta_{\mathrm{int}}\left(r\right)$. We expect that only the projection of the force of a motor at a given angle θ on the phagosome contributes to the phagosomal stall force. Therefore, we weighted the interaction length with cos(θ) to obtain an effective interaction length $l_{\mathrm{int},\text{eff}}$ of motors that contribute to the stall force. Dyneins with a maximal distance to a microtubule of one motor length $d_{M}$ may interact with this microtubule (Fig. S8 *A*), leading to an effective area of interaction $A_{\mathrm{int},\text{eff}}$ of about $2\;l_{\mathrm{int},\text{eff}}\;d_{M}$ on the phagosome. Summing up all the interaction areas for all the displaced microtubules by integrating over the cross-sectional area of the phagosome $A_{\emptyset }$ yields the effective number of all possible dynein-microtubule interactions contributing to the stall force:

(9) $$N_{\mathrm{int},\text{eff}}\left(D\right)=\rho_{\text{Dyn}}{\;\rho }_{\text{MT}}\int_{A_{\emptyset }}A_{\mathrm{int},\text{eff}}\;dA\text{.}$$

Together with the force of a single dynein $F_{\text{SD}}$ and the model offset $c_{m}$, we modeled the measured stall forces:

(10) $$F_{\text{stall}}\left(D\right)=F_{\text{SD}}\;N_{\mathrm{int},\text{eff}}\left(D\right)+c_{m}=fD^{3}+c_{m}\text{,}$$

with the force density $f={0.5\;F}_{\text{SD}}\;\rho_{\text{Dyn}}\;\rho_{\text{MT}}\;d_{M}$. The constant offset was included because at least one force-producing unit was active during stall-force measurements as a consequence of our measurement method. In addition, to assess the forces predicted by the model with respect to dynein numbers, we can write it to directly include the measured dynein intensity on phagosomes $I_{\text{dyn}}$ by rewriting the dynein surface density $\rho_{\text{Dyn}}$:

(11) $$\rho_{\text{Dyn}}=\frac{N_{\text{Dyn}}}{\pi D^{2}}=\frac{I_{\text{dyn}}}{\pi I_{\text{singledyn}}D^{2}}\text{.}$$

To convert from the dynein number $N_{\text{Dyn}}$ to the dynein intensity $I_{\text{dyn}}$ on phagosomes in our assay, we used the fluorescence intensity of a single dynein complex in our assay, $I_{\text{singledyn}}$. Taken together, this yields

(12) $$F_{stall}=f^{\prime }I_{phago}D+c_{m}\text{,}$$

with the modified force density $f^{\prime }=\frac{0.5\;F_{\text{SD}}\;\rho_{\text{MT}}\;d_{M}}{\pi \;I_{\text{singledyn}}}=\frac{f}{{\pi \;\rho }_{\text{Dyn}}\;I_{\text{singledyn}}}\text{.}$

This model fits the experimentally determined stall forces very well and provides an explanation for how the dynein numbers increasing with $D^{2}$ generate stall forces increasing with $D^{3}$ (Eq. 10; Fig. 4 *D*). In addition, we plotted the modeled stall forces against the dynein intensities measured on the isolated phagosomes (Eq. 12; Fig. 4 *E*). There is a clear agreement of the measured forces and dynein intensities with our model.

We measured a force density f of (1.3 ± 0.1) pN *μ*m^−3^ and an offset $c_{m}$ of (7 ± 2) pN. From the fitted force density f, we estimated the cytoplasmic microtubule density $\rho_{\text{MT}}$ of (11 ± 8) microtubules *μ*m^−2^ for a dynein surface density $\rho_{\text{Dyn}}$ of (3 ± 1) dyneins *μ*m^−2^, a single dynein stall force $F_{\text{SD}}$ of 1–4 pN (11,12,13,14,20), and a dynein length $d_{M}$ of 65 nm (71).

## Discussion

Understanding how large groups of molecular motors interact with each other and the cytoskeleton to produce large collective forces is of major biological and biomedical importance. Here, we demonstrated that the transport of large organelles is coordinated by up to hundreds of dyneins that cluster into many teams and likely interact with multiple microtubules. The involvement of such large numbers of dyneins was measured in two independent ways: first, the dynein-inhibition experiments showed that this motor is mainly responsible for the retrograde transport. The combination with the direct measurement of the transport forces of up to 160 pN (95% confidence interval: 150–180 pN) shows that such large numbers of motors need to be involved. Second, the motor numbers of the phagosomes were directly quantified by stepwise photobleaching.

Clustering of dyneins into teams may be caused by the enrichment of dynein on lipid rafts (28) and by dynamic clustering under load (29). This clustering of dynein may be a general mechanism of dynein’s collective action (15,28,29), as, within a single dynein team, the load is distributed between individual motors via the elastic lipid membrane of the organelles (28). The ability of dyneins to adjust their step length and frequency of backstepping under a resisting load (12,23) permits leading dyneins to slow down while trailing dyneins can catch up, allowing them to combine their individual forces (11,28). Additionally, their high binding affinity to microtubules allows the dyneins to stay attached even at high loads (11,24,25,26).

We hypothesize that similar mechanisms may mediate the collective force generation of multiple dynein teams. Forces may be transmitted between dynein teams via the phagosomal membrane. Leading dynein teams may thus experience an increased resisting force, on average slowing down the motion of individual dyneins in such a team. In contrast, trailing dynein teams may bear less load, allowing the individual dyneins in these teams to increase their step length and catch up. The high binding affinity of dyneins to microtubules may prevent dynein teams that momentarily experience very high loads from falling off. In this way, the dynein teams may evenly share the load between them and produce high forces collectively. This mechanism of collective action of dynein teams may also affect their net transport velocity: to enable trailing dynein teams to catch up, leading dynein teams need to slow down or halt their motion, which may, on average, decrease the cargo velocity. Consistently, we noticed that the average free-transport velocity of the intracellular cargoes was about 25 nm s^−1^, which is in agreement with previous studies on large intracellular cargoes with diameters of a few micrometers (38) and much lower than the velocity of 500–1000 nm s^−1^ that was measured for dynein in vitro (14,23,49). Additionally, individual dyneins that were studied in vitro with no or small cargoes experience a much smaller drag compared to the groups of dyneins on phagosomes in vivo and can therefore move faster. This is evident from in vitro measurements of the retrograde transport velocity of purified phagosomes and beads decorated with dyneins, which move with velocities of ca. 200–1200 nm s^−1^ (3,14,34). However, these experiments are not directly comparable to our measurements, since the reconstituted organelles were placed on top of straight microtubules on a glass coverslip. In this situation, as suggested by the model of Rai et al. (28), only one team of dyneins can be active due to the limited area of interaction with the microtubules, and the load is not shared between multiple dynein teams. Furthermore, for small phagosomes (1-*μ*m diameter) transient velocities of more than 100 nm s^−1^ have been documented in vivo (72) on timescales that are smaller than the ones investigated here.

Because of the ability of dynein teams to produce forces collectively and their size-dependent interactions with the microtubule cytoskeleton, we observed that the forces during retrograde organelle transport scale with approximately $D^{3}$. In the power-law fit of the stall forces, we included an offset c (experimental data) or $c_{m}$ (model), since at least one force-producing unit must be active to observe retrograde organelle transport. Likely, this minimal force-producing unit consists of two dyneins due to the pairwise recruitment of dynein to the phagosomes via the Rab7/RILP complex (11,73,74). Therefore, an offset of 2–8 pN is expected (11,12,13,14). We observed an offset of 6–7 pN, consistent with reports of a stall force of 3–4 pN for a single dynein, indicating that cargo adaptors were involved in the retrograde transport (14,18,75). Our observation of a minimal transport force agrees with earlier reports about organelle stall forces in vivo. For even smaller phagosomes (diameter of 0.74 *μ*m) stall forces of 6–10 pN were measured (11), and, for lipid droplets (diameter of 0.4–0.6 *μ*m), average stall forces of 2–10 pN (75,76) were found. However, the stall forces were broadly distributed, with individual forces as low as 1–2 pN. These low forces measured in optical-tweezer experiments likely were related to the motors’ processivity, if the stall force is not reached before the motors detach (20). These observations together with the results presented in this study may potentially suggest that the scaling behavior of retrograde transport forces with cargo size is divided into distinct regimes: for small cargoes (≲1 *μ*m), the observed stall forces seemed to be independent of cargo size (11,75,76). In this regime, dyneins in only one team may interact with one microtubule (28) and combine their forces linearly, modulated by force-dependent motor detachment (70). Steric interactions of dyneins within such a dynein team may potentially limit the collective force production, leading to a size-independent stall force. A similar effect has been observed for myosins and kinesins, where only a limited number of motors can simultaneously engage with an actin filament or microtubule (77,78).

In contrast, for cargoes with diameters of a few micrometers, microtubule displacement and bending will play a role, as suggested by our model and data. In this regime, the transport force also linearly depends on the number of active dyneins. However, this number additionally increases with the increasing microtubule density on the phagosomes’ surface due to their displacement and bending. This effectively leads to the observed scaling exponent of $F∼D^{3}$.

We found that the transport forces decreased by 50% after a 50% reduction of the expression level of dynein and after a 50% reduction of the ATPase activity of dynein. These results indicate that dynein is the main driver for the observed transport processes. Inhibiting the expression or activity of dynein may affect kinesin’s activity as well, which is known as the “paradox of co-dependence” (79,80). How groups of opposing molecular motors coordinate their movement to drive bidirectional transport was investigated by numerous in vitro and in vivo studies (11,21,28,30,32,33,34,80,81). Consistent with our observations, some of these studies reported that kinesins did not functionally interfere with dynein during retrograde runs (21,30,33,81).

Additionally, we considered the possibility that other contributions to the observed transport forces might originate from the elastic deformation of the cell cortex by the phagosomes. We found that these elastic forces scale linearly with the phagosome diameter D and can therefore not explain the observed $D^{3}$ scaling of the retrograde transport forces. Our findings show, in agreement with previous results (67,68), that these elastic forces start to dissipate on timescales longer than ∼10 s. Since in our measurements the time between reaching the maximal magnetic force and the onset of retrograde transport is much longer than this time, potential elastic deformations of the cell cortex by the phagosomes likely did not contribute to the measured stall forces. Taken together, this shows that dynein is predominantly responsible for the retrograde transport.

There is plenty of evidence for the collective force generation of dyneins, which can work together in large teams with more than 10 individual motors (11,28,32). Our study further supports these observations of dyneins’ collective action, suggesting that multiple dynein teams work together to produce very large collective forces of up to 300 pN, indicating the activity of ∼100 motors. However, in the case of kinesins, the picture is less clear. There are several theoretical studies demonstrating that multiple kinesins can, in principle, combine their forces (82,83). These studies are backed by experimental evidence, which shows that, for organelles such as latex bead compartments and lipid droplets, 2–3 kinesins may combine their forces to overcome the collective force of ∼10 dyneins (32,84). In intraflagellar trains, average anterograde transport forces of 21 pN were observed, indicating the collective action of more than four kinesins (31). Moreover, in microtubule gliding assays, much higher forces were observed, indicating the combined action of many kinesins (85). However, microtubule gliding assays and intraflagellar transport are unique systems that may not be directly comparable to the transport of organelles in the cytoplasm, which was studied here. We are not aware of any evidence observing the cooperation of many (∼10–100) kinesins during intracellular organelle transport, as was observed for dyneins.

In the light of these studies, it has to be considered that kinesin’s mechanochemistry differs from that of dynein, which makes it seem unlikely that large numbers of kinesins can work collectively in teams in the same way as dyneins do. The detachment rate of dynein may decrease in the superstall regime (catch bond) (11,86), remain constant, or slightly increase under load (ideal bond, slip-ideal bond) (25,26,87), depending on the design of the study. Furthermore, the tenacity of dynein motor teams is proportional to the motor copy number in the sub- and superstall regime (11). In contrast to dynein, kinesin’s detachment rate increases in the superstall regime with increasing load (slip bond) (86,88,89). Additionally, neither kinesin’s processivity nor its velocity increase with the motor number (84), and the load is usually not shared between multiple kinesins (90). Consequently, the tenacity of kinesins does not increase with increasing motor number (11). However, it is important to note that the mechanochemical properties of molecular motors measured in vitro can be affected by factors like the assay geometry and the stiffness of the optical trap used in the experiments (20,91). To fully understand how dynein and kinesin combine their forces, more detailed studies are needed to connect their individual mechanochemical characteristics with their ability to produce forces collectively.

Overall, the ability of dyneins to produce forces collectively may potentially restrict the ability of kinesins to oppose multiple teams with more than 100 dyneins that drive the retrograde transport of large organelles (23,28,32,33,92). In the case of small organelles, however, we show that only a few dyneins are active, generating forces in the order of 10 pN, which can be balanced by 2–3 kinesins (32,93,94). This may explain why large 3-*μ*m phagosomes were transported very persistently toward the perinuclear region, whereas small 1-*μ*m phagosomes engaged in a bidirectional, tug-of-war-like motion with frequent phases of persistent anterograde transport (38). Similarly, in fibroblasts, it was observed that larger latex-bead compartments were located close to the nucleus after intracellular transport, whereas smaller particles remained in the cell periphery (95). These findings agree with the hypothesis of Rai et al. (28), which suggests that the clustering of dyneins on late phagosomes enhances their collective force generation, allowing them to overcome the anterograde forces of 2–3 kinesins and enable more persistent transport toward the perinuclear region.

Taken together, these results seem to suggest a size-dependent mechanism of intracellular cargo transport in which dyneins, due to their strong ability to produce large forces collectively, can outcompete kinesins on larger organelles, leading to more persistent and less bidirectional retrograde transport. In this study, we investigated this phenomenon for phagosomal transport, since phagocytosis enables the reliable generation of organelles of a very defined size and shape (96). However, not all cellular cargoes have the same properties as the phagosomes used in this study. For example, softer organelles might be subjected to deformations due to stresses from the microtubules and large forces applied by motor teams (97), unlike our phagosomes. Nevertheless, the mechanism we propose here might be applicable to a wider variety of organelles, because it only depends on basic geometric assumptions (constant motor density, displacement and bending of microtubules). Size-dependent interactions of organelles with microtubules possibly contribute to the transport of a range of organelles, as even membrane-less organelles displace the microtubule cytoskeleton in a size-dependent manner (98). A possible application outside of phagocytosis might be the intracellular positioning of nuclei, which also requires the collective action of large numbers of dyneins and kinesins (5,36). This size-dependent regulation of bidirectional cargo transport may complement biochemical mechanisms, which include, for example, cues embedded in the microtubule tracks, such as microtubule-associated proteins, posttranslational microtubule modifications, and microtubule bundling (99,100), or the selective activation of individual motor types, which is linked to the coordination of different cargo adaptors (100).

Our results together with the theoretical model also highlight the role of the microtubule cytoskeleton for the generation of large collective forces. From our model, we derived a prediction for the cytoplasmic microtubule density $\rho_{\text{MT}}$ of (11 ± 8) microtubules *μ*m^−2^. This value is well within the range of previous reports, underlining the validity of our model. In neutrophils, a cytoplasmic microtubule density of 1 microtubule *μ*m^−2^ was observed (101), in neuron dendrites 50–70 microtubules *μ*m^−2^ were found (102,103), and in axons up to 150 microtubules *μ*m^−2^ (104). There is a remarkable coincidence between the microtubule density and the range of directed organelle transport: in neutrophils and macrophages, distances of only 1–10 *μ*m need to be covered, in dendrites 10–100 *μ*m (105), and in axons 1 mm to 1 m (106). Our model suggests that the number of active dynein teams depends on the microtubule density, indicating that the cytoplasmic microtubule density may be an important factor to regulate the robustness and persistence of intracellular organelle transport.

## Conclusions

The experimental results and theoretical model we present in this study suggest that dynein’s inherent ability to generate forces collectively and its interactions with the microtubule cytoskeleton may both be important to drive fast intracellular transport of organelles of different sizes. Since processes such as the degradation of pathogens by phagocytes or the clearance of particulate contaminants like microplastics rely on the efficient transport of organelles spanning orders of magnitude in size, these findings are relevant not only for fundamental cell biology but also from a biomedical perspective.

## Data and code availability

All data needed to evaluate the conclusions in the paper are present in the paper and/or the supporting material. All code used in the evaluation of the data and additional raw data are available from the corresponding author upon request.

## Acknowledgments

We would like to acknowledge and honor Erich Sackmann, whose pioneering contributions to cell biophysics and magnetic force measurement techniques have greatly influenced our work. We thank Thomas Scheibel and Hendrik Bargel for their support with the TIRF setup and Matthias Weiss and Konstantin Speckner for their assistance with the confocal images of the microtubules. This work received funding from the 10.13039/501100001659Deutsche Forschungsgemeinschaft (DFG, German Research Foundation)—project number 391977956—SFB 1357 and DFG GZ: INST 91/367-1 FUGG. S.W. and D.E.G. were supported by the 10.13039/501100008848Elite Network of Bavaria (Study Program Biological Physics). S.W., D.E.G., W.G., and J.R. were supported by the University of Bayreuth Graduate School.

## Author contributions

H.K., A.G.H., and S.W. designed the research. S.W., C.S., D.E.G., and M.M.K. performed the experiments and evaluated the data with support from A.R.C. and J.R. H.K. and S.W. developed the theoretical model. W.G. developed and implemented the setup and procedures for the magnetic force measurements. S.W. wrote the manuscript. All authors discussed the results and revised the manuscript.

## Declaration of interests

The authors declare no competing interests.

## Supporting material

Document S1. Figures S1–S16, Tables S1–S4, and supporting material appendix

Document S2. Article plus supporting material
