# Supplementary material for: Many dynein teams collectively generate high forces during the transport of large organelles
Source: Biophys J. 2025 Sep 10;125(12):3009–24. doi: 10.1016/j.bpj.2025.09.012 (PMC13507173; doi:10.1016/j.bpj.2025.09.012)
Supplement: Supplementary file 1 [file mmc1.pdf]

Biophysical Journal, Volume *125*

Supplemental information

Many dynein teams collectively generate high forces during the transport of large organelles

Simon Wieland, Christina Steininger, David E. Gitschier, Marius M. Kaiser, Wolfgang Gross, Abdullah R. Chaudhary, Jana Ritschar, Christian Laforsch, Adam G. Hendricks, and Holger Kress

Supporting Information for

Many dynein teams collectively generate high forces during the transport of large organelles

Simon Wieland[1,2], Christina Steininger[1], David E. Gitschier[1], Marius M. Kaiser[1], Wolfgang Gross[1], Abdullah R. Chaudhary[3], Jana Ritschar[4], Christian Laforsch[2], Adam G. Hendricks[3], Holger Kress[1,*]

[1] Biological Physics, University of Bayreuth, Bayreuth, Germany
[2] Animal Ecology I, University of Bayreuth, Bayreuth, Germany
[3] Department of Bioengineering, McGill University, Montreal, Canada
[4] Laboratory of Molecular Parasitology, University of Bayreuth, Bayreuth, Germany

*Corresponding Author: Holger Kress

Email: holger.kress@uni-bayreuth.de

This PDF file includes:

SI Appendix
Figures S1 to S16
Tables S1 to S4
SI References

SI Appendix

Calibration of the magnetic tweezers. We calibrated the force of the magnetic tweezers on the magnetic beads by suspending ca. 1.6×10^6 beads in 1 ml of a 75%/25% (v/v) glycerol-water solution. The viscosity of this solution was calibrated in the temperature range of 20-25°C using an Ostwald viscosimeter. It followed approximately a linear relation

$$\eta(T) = 0.103\ \mathrm{Pa\ s} - 0.0026\ \mathrm{Pa\ s\ °C^{-1}} \times T,$$

with the solution's temperature T in °C. The uncertainty of the viscosity s_η was about 5%. The tip of the magnetic tweezers was immersed in 150 µl of the calibration solution, placed 100 µm above the coverslip and brought into focus. Then, the coil current was turned on and the beads started moving towards the tip of the magnetic tweezers (Figure S9 A). From the bead trajectories, their velocity was determined as previously described (1). Briefly, we detected the particles in each frame with a peak-finding algorithm. Using the tracking algorithm by Crocker and Grier (2), the individual particle positions were linked to trajectories. The instantaneous particle velocity $\boldsymbol{v}$ was then calculated for each particle position along the trajectories over intervals of three frames. Here, only particles in a range of ±40° from the tip axis were considered to ensure radial symmetry of the magnetic gradient field. The tip of the magnetic tweezers was detected by automated image analysis and the shortest distance d between the particle and the tip surface was calculated for each particle position. Using Stokes' law of friction, we obtained the force-distance relationships for the individual particle types:

$$F_{\mathrm{MT}}(d) = 6\pi\, \eta\, R\, v(d)$$

Heuristically, we fitted the force-distance relationship with a power law to obtain the calibration curves:

$$F_{\mathrm{MT}}(d) = \frac{F_0}{\left(\frac{d - r_0}{d_0}\right)^c}$$

The parameter d_0 was set to 25 µm. The fit results for F_0, r_0, and c are shown in Table S2 for all particle types. Three factors contributed to the uncertainty of the calibration curve: velocity uncertainties due to tracking errors, variations in size and magnetic moment of the individual particles, and the uncertainty of the viscosity. Both tracking errors and variations of the beads' size and magnetic moment resulted in variations of $F_{\mathrm{MT}}(d)$. To determine their magnitude, we fitted an exponential function to the absolute values of the differences between the data points and the fitted calibration curve, averaged over intervals of 3 µm in the parameter d:

$$s_{\mathrm{scatter}}(d) = a \exp\left(-\frac{d}{b}\right) + k$$

The fit results for the parameters a, b, and k are shown in Table S2. Together with the uncertainty of the viscosity, the total uncertainty of the calibration curve is

$$s_F(d) = \left[\left(\frac{\partial F(d)}{\partial \eta} \times s_\eta\right)^2 + s_{\mathrm{scatter}}^2(d)\right]^{\frac{1}{2}} = \left[\left(\frac{s_\eta}{\eta} \times F(d)\right)^2 + s_{\mathrm{scatter}}^2(d)\right]^{\frac{1}{2}}$$

$$= \left[\left(0.05 \times F(d)\right)^2 + s_{\mathrm{scatter}}^2(d)\right]^{\frac{1}{2}}.$$

The resulting calibration curves are shown with their respective uncertainties in Figure S9 B-F.

Western blot protocol. After transfection, the medium of the cells was replaced by 100 µl of homogenization buffer (HB, consisting of motility assay buffer (MAB): 10 mM PIPES, 50 mM K-Acetate, 4 mM MgCl2, and 1 mM EGTA; pH 7.0; with 10% sucrose, 1x cOmplete EDTA-free

protease inhibitors (Sigma Aldrich, Darmstadt, Germany), 10 µM ATP, and 30 µM DTT), cells were chilled on ice, scraped from the bottom of the wells, and transferred to a 500 µl Eppendorf tube. The cells were homogenized on ice in an ultrasound bath 3x for 10 s. Then, 20 µl of 6x Laemmli buffer was added, and the lysate was heated to 70°C for 10 min. The lysate was stored at -20°C.
Prior to blotting, the lysates were thawed and heated to 95°C and centrifuged at 17,000 g for 30 s to remove debris. A precast SDS-PAGE gel (10% Mini-PROTEAN® TGX™ Precast Protein Gels, BioRad Laboratories Inc., Hercules, CA) was inserted into the electrophoresis cell (Mini-PROTEAN Tetra cell, BioRad Laboratories Inc., Hercules, CA) and filled with running buffer (0.29% w/v Tris, 1.44% w/v Glycine, 0.1% SDS w/v in MilliQ water). To each pocket of the gel, 20 µl of the lysate were added. In one pocket, 5 µl of a protein ladder (PageRuler Plus Prestained Protein Ladder, Thermo Fisher Scientific, Waltham, MA) were added. Electrophoresis was started for 15 min at 80 V, and was continued at 120 V for approximately 1 h, until the protein ladder was spread out over the whole gel. Before the western blot, the nitrocellulose membrane (Amersham Protran 0.45 µm NC Nitrocellulose Blotting Membrane, GE Healthcare, Chicago, IL), filter paper (Mini Trans-Blot Filter paper, BioRad Laboratories Inc., Hercules, CA), and sponges were soaked thoroughly in the transfer buffer (0.29% w/v Tris, 1.45% w/v Glycine, 20% v/v methanol in MilliQ water). Then, the blot was assembled, inserted into the electrophoresis cell, filled with transfer buffer, and ran at 100 mA overnight at room temperature.
After blotting, the nitrocellulose membrane was washed in PBS. To verify successful blotting, proteins were stained using Ponceau S (Sigma Aldrich, Darmstadt, Germany). The Ponceau dye was then washed out with MilliQ water. To prevent unspecific binding of the antibodies, the membrane was incubated 2x 15 min in PBS-T (PBS with 0.1% Tween-20) with 5% w/v milk powder. In between and after the incubation, the membrane was washed 3x with PBS-T. The primary antibodies (mouse anti dynein intermediate chain: Invitrogen Dynein Monoclonal Antibody (74.1) MA1-070, Thermo Fisher Scientific, Waltham, MA. mouse anti α-tubulin as loading control: alpha-Tubulin (DM1A) Mouse mAB, Cell Signaling Technology, Cambridge, UK) were diluted 1:1000 in PBS-T containing 5% w/v milk powder. The membrane was incubated for 1h at room temperature with the primary antibodies, then washed 3x with PBS-T. The secondary antibody (goat anti mouse, horseradish peroxidase: Immun-Star Goat Anti-Mouse (GAM)-HRP Conjugate #1705047, BioRad Laboratories Inc., Hercules, CA) was diluted 1:1000 in PBS-T containing 5% w/v milk powder and incubated for 1h at room temperature with the membrane. Finally, the membrane was washed 3x with PBS-T and then 3x with PBS. The membrane was then transferred to a transparent plastic bag. 400 µl of the chemiluminescence substrate (Clarity ECL Western Blot Substrate, BioRad Laboratories Inc., Hercules, CA) were prepared and homogeneously distributed over the nitrocellulose membrane. The bag was tightly sealed. The chemiluminescence signal was recorded using a biomolecular imager (ImageQuant LAS 4000, GE Healthcare, Freiburg, Germany).

Dynein immunofluorescence protocol. For the immunofluorescent labeling of dynein on the phagosomes, small flow channels were assembled with the treated coverslips and microscopy slides using Parafilm and vacuum grease. The channels were rinsed 3x with MAB. Then, 15 µl of the isolated phagosomes were suspended in 60 µl of MAB and flown into the flow channel. The phagosomes were left for 1 h to settle on the hydrophobic coverslip. After washing 3× with MAB, 60 µl of 5 µM Pluronic F-127 (Sigma Aldrich, Darmstadt, Germany) was added to block the coverslip for 30 min. After washing 3× with MAB, phagosomes were fixed for 15 min with 4% paraformaldehyde (PFA) and then washed 2x with MAB-T (MAB with 0.1% Tween 20) and 1x with MAB before overnight incubation at 4°C with the primary antibody (Invitrogen Dynein Monoclonal Antibody (74.1) MA1-070, Thermo Fisher Scientific Inc., Waltham, MA). The next day, samples were washed 2x with MAB-T and 1x with MAB and incubated for 45 min at RT with the secondary antibody (Invitrogen Goat Anti-Mouse IgG, DyLight 488 conjugated, Thermo Fisher Scientific Inc., Waltham, MA). After washing 2x with MAB-T and 1x with MAB, the flow chamber was sealed with vacuum grease. Samples were imaged on the same day. To ensure specificity of the primary antibody, western blots of the total cell lysate and isolated phagosomes were performed. Furthermore, suitable control samples were always prepared (phagosomes stained without primary antibody to ensure specificity of the secondary antibody; unphagocytosed beads with primary and secondary antibody and only secondary antibody). There was a slight fluorescent signal from the unphagocytosed beads stained with primary and secondary antibody, indicating a weak unspecific

binding of the primary antibody to the carboxylate beads. There was no notable unspecific binding of the secondary antibody.

Replication of the dynein immunofluorescence assay. To validate our dynein immunofluorescence assay, we completely replicated our experiments by producing a new batch of isolated phagosomes and labeled dynein with different types of primary and secondary antibodies. Additionally, we labeled kinesin-2.

J774 cells were grown as described above in 10 cm cell culture dishes until they reached 80% confluency. Carboxylated beads (D = 2 µm, 4 µm, 5 µm) were washed 3× in PBS, and 100 µl of each stock solution was suspended in 10 ml of D10-, with 2.5 ml added per dish (four dishes per bead size). Cells were incubated for 60 min to enable phagocytosis, and subsequently the phagosomes were isolated and purified as described above. However, instead of homogenizing the cells with a syringe, we used a dounce homogenizer (VWR).

For the immunofluorescence assay, we followed the same procedure as before but used a different set of antibodies: Dynein HC Antibody, Santa Cruz, Catalogue Number sc-9115 (primary, 1:500 dilution, incubated at 4°C overnight), and Goat Anti-Rabbit IgG – Atto 488, Sigma Aldrich, Catalogue Number 18772 (secondary, 1:1000 dilution, incubated for 45 min at room temperature). We also labeled kinesin-2 using a monoclonal primary antibody (Mouse Anti KIF3A, ab24626, abcam, directly conjugated with Alexa-568) diluted to 1:500 and incubated at 4°C overnight.

Samples were imaged using an inverted light microscope (Nikon Eclipse Ti-E, Nikon, Japan) equipped with a 100× objective (Apo TIRF 100× OIL DIC N2). The sample was illuminated either with a diascopic brightfield lamp, or with fluorescence excitation lasers (488 nm or 561 nm, respectively). Images were captured with an EMCCD camera (Andor DU-897, Oxford Instruments, UK). Dynein samples were imaged at 12 mW laser power (488 nm) and an exposure time of 500 ms. Kinesin-2 samples were imaged at 10 mW laser power (561 nm) and an exposure time of 500 ms.

An overview of the workflow, including experimental procedures, measured parameters, and the corresponding control experiments is given in Figure S4. The results of the phagosome isolation and immunofluorescence experiments and replication experiments are given in Figure S12.

Determining the unitary step size in the photobleaching experiments. The photobleaching curves were fitted with a piecewise constant function using a step finding algorithm. The resulting step sizes correspond to the intensity of one or multiple fluorophores. Examples of photobleaching curves are shown in Figures S5 and S6. In Figure S5 A photobleaching curves measured in the epifluorescence mode of the microscope on samples with labelled phagosomes are shown. In the phagosome samples, photobleaching curves of small fluorescent spots on the coverslip were evaluated. In larger objects, e.g., complete phagosomes, where the total intensity is 100× to 1000× higher than the unitary step size it is difficult to resolve individual steps, since the variance of the signal (scaling with the square root of the number of fluorophores) became much larger than the intensity of a single fluorophore. The analyzed fluorescent spots, possibly originating from individual antibodies bound unspecifically to the coverslip, had much lower integrated intensities than the phagosomes, making it easier to resolve individual steps. On average, these fluorescent spots had 13 unitary steps. From the unitary step size calibrated with these fluorescent spots, we extrapolated the number of fluorophores on the phagosomes. In Figure S5 B photobleaching curves of samples with individual antibodies measured in the TIRF mode of the microscope are shown. To avoid overfitting, only downward steps > 70 arb. u. were considered for the estimation of the unitary step size.

From the distribution of the step sizes the intensity of a single fluorophore was determined by fitting a Gaussian mixture model (3, 4):

$$\mathrm{GMM}(k, \mu_i, \sigma_i) = \sum_{i=1}^{k} P_i \mathcal{N}_i(\mu_i, \sigma_i),$$

with k normally distributed components $\mathcal{N}_i$ with mean value μ_i, variance σ_i, and weight P_i. The optimal number of components k was chosen to minimize the Bayesian information criterion of the

fit (3, 5). The step size distributions and fitted Gaussian mixture models for epifluorescence and TIRF mode are shown in Figure S5 C and D. The unitary step size, corresponding to the intensity of a single fluorophore, was calculated from the fitted Gaussian mixture model:

$$I_{\mathrm{unitary}} = \sum_{i=1}^{k} \frac{1}{i} P_i \mu_i.$$

For each step size distribution, the Gaussian mixture model was fitted 1000× with random starting points, until the mean of the unitary step size converged. The uncertainty of the unitary step size was calculated from the standard deviation of the unitary step size distribution. We measured a unitary step size of (160 ± 30) arb. units in epifluorescence mode and a unitary step size of (235 ± 50) arb. units in TIRF mode.

Determining the degree of labeling (DOL) of the secondary antibodies. To determine the number of fluorophores coupled to a single secondary antibody, we diluted the secondary antibodies 1:10,000 in 60 µl of MAB, flowed them into a sample chamber and let them attach to the coverslip overnight at 4°C (Figure S5 E). We imaged them in the TIRF mode, measured the intensity of each spot on the coverslip, and corrected the measurements for background. The number of fluorophores per secondary antibody was then calculated as:

$$N_{\mathrm{sAB}} = I_{\mathrm{sAB}} / I_{\mathrm{unitary}}^{\mathrm{TIRF}}$$

On average, we found that 11 ± 1 fluorophores were bound to a single secondary antibody (Figure S5 F). To verify our method, we compared this result to the spectroscopically measured degree of labeling. Using an UV-Vis spectrophotometer (Genesys 10S UV-Vis Spectrophotometer, Thermo Fisher Scientific, Waltham, MA), we measured the absorbance of the antibody solution at 280 nm A_{280} to determine the protein concentration and the absorbance at 495 nm A_{495} to measure the concentration of fluorophores (Table S3). The degree of labeling is then:

$$\mathrm{DOL} = \frac{\epsilon_{\mathrm{IgG}} A_{495}}{(A_{280} - A_{495} \times \mathrm{CF}) \epsilon_{\mathrm{DyLight-488}} l_{\mathrm{d}}},$$

with ϵ_{IgG} and $\epsilon_{\mathrm{DyLight-488}}$ the extinction coefficients of IgG at 280 nm and the DyLight-488 fluorophore at 495 nm, the sample path length l_{d}, and a correction factor CF accounting for the absorption of the fluorophore at 280 nm. We measured a degree of labeling of 10 ± 1 fluorophore per single secondary antibody, aligning well with the degree of labeling measured by stepwise photobleaching, thus verifying our photobleaching approach. The degree of labeling that we measured by photobleaching and spectroscopy is technically possible, as there are up to 90 lysines on a single antibody, to which an amine-reactive dye such as DyLight-488 could couple (6, 7). This makes an even larger degree of labeling in principle possible, and likely also explains the variation of the number of fluorophores per secondary antibody that we observed.

Immunofluorescence of the microtubule cytoskeleton. After the cells were fixed with PFA, they were washed 3x and then permeabilized with PBS containing 0.1% Triton-X for 10 min. After washing the cells 3x, they were incubated for 2h at RT with the primary antibody (alpha-Tubulin (DM1A) Mouse mAB, Cell Signaling Technology, Cambridge, UK), washed 3x with PBS-T, incubated for 45 min with the secondary antibody (Invitrogen Goat Anti-Mouse IgG, DyLight 488 conjugated, Thermo fisher Scientific Inc., Waltham, MA), and washed 3x in PBS-T. Finally, they were washed 1x with PBS and transferred to ultrapure water. The coverslips were then mounted on microscopy slides using Fluoromount G (Thermo Fisher Scientific Inc., Waltham, MA).

Modeling of elastic forces due to deformations of the cell cortex. To quantify the potential contributions of cell cortex deformations to the transport forces, we modeled the deformation to estimate the magnitude and scaling behavior of these elastic forces.

In scanning electron micrographs of cells which internalized the magnetic beads, we observed that the beads slightly deformed the outer cortex of the cells, depending on their distance to the nucleus (Figure S7 A). For our model of the deformation of the cell cortex, we assumed that the cell has a wedge-like shape, and the beads deform the cell cortex of the cell if their diameter is larger than the height of the cell at their respective position (Figure S7 B). Heuristically, we assumed that this deformation has the shape of a cosine which smoothly transitions to the undeformed cell at its edges and has a radius of curvature that is equal to the bead radius at its maximum. A sketch of the geometry is shown in Figure S7 C. This model of the deformation shown in Figure S7 B resembles the deformations observed in the scanning electron micrograph.
For a bead with radius $R = D/2$ which is at position x_{B} along the x axis, the deformation of the cell cortex along the x' axis can be written as:

$$f(x') = h\left[\frac{1}{2}\cos\left(\frac{1}{a}(x' - x'_{\mathrm{B}})\right) + \frac{1}{2}\right],$$

where h is the height of the deformation perpendicular to the undeformed cortex, and πa the radius of the deformed area of the cell cortex. The deformation height h depends on the position of the bead x_{B}:

$$\begin{aligned} h &= R - h' \\ &= R - \cos(\theta)\, h'' \\ &= R - y_{\mathrm{B}}\cos(\theta) + R\cos(\theta) \\ &= R - x_{\mathrm{B}}\tan(\theta)\cos(\theta) + R\cos(\theta) \\ &= R(1 + \cos(\theta)) - x_{\mathrm{B}}\sin(\theta). \end{aligned}$$

To ensure a smooth transition of the deformation into the undeformed cell, the interval of the deformation ranges from $x'_0 = x'_{\mathrm{B}} - \pi a$ to $x'_1 = x'_{\mathrm{B}} + \pi a$. To determine the width of the deformation a, we use the additional condition that the radius of curvature of the deformation at $x' = x'_{\mathrm{B}}$ must be equal to the bead radius R. The radius of curvature of a function $f(x')$ is defined as

$$r(x') = \left|\frac{(1 + f'(x')^2)^{\frac{3}{2}}}{f''(x')}\right|,$$

where $f'(x')$ and $f''(x')$ are the first and second derivatives of $f(x')$. At the position of the bead the radius of curvature of the deformation therefore is

$$r(x' = x'_{\mathrm{B}}) = \frac{2a^2}{h} = R.$$

With the deformation height h, this yields

$$a = \left(\frac{1}{2}R[R(1 + \cos(\theta)) - x_{\mathrm{B}}\sin(\theta)]\right)^{\frac{1}{2}}.$$

We assumed that the deformation of the cell cortex is rotationally symmetric around x'_B. Therefore, to calculate the area of the deformed cell cortex in the interval $[x'_0, x'_1]$, we used Pappus' centroid theorem. The arc length L of the function $f(x')$ in the interval from x'_{B} to $x'_{\mathrm{B}} + \pi a$ cannot be calculated analytically and was therefore determined numerically. The distance d of its geometric centroid to the axis of rotation is $d = \frac{1}{2}\pi a$ due to the function's symmetry. In total, the area of the deformed actin cortex is

$$A_{\mathrm{deformation}} = 2\pi d L$$

The corresponding area of the undeformed cell cortex in the interval $[x'_0, x'_1]$ is:

$$A_{\mathrm{undeformedCell}} = \pi^3 a^2$$

The total change in the area of the cell cortex due to the deformation caused by the bead is therefore:

$$\Delta A = A_{\mathrm{deformation}} - A_{\mathrm{undeformedCell}}.$$

With the known tension of the cell cortex of J774A.1 cells σ = 140 ± 20 pN μm^{-1}, which includes tensions of the actin cortex as well as the plasma membrane (8), we can calculate the energy required for a deformation of ΔA of the cell cortex:

$$W_{\mathrm{deformation}} = \sigma \Delta A.$$

The force which pushes the bead along the x axis is the spatial derivative of this energy:

$$F_{\mathrm{Actin}} = -\frac{\partial}{\partial x_{\mathrm{B}}} W_{\mathrm{deformation}}.$$

This derivative was also calculated numerically. The resulting forces by the actin cortex are shown in Figure S7 D. Depending on the size of the bead and its position along the cell, the forces are on the order of a few 10 pN to a few 100 pN. The forces increase with increasing diameter of the beads, however, they only scale linearly with D, and not with D^3 as measured in our experiments (Figure S7 E and F).

Modeling of the stall forces. We developed a theoretical model to explain the scaling behavior of the observed transport forces, taking the size-dependent displacement of microtubules by the phagosomes into account. This size-dependent displacement and bending was directly observed in confocal images of the microtubule cytoskeleton (Figures 4, S8, S16). The model assumes that the total number of microtubules that are displaced by a phagosome is proportional to the organelle's cross-sectional area:

$$N_{\mathrm{MT}} = \rho_{\mathrm{MT}}\, \pi \left(\frac{D}{2}\right)^2.$$

The cytoplasmic microtubule area density ρ_{MT} is the density of microtubules running through a cross-section of the cell orthogonal to its long axis. The model assumes that microtubules which are displaced by a phagosome bend around it, depending on their distance r to the phagosome center. They touch the phagosomes over an interaction angle $\theta_{\mathrm{int}}(r)$ (Figure S8 A). Microtubules with $r = 0$ are strongly displaced and bent by the phagosomes, so that they touch the phagosome over an angle $\theta_{\mathrm{int}}(r = 0) = \theta_0$. With increasing r, the microtubules are displaced and bent less by the phagosomes. Ultimately, microtubules with $r > \frac{D}{2}$ are not displaced or bent by the phagosomes and do not interact with their surface. This displacement and bending of microtubules was directly visible in the confocal images of the microtubules (Figure S8 B). We found experimentally that θ_0 was on average 108° ± 7° and independent of D. Therefore, we treated it as a constant in our model (Figure S16).
Overall, the interaction length $l_{\mathrm{int}}(r)$ on which a microtubule that would cross the phagosome at a given radius r will interact with it, is

$$l_{\mathrm{int}}(r) = \frac{D}{2}\theta_{\mathrm{int}},$$

with the interaction angle θ_{int}. As discussed above, the interaction angle decreases with increasing radius r:

$$\theta_{int} = \theta_0 \left(1 - \frac{2r}{D}\right)$$

Furthermore, only the projection of the force of a motor at a given angle θ along the interaction length of a microtubule contributes to the phagosomal stall force. We assume that motor force is directed along the microtubules, so that a motor at $\theta = \pm 90°$ will direct all its force perpendicular to the force applied by the magnetic tweezers and therefore does not contribute to the stall force. Similarly, we expect that for a motor at $\theta = 0°$, all its force will contribute to the phagosomal stall force (Figure S8 A). Therefore, we weighted the interaction length with $\cos(\theta)$ to obtain an effective interaction length of motors that contribute to the stall force:

$$l_{\text{int,eff}} = \frac{D}{2} \int_{-\frac{\theta_0}{2}(1-\frac{2r}{D})}^{\frac{\theta_0}{2}(1-\frac{2r}{D})} \cos(\theta)\, \mathrm{d}\theta = D \sin\left(\frac{\theta_0}{2}\left(1 - \frac{2r}{D}\right)\right).$$

Dyneins with a maximal distance to a microtubule of one motor length d_{M} may interact with this microtubule (Figure S8 A), leading to an effective area of interaction $A_{\text{int,eff}}$ of about $2\, l_{\text{int,eff}}\, d_{\text{M}}$ on the phagosome. Summing up all the areas of interaction for all the displaced microtubules by integrating over the cross-sectional area of the phagosome $A_{\emptyset}$ yields the effective number of all possible dynein-microtubule interactions that contribute to the stall force:

$$N_{\text{int,eff}}(D) = \rho_{\text{Dyn}}\, \rho_{\text{MT}} \int_{A_{\emptyset}} A_{\text{int,eff}}\, \mathrm{d}A = \rho_{\text{Dyn}}\, \rho_{\text{MT}}\, 2\, d_{\text{M}}\, D \int_0^{2\pi} \mathrm{d}\varphi \int_0^{D/2} \sin\left(\frac{\theta_0}{2}\left(1 - \frac{2r}{D}\right)\right) r\, \mathrm{d}r$$

The integral over r yields

$$\frac{D^2}{2\theta_0^2}\left(\theta_0 - 2\sin\left(\frac{\theta_0}{2}\right)\right) \approx 0.04\, D^2,$$

with $\theta_0 = 108°$ (Figure S16). The integral over φ simply yields 2π. Therefore, the effective number of possible dynein-microtubule interactions is:

$$N_{\text{int,eff}}(D) \approx 0.5\, \rho_{\text{Dyn}}\, \rho_{\text{MT}}\, d_{\text{M}}\, D^3$$

Together with the force of a single dynein F_{SD} and an offset c_{m} we modeled the measured stall forces:

$$F_{\text{stall}}(D) = F_{\text{SD}}\, N_{\text{int,eff}}(D) + c_{\text{m}} = f D^3 + c_{\text{m}},$$

with the force density $f = 0.5\, F_{\text{SD}}\, \rho_{\text{Dyn}}\, \rho_{\text{MT}}\, d_{\text{M}}$. This model fits the experimentally determined stall forces very well and provides a potential explanation for how the dynein numbers increasing with D^2 generate stall forces increasing with D^3.

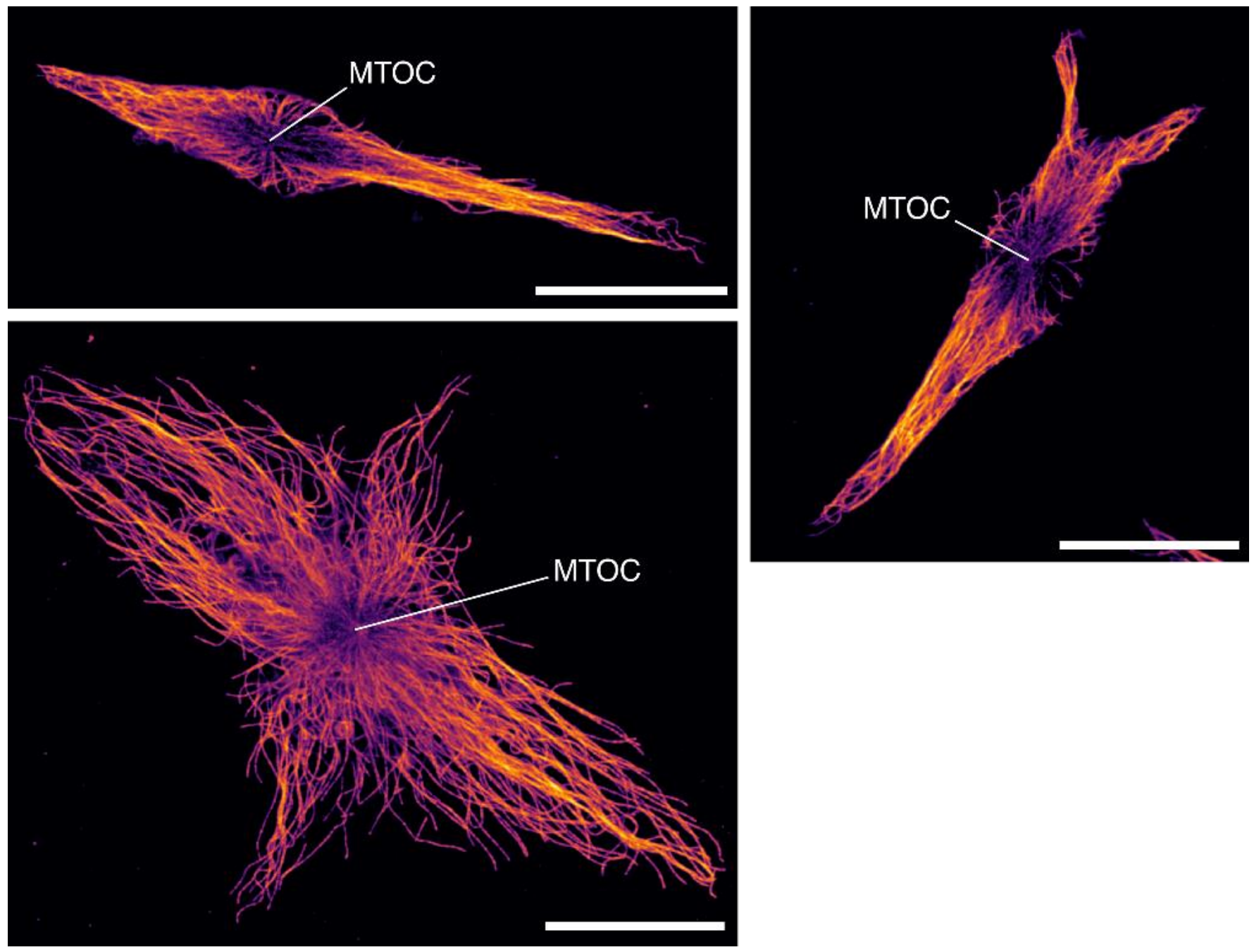

Figure S1: Confocal images of the microtubule skeleton of the J774A.1 cells. The images show that the microtubules originated from a common point, the microtubule organizing center (MTOC). In the cell's lamellipodia, the microtubules were oriented fairly parallel. Beaudet et al. (5) demonstrated that in J774A.1 macrophages, the microtubules are primarily oriented with their plus-ends out. In our transport measurements, the magnetic forces were directed along the long axis of the cells, and therefore acted parallel to the microtubules and opposite to the direction of motion of dynein. Scale bar: 20 μm.

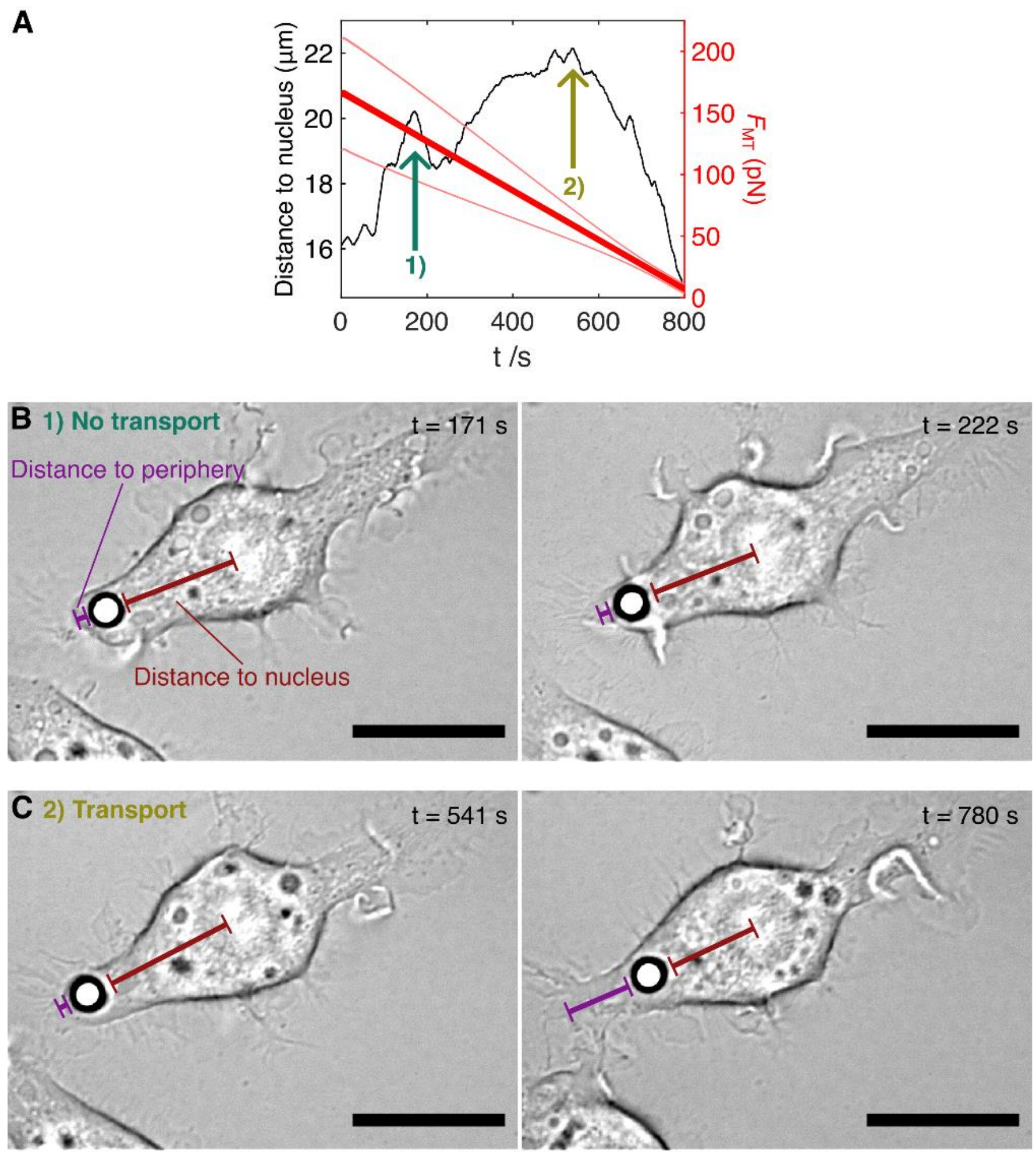

Figure S2: Example of the process to identify retrograde transport events. Here, a measurement of the retrograde transport force for a phagosome with a diameter of is shown. **A:** There are two possible transport events, marked 1) and 2) in the phagosome trajectory. In both cases, the phagosome moves persistently towards the nucleus for more than 1 µm. **B:** However, in the first case, the retrograde movement is caused by remodeling of the cell cortex and retraction of the cell's lamellipodium. During this movement, the phagosome's distance to the nucleus decreased, but its distance to the cell periphery did not change. Therefore, movements like this were not considered for the evaluation of the transport forces. **C:** In the second case, the distance to the nucleus decreased, and the distance to the periphery increased. Therefore, this movement was likely not related to dynamic changes of the cell cortex and was included in the evaluation of the transport forces. Scale bar: 20 µm.

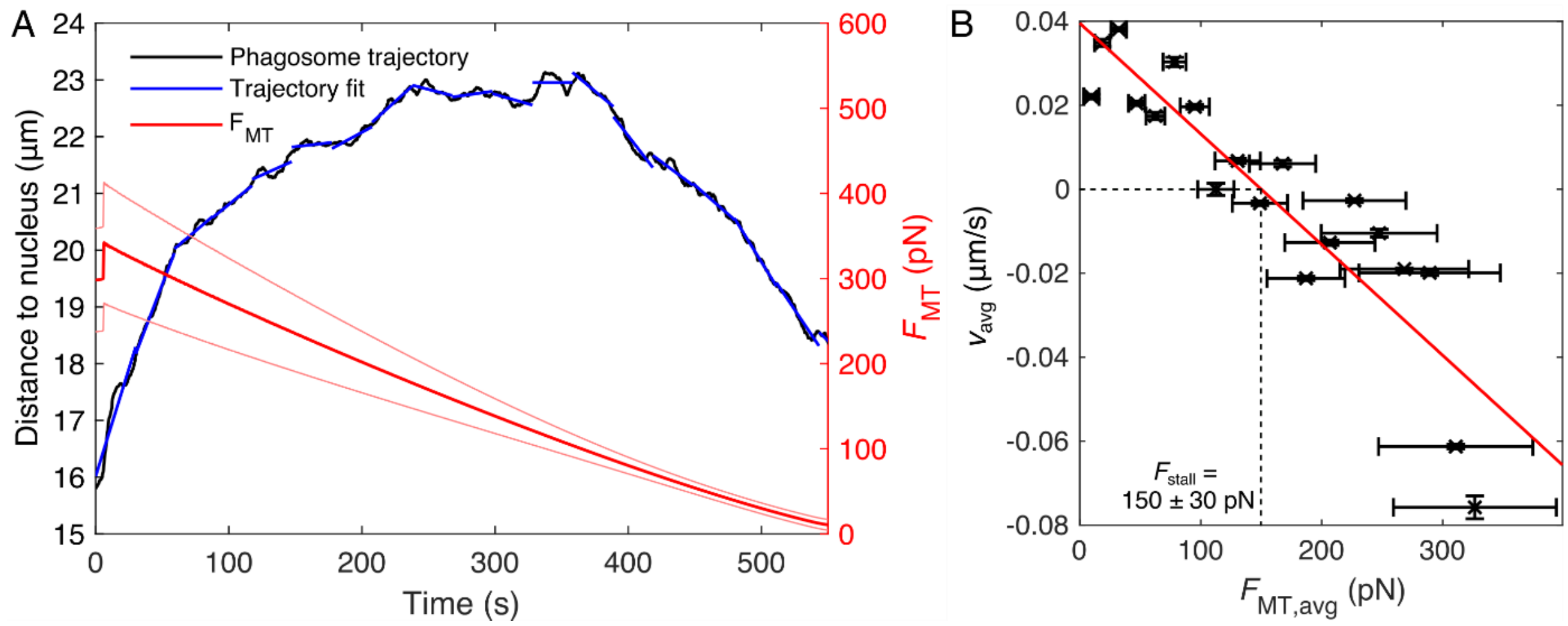

Figure S3: Analysis of the cytoplasmic drag. Stokes' law of friction was fitted to $v_{\mathrm{avg}}(F_{\mathrm{MT,avg}})$. Here, the evaluation procedure for an example measurement with a 5 µm phagosome is shown. **A:** v_{avg} was estimated by fitting a piecewise linear function to the bead trajectories in intervals of 30 s. $F_{\mathrm{MT,avg}}$ was calculated as the arithmetic mean of the magnetic force on the particles in the corresponding intervals. **B**: The data obtained in this way were then fitted with SI equation 1. For this measurement, we observed an effective cytoplasmic viscosity of (80 ± 10) Pa s and a stall force of (150 ± 30) pN.

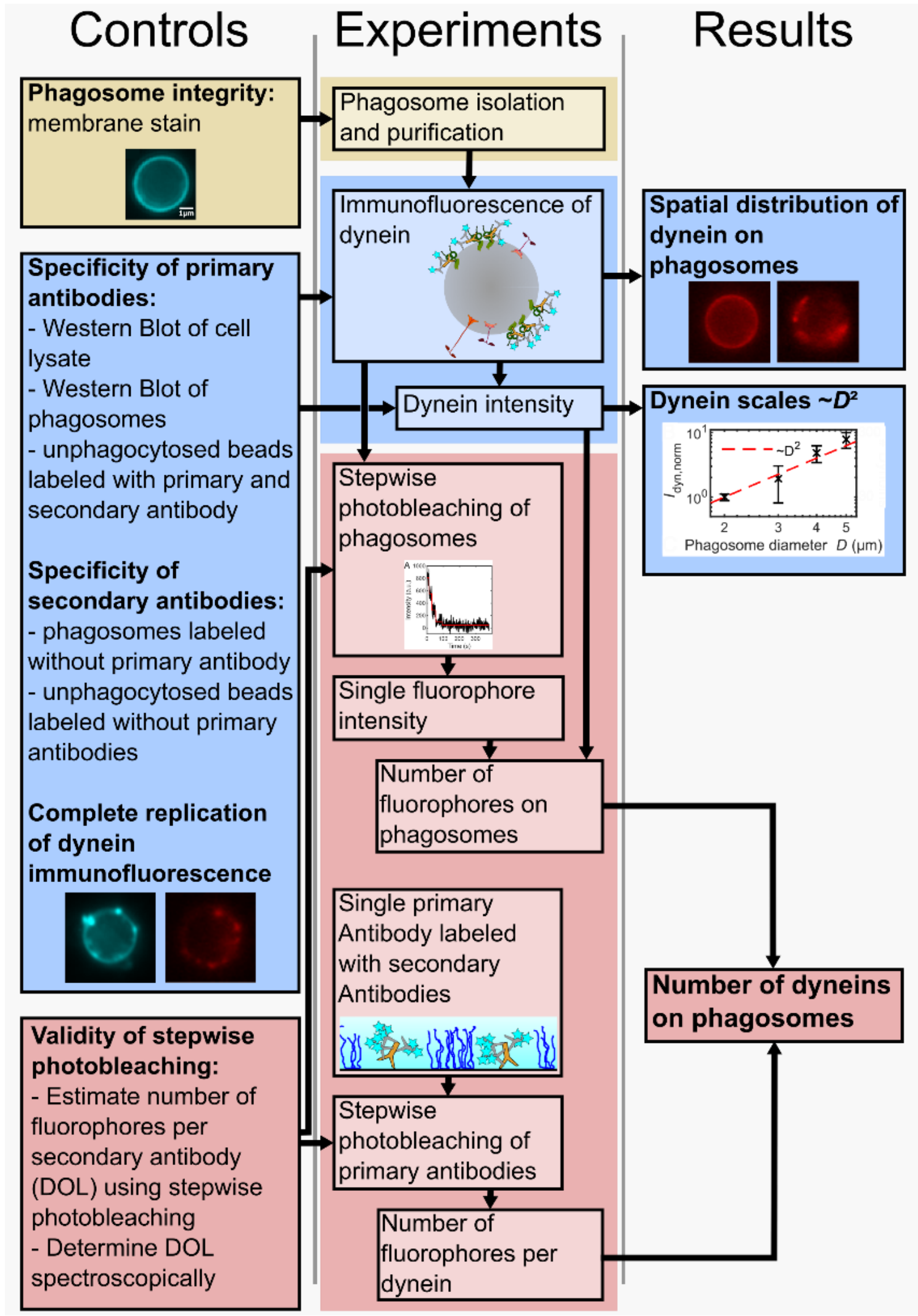

Figure S4: Overview of experiments and corresponding controls. To visualize and quantify dynein on phagosomes we followed a three-step procedure: First, phagosomes were isolated from the cells (color-coded in yellow), then dynein on the isolated phagosomes was labeled via immunofluorescence (color-coded in blue), and lastly dynein was quantified using stepwise photobleaching (color-coded in red). For each step, we performed extensive control experiments.

Briefly, we isolated the phagosomes by homogenizing cells with internalized beads in a homogenization buffer containing EGTA, protease inhibitors, ATP, and DTT. To ensure that the isolated phagosomes were intact, we stained the phagosomal membrane using the fluorescent membrane dye FM4-64.

In the next step, we immunofluorescently labeled dynein on the isolated phagosomes. Briefly, we let the isolated phagosomes sediment on silanized coverslips, which were then blocked using Pluronic F-127. We incubated them with the primary antibodies overnight at 4°C. After thoroughly washing them 2x with buffer containing 0.1% Tween-20 and 1x with buffer only, the phagosomes were incubated with the secondary antibodies for 45 minutes at room temperature. Again, they were washed 2x with buffer containing 0.1% Tween-20 and 1x with buffer only. In the images of the phagosomes, we observed that dynein was not homogeneously distributed over their surface, indicating that dyneins cluster into teams. Furthermore, we measured the total intensity of the dynein signal, which increased with the phagosome surface ($\sim D^2$), indicating that the dynein density on the phagosomes does not depend on phagosome size. To ensure that these conclusions drawn from the immunofluorescence assay are valid we performed extensive controls. We tested the

specificity of the monoclonal primary antibody with Western Blots of total cell lysate and the isolated phagosomes. Furthermore, we labeled unphagocytosed beads with primary and secondary antibodies to ensure that no unspecific binding to the beads occurred. In addition, we tested the specificity of the secondary antibody by labeling both phagosomes and unphagocytosed beads with the secondary antibody only. Lastly, we replicated the immunofluorescence assay of dynein with new phagosomes and different types of primary and secondary antibodies.

To quantify the number of dyneins on the phagosomes from their fluorescence intensity, we performed stepwise photobleaching on the phagosomes. This technique allows us to estimate the intensity of single fluorophores in the sample. To ensure a good resolution of the individual steps, we focused on “dim” trajectories that only had about 5-15 steps. Knowing the intensity of a single fluorophore, we extrapolated the number of fluorophores attached to the phagosomes. To determine the number of dyneins, we additionally needed to calibrate the number of fluorophores per dynein. We therefore incubated silanized coverslips in a solution of diluted primary antibodies, so that individual antibodies could attach to the coverslip. After blocking the coverslip with Pluronic F-127, we labeled the primary antibodies with the secondary antibodies as described above. Performing stepwise photobleaching on these samples yielded the number of fluorophores attached to a primary-secondary antibody complex. Since we used a monoclonal antibody with a known epitope on the dynein intermediate chain, we calculated the number of fluorophores attached to a single dynein. Combining the number of fluorophores on the phagosomes with the number of fluorophores per single dynein allowed us to determine the number of dyneins per phagosome. We validated this technique by estimating the degree of labeling (DOL) of the secondary antibody using stepwise photobleaching. We then compared this value to the DOL measured with a well-established spectroscopical method (Table S3). Both values agreed within a 10% margin, underlining the validity of the stepwise photobleaching.

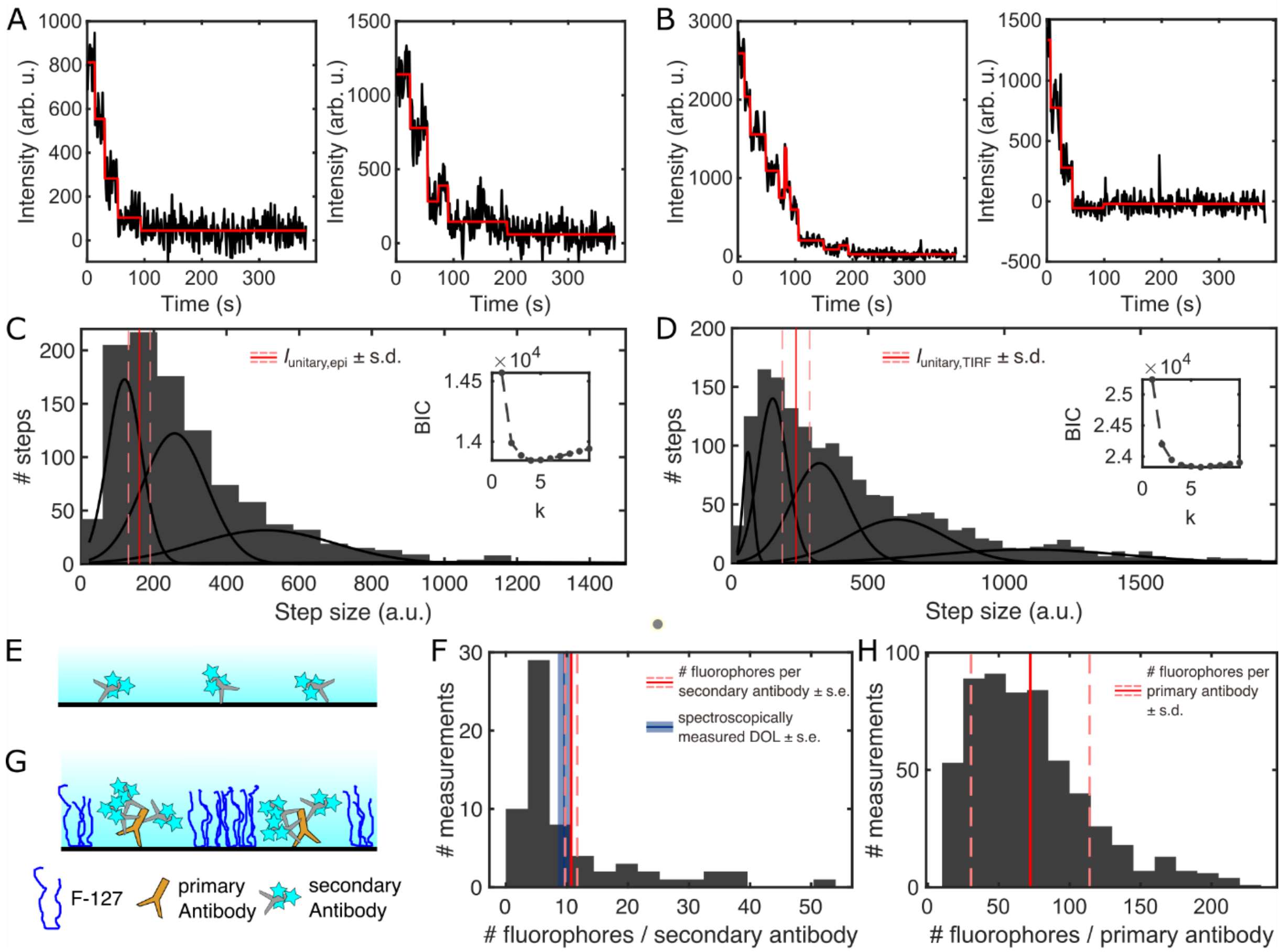

Figure S5: Quantification of dynein numbers by stepwise photobleaching. A: Examples of photobleaching curves measured in the epifluorescence mode of the microscope. Discrete steps in the signal correspond to one or multiple fluorophores bleaching. The signal was fitted with a piecewise constant function using an algorithm based on a two-sided t-test. More details about the acquisition of the photobleaching curves are provided in Figure S6. **B:** Examples of photobleaching curves measured in the TIRF mode of the microscope, fitted with a piecewise constant function. **C:** The distribution of step sizes was fitted with a Gaussian mixture model to estimate the unitary step size in the epifluorescence mode, which corresponds to the intensity of a single fluorophore. The number of components for the Gaussian mixture was determined by evaluating the Bayesian information criterion (small inset). We found a unitary step size of (160 ± 30) arb. u. in the epifluorescence mode. **D:** In TIRF mode, we analogously estimated a unitary step size of (235 ± 50) arb. units. **E:** To estimate the numbers of fluorophores bound to a single secondary antibody, we let diluted secondary antibodies attach to the coverslip and evaluated their intensity in TIRF mode. **F:** Using the unitary step size in TIRF mode, we found that on average 11 ± 1 fluorophores are bound to a single secondary antibody. This agreed very well with the independently measured spectroscopic degree of labeling (DOL) of 10 ± 1 fluorophores per secondary antibody. The variation of the number of fluorophores per secondary antibody likely originated from the dye's chemical coupling process, binding covalently to lysine residues on the IgG. With roughly up to 90 lysine residues on a single antibody, larger numbers of fluorophores per antibody are in principle possible (6, 7). **G:** Similarly, we determined the number of fluorophores bound to a single primary-secondary antibody complex. Therefore, we let diluted primary antibodies attach to the coverslip, blocked the coverslip with F-127, and labelled the primary antibodies with the secondary antibodies. **H:** We observed that on average 70 ± 40 fluorophore are present in a single primary-secondary

antibody complex. Since each dynein complex carries two dynein intermediate chains, we expect two primary antibodies and therefore about 140 fluorophores per dynein complex.

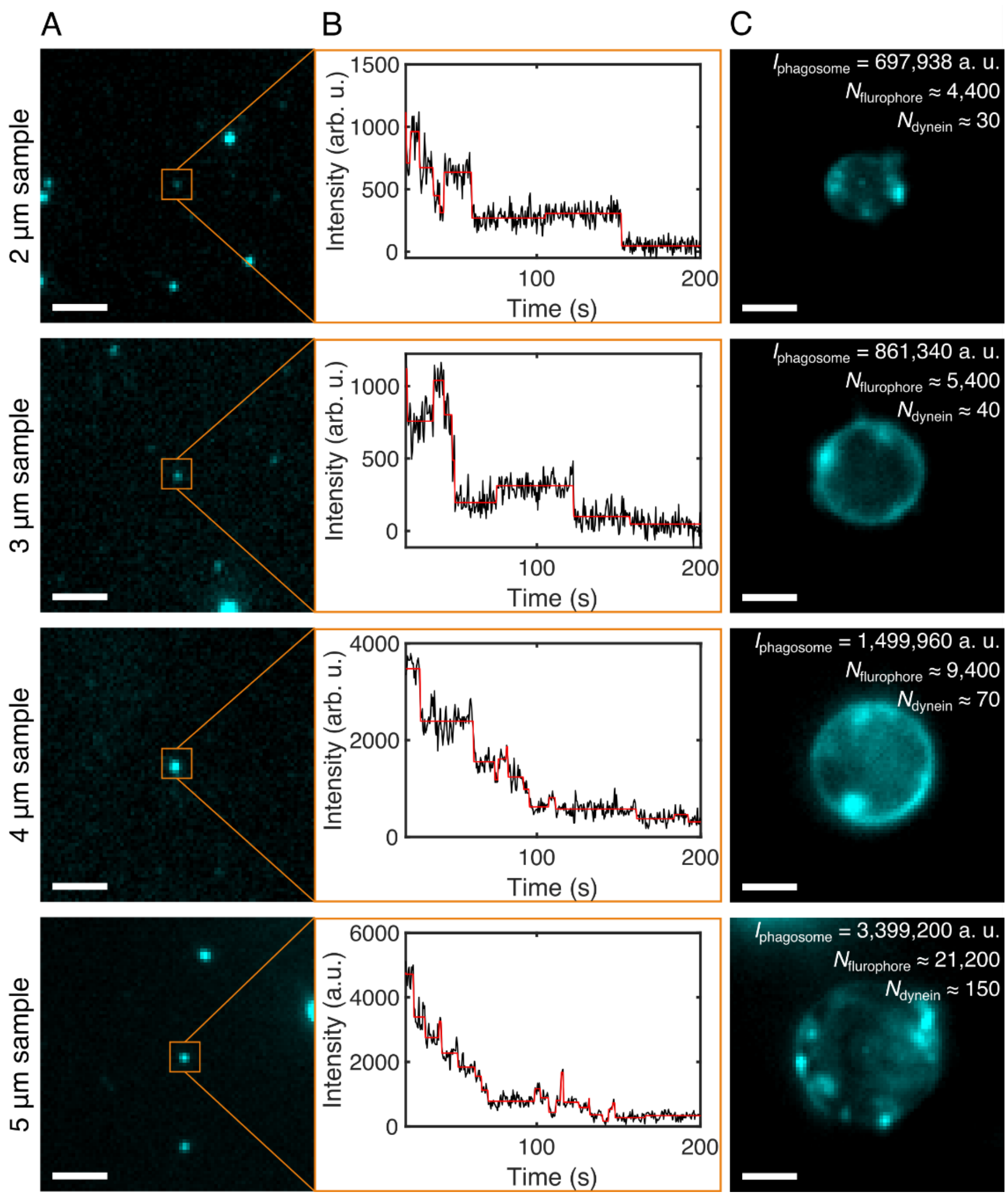

Figure S6: Detailed examples of photobleaching curves. A: In the phagosome samples with immunofluorescently labeled dynein, photobleaching curves of small fluorescent spots on the coverslips were evaluated. These spots might have originated from unspecific binding of the antibody to the coverslips or binding to remnants of cell debris in the phagosome samples, which adhered to the coverslips in small amounts. On average, the intensity of these fluorescent spots was 13× the unitary step size. In larger objects, like complete phagosomes, resolving individual steps was challenging due to the signal variance exceeding the single fluorophore intensity. **B:** The resulting photobleaching curves were fitted with a piecewise constant function. To avoid overfitting, only downward steps > 70 arb. u. were considered. Each step corresponds to the intensity decrease for one or multiple bleaching fluorophores. The photobleaching curves from all samples were evaluated together to determine the intensity of a single fluorophore $I_{\mathrm{unitary}}^{\mathrm{epi}}$ = 160 ± 30 arb. u. **C:** Examples of phagosomes with immunofluorescently labeled dynein. The fluorescent spots shown in **A** and the phagosomes shown in **C** are from the same sample, respectively. Using $I_{\mathrm{unitary}}^{\mathrm{epi}}$ we calculated the number of fluorophores $N_{\mathrm{Fluorophore}}$ per phagosome. With the number of

fluorophores per dynein (Figure S5), we then determined the number of dyneins N_{dynein} per phagosome. Scale bars: 2 μm.

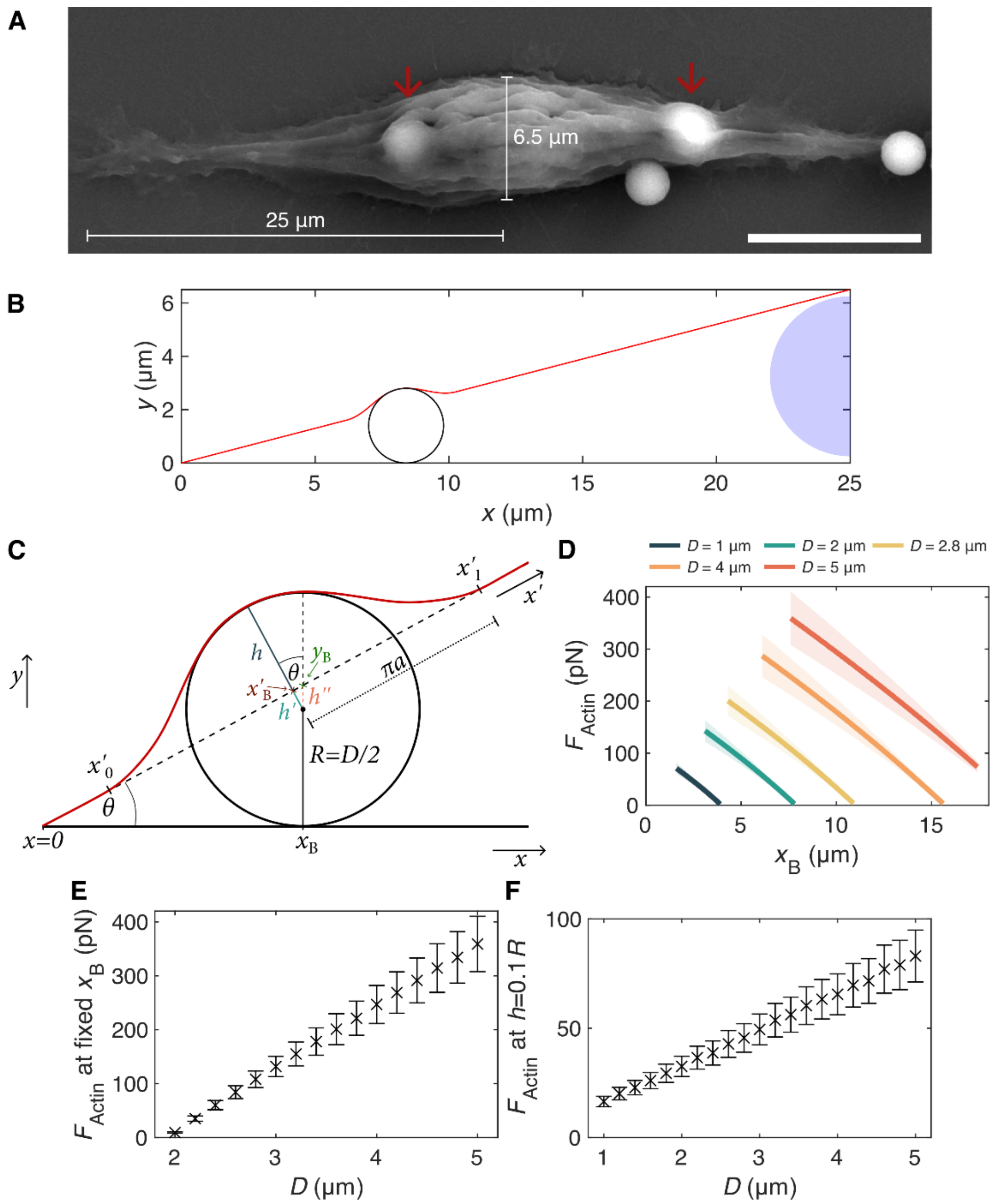

Figure S7: Model of the elastic forces by the cell cortex. A: Scanning electron micrograph of a J774A.1 cell which internalized two 2.8 µm beads (highlighted with red arrows). Depending on their position, the beads slightly deformed the cell cortex. **B:** To model this deformation, we assumed that the cell has a wedge-like shape, and the deformation can be described heuristically by a cosine, which smoothly transitions into the undeformed cell at its edges and has a radius of curvature that is equal to the bead radius at its center. **C:** Sketch of the geometry of the deformation. **D:** Resulting forces of the elastic models. Forces are on the order of 10-400 pN and depend on the phagosomes' size and position along the cell. **E:** At a fixed bead position x_B of 7.4 µm, the elastic forces due to the actin cortex increase linearly with phagosome size. **F:** Likewise, the elastic forces increase linearly with phagosome size for a fixed deformation of $2h/D$ = 0.1.

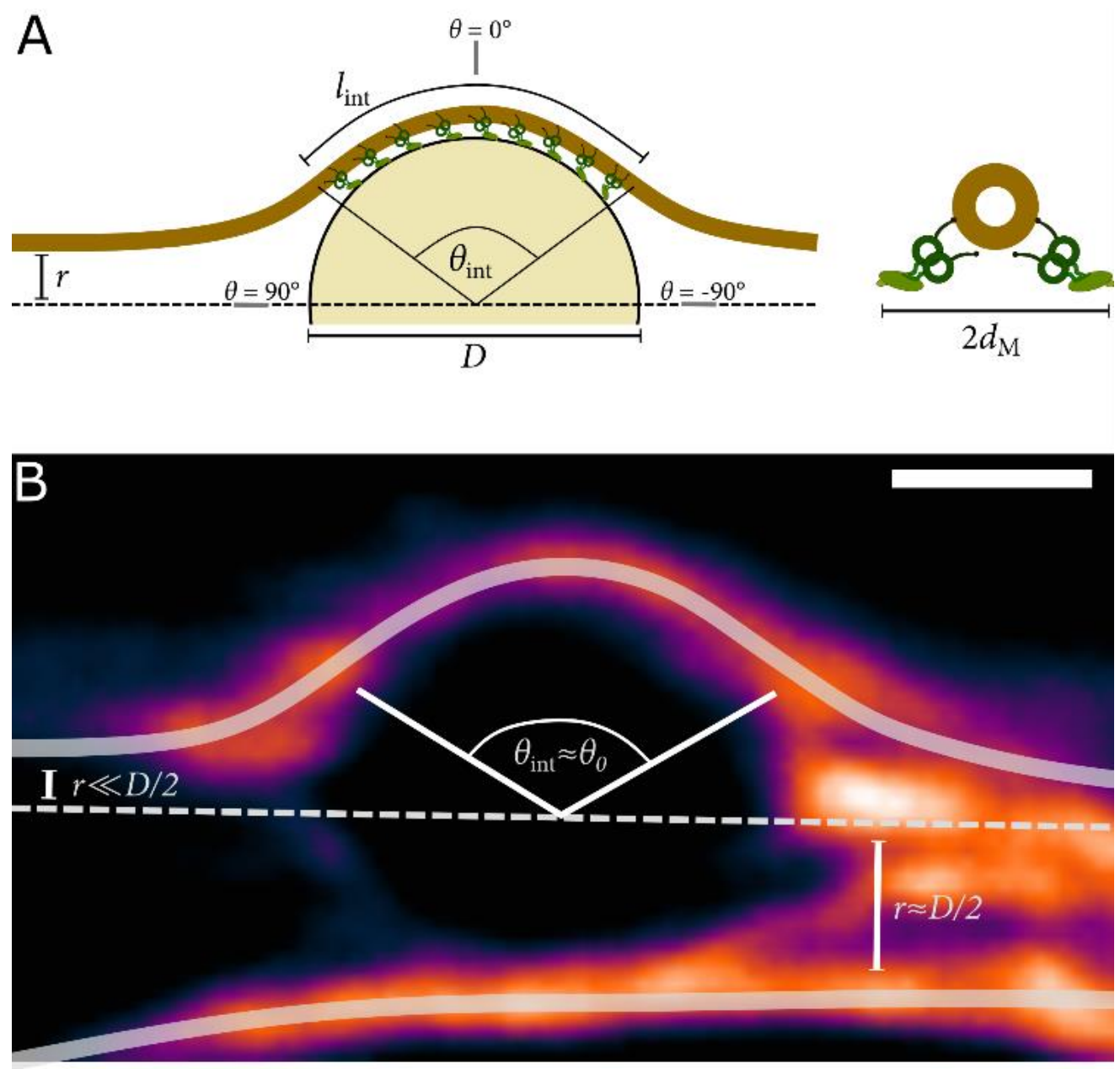

Figure S8: Geometry of microtubule bending. A: Microtubules bend around organelles depending on their distance r to the phagosome center. Microtubules that would run close to the phagosome center $r \approx 0$ are strongly displaced and bent by the phagosome. These microtubules touch the phagosomes over an angle of $\theta_{\text{int}}(r \approx 0) \approx \theta_0$. Motors that are closer than d_{M} to this microtubule may interact with it, leading to an interaction area A_{int} on the phagosome in which motors can interact with this microtubule. **B:** The displacement and bending of microtubules by phagosomes was directly visible in the confocal microtubule images. Here, a microtubule with $r \ll \frac{D}{2}$ is strongly displaced and bent around the phagosome, another microtubule with $r \approx \frac{D}{2}$ barely is affected. The confocal image shows a phagosome in a fixed cell which was not subjected to an external magnetic force. Scale bar: 1 µm.

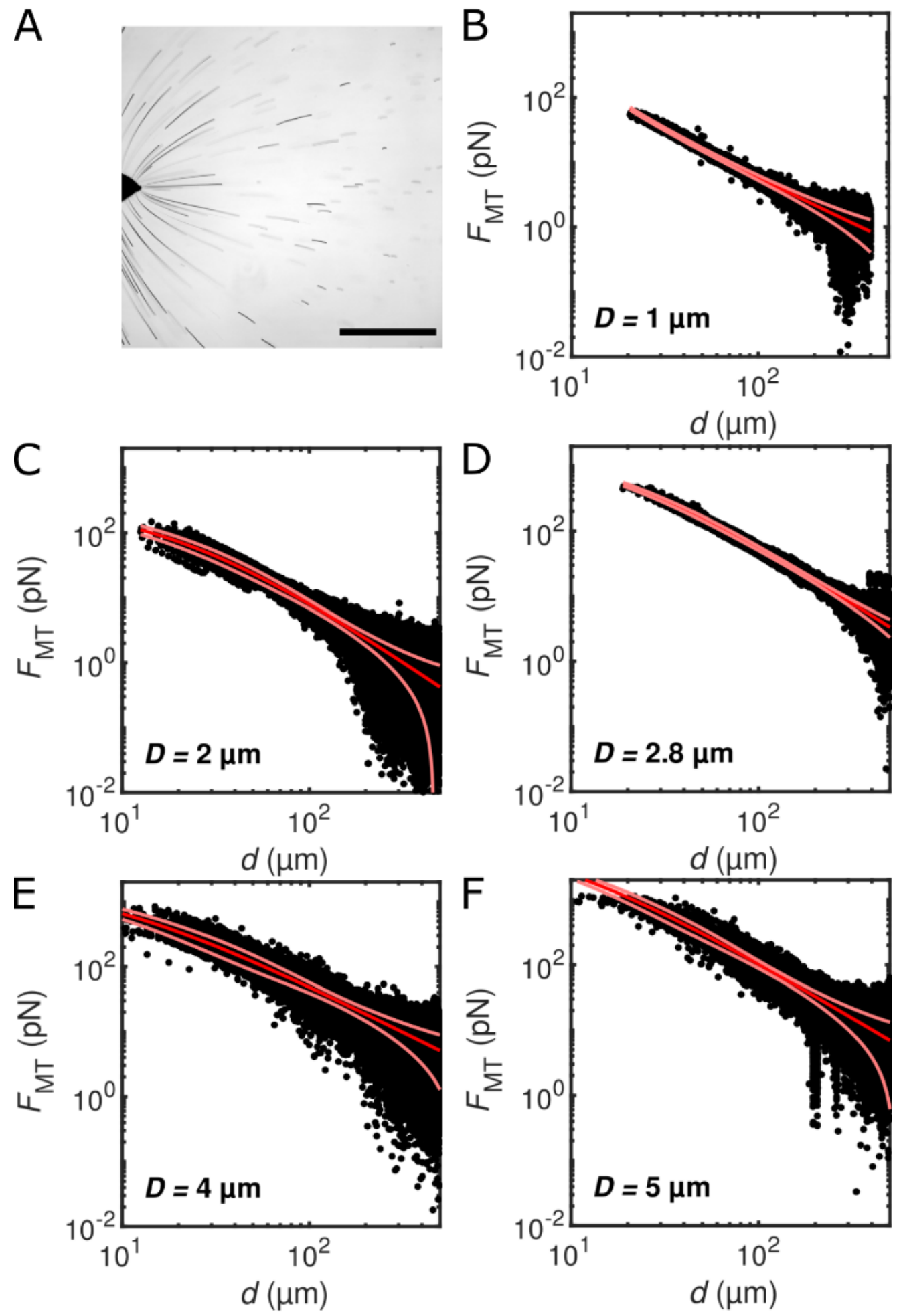

Figure S9: Calibration of magnetic tweezers. We analyzed the motion of the magnetic particles towards the tip of the magnetic tweezers in a viscous glycerol-water solution. **A:** Minimal intensity projection of a calibration measurement. Scale bar: 200 µm. The magnetic beads move along the magnetic field gradient with a velocity v that depends on the tip-bead distance d. Using Stokes' law of friction, we evaluated the force-distance relationship $F_{\mathrm{MT}}(d)$ for each bead type (**B-F**). The resulting calibration curves were heuristically fitted with a power law $F_{\mathrm{MT}}(d) = F_0 / \left(\frac{d-r_0}{d_0}\right)^c$, shown in red. The uncertainty of the calibration curves (shown in light red) was determined from the scattering of the data points, containing tracking errors and variations in size and magnetic moment of individual beads, and the uncertainty of the viscosity of the calibration. Sample size $N =$ 77,277 (1 µm phagosomes), 501,545 (2 µm), 401,077 (2.8 µm), 389,425 (4 µm), 414,788 (5 µm).

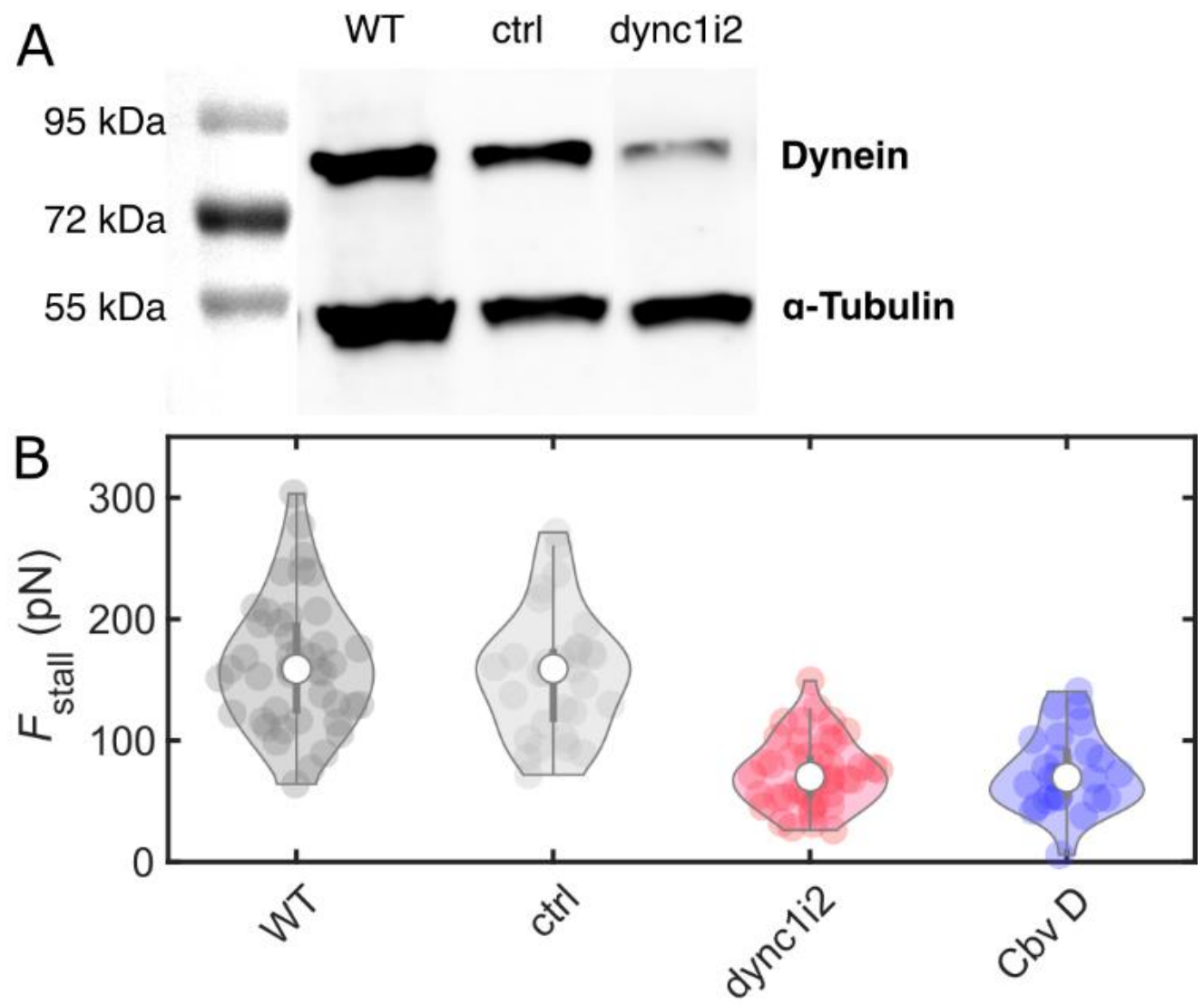

Figure S10: Dynein knockdown and inhibition experiments. To investigate the role of dynein in the transport of phagosomes, we treated cells with siRNA targeted to dync1i2 and the small-molecule dynein inhibitor ciliobrevin D, respectively. **A:** The Western blot indicates that the expression level of the dynein intermediate chain dync1i2 was reduced to (50 ± 10) % compared to the negative control treatment and the wild-type cells. **B:** Transport forces of phagosomes with a diameter of 5 μm for cells treated with siRNA targeted to dync1i2 and 50 μM ciliobrevin D (Cbv D). The forces in the case of both treatments were reduced to about 50% of the value of the negative control and the wild-type cells. This is consistent with a reduction of the expression level of dynein to about 50% in the siRNA-treated cells and the reduction of its ATPase activity to about 50% in the ciliobrevin-treated cells, respectively. Our results indicate that in this case mainly dynein is driving the centripetal transport of the organelles. Sample size $N = 44$ (WT), 31 (ctrl), 42 (dync1i2), 25 (CbvD).

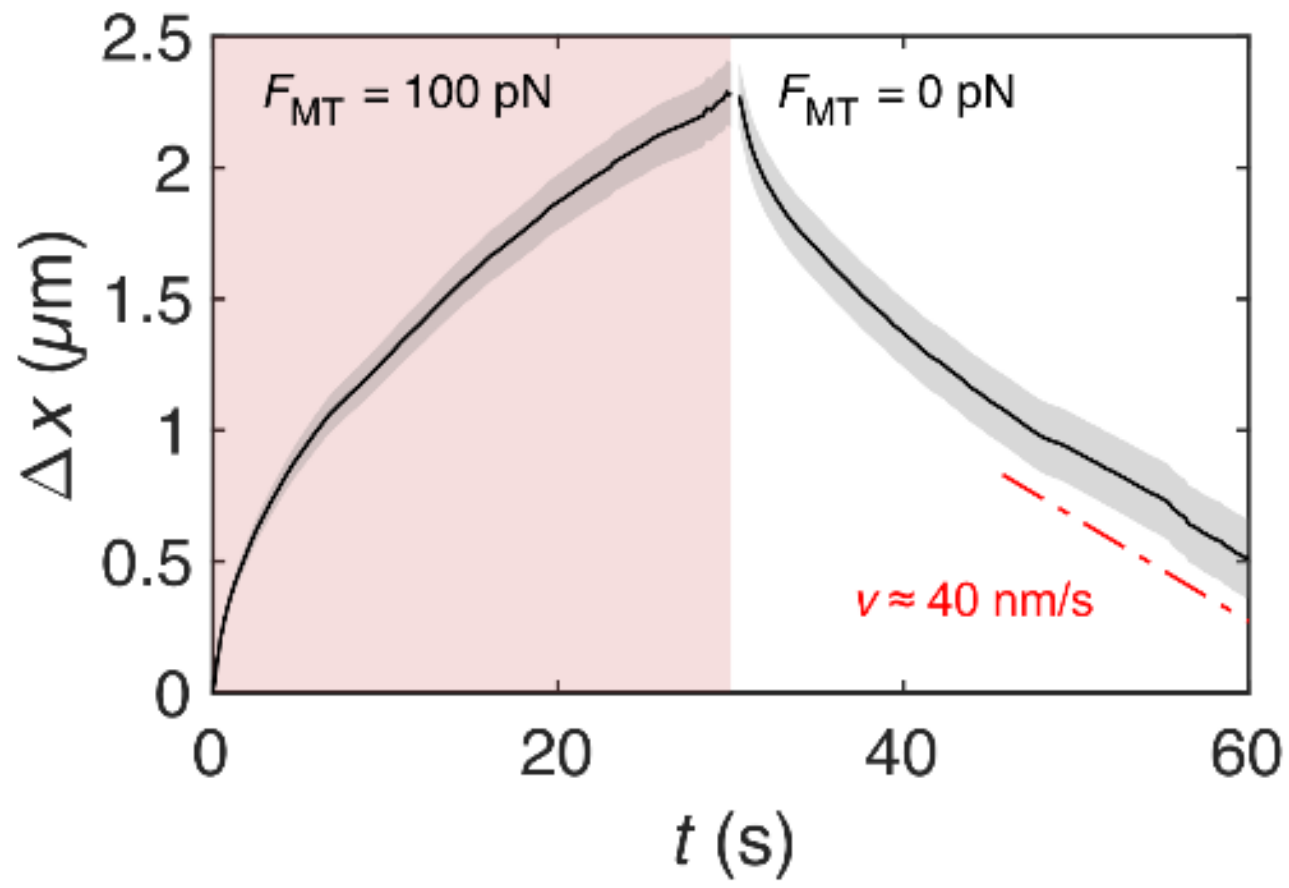

Figure S11: Measurement of cell rheology. To measure the rheological properties of the cells, we applied a high alternating magnetic force of 100 pN to the 2.8 μm phagosomes. The force was periodically switched on and off with a period of 60 s. Here, the mean displacement Δx of the phagosomes from their initial position over one switching cycle is shown. The magnetic force F_{MT} was turned on at *t* = 0 s and kept constant at 100 pN (red shaded area) until it was switched off again at *t* = 30 s. When the magnetic force was on, the particle was displaced from its original position. This displacement followed a power law, as described previously (9, 10). Once the magnetic force was turned off, the phagosome started to relax back towards its original position. However, the phagosomes did not fully reach their initial position, which indicates that there was an inelastic deformation of the cell cortex due to its viscoelastic properties. Interestingly, the creep function seemed to transition from a power law to a linear function 15-20 s after the magnetic force was turned off. Then, the phagosome moved with a constant velocity of approximately 40 nm s^{-1}, which is on the same order of magnitude as the free transport velocity observed during retrograde transport (Figure 2 F). To further analyze this behavior, we fitted a power law of the form $\Delta x(t) = x_0 t^{\varepsilon}$. In the context of power-law rheology, the scaling exponent ε of this power law shows whether the environment of the phagosome behaves like an elastic solid ($\varepsilon = 0$) or viscous fluid ($\varepsilon = 1$) (9, 10). Since cells are viscoelastic and have both elastic and viscous components, $0 < \varepsilon < 1$ should be expected (9). Consistently, we observe for the creep phase (*t* = 0-30 s) an exponent $\varepsilon =$ 0.521 ± 0.004. Interestingly however, the recovery phase can be divided into two parts: In the first half of the recovery phase (*t* = 30-45 s), the viscoelastic exponent is very similar to the creep phase, with $\varepsilon =$ 0.578 ± 0.002. However, in the second half (*t* = 45-60 s), the recovery has transitioned to a purely viscous-like motion, with a viscoelastic exponent of $\varepsilon =$ 1.02 ± 0.02. This result indicates that elastic forces no longer play a role, and the phagosome moves with a constant velocity, consistent with free transport by dyneins.

In total, we analyzed 97 phagosome trajectories in the creep phase and 86 phagosome trajectories in the recovery phase, originating from 14 individual cells. Shaded areas of uncertainty represent the standard error of the mean.

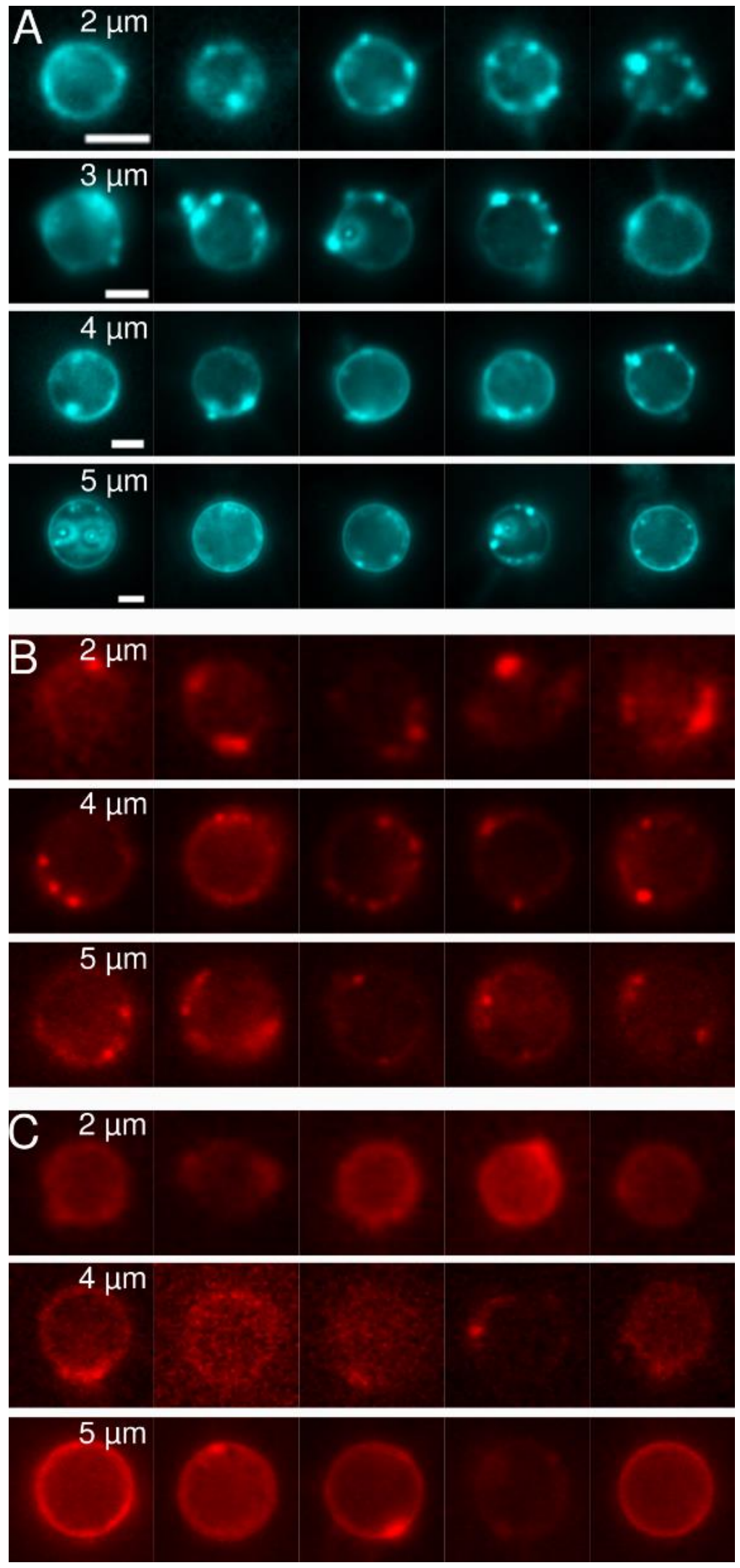

Figure S12: Replication of dynein immunofluorescence. To verify our immunofluorescence assay, we replicated the labeling of dynein with new phagosomes and different types of antibodies. The new phagosomes were isolated from the cells following the same procedure as before. However, instead of homogenizing the cells with a syringe we now used a dounce homogenizer. The original antibodies were Invitrogen Dynein Monoclonal Antibody (74.1), Thermo Fisher Scientific, Catalogue Number MA1-070 (primary) and Invitrogen Goat Anti-Mouse IgG, DyLight 488 conjugated, Thermo Fisher Scientific (secondary). For the replication, we used Dynein HC Antibody, Santa Cruz, Catalogue Number sc-9115 (primary), and Goat Anti-Rabbit IgG – Atto 488, Sigma Aldrich, Catalogue Number 18772 (secondary). The results obtained from the original and new immunofluorescence assay of dynein were remarkably similar. **A:** Examples of the original dynein labelling assay. Dynein localized predominantly to discrete spots, indicating clustering of dynein into teams. **B:** The same localization pattern was observed in the replicated dynein assay. **C:** We also labeled kinesin-2 on the new phagosomes, which was previously reported to be present on phagosomes (3, 11). Unlike dynein, kinesin was distributed homogeneously over the phagosomes' surface, indicating that the localization pattern observed for dynein was indeed not an artifact.

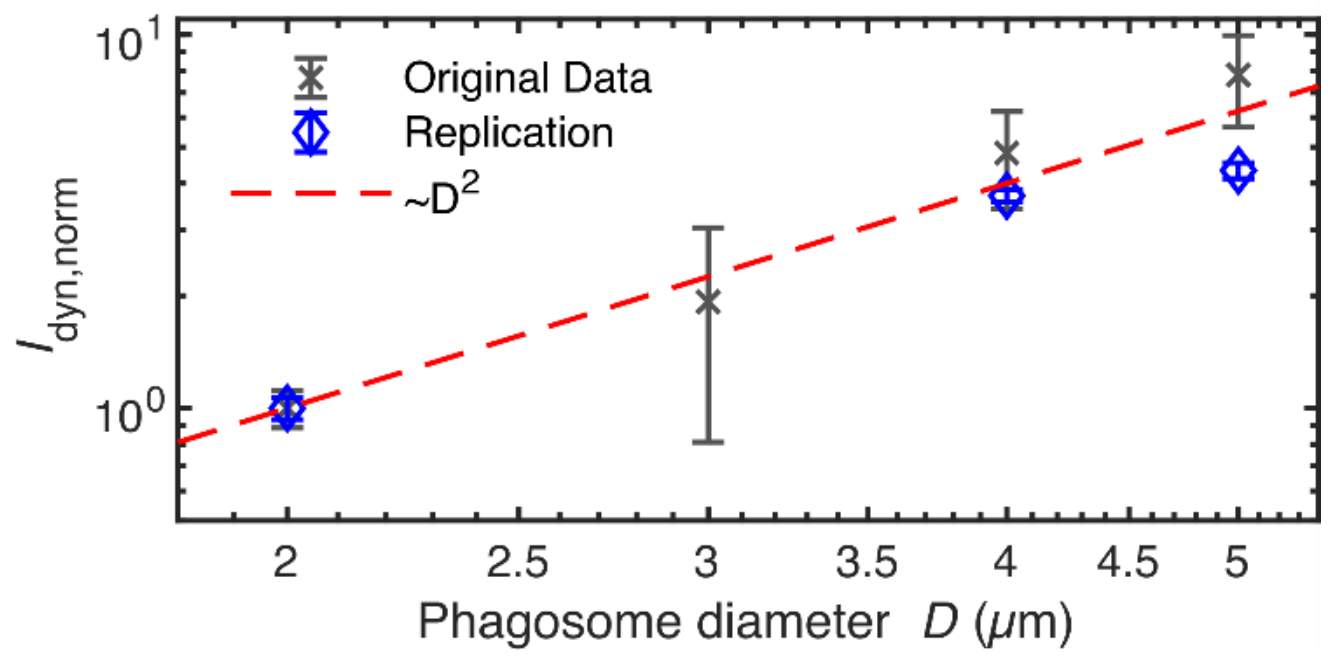

Figure S13: Comparison of original and replicated dynein intensities. We additionally determined the intensity of dynein for phagosomes of different size in the replicated immunofluorescence assay. In the replicated assay, we used a different batch of isolated phagosomes, different primary and secondary antibodies, different fluorophores, and a different microscopy setup. Like in the original data, the dynein intensity did not scale with $\sim D^3$, but rather with $\sim D^2$. Therefore, both the original and replicated immunofluorescence assays are consistent with a constant dynein surface density on the phagosomes, independent of phagosome size. The amount of dynein present on the phagosomes alone is thus not sufficient to explain the scaling $\sim D^3$ of the transport forces.

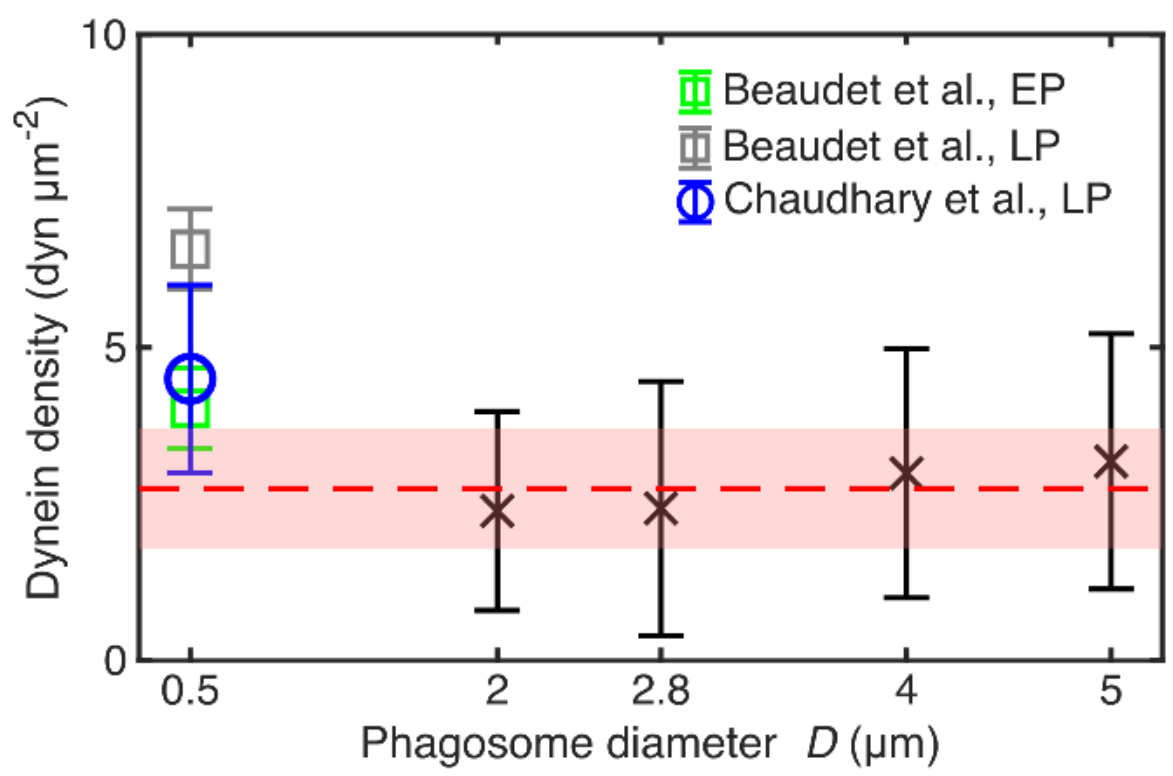

Figure S14: Comparison of the dynein surface density of phagosomes of different size with dynein densities published previously. Beaudet et al. (5) measured the dynein surface density on 0.5 µm phagosomes 30 min (early phagosomes, EP) and 90 min (late phagosomes, LP) after the particles were added to the cells. Chaudhary et al. (3) measured the dynein surface density of 0.5 µm phagosomes 90 min after the particles were added to the cells. The phagosomes used in this work were isolated 60 min after the beads were added to the cells. The dynein surface density of 3 ± 1 dyneins µm^{-2} we measured in this work were within the margins of uncertainty consistent with the dynein surface densities published earlier. The differences between our values and those of Beaudet et al. and Chaudhary et al. might be caused by the different duration of phagosomal maturation, slight differences in the treatment of the cells during phagosome isolation (e. g., homogenization with syringe instead of dounce homogenizer), and different procedures during purification and immunostaining of the phagosomes (e. g., magnetic separation of isolated phagosomes vs. ultracentrifugation). Overall, the agreement of the dynein surface density measured in this work and those published earlier underlines the validity of our procedures and reinforces our hypothesis that the dynein surface density is independent of phagosome size over a large range of diameters (0.5 µm – 5 µm).

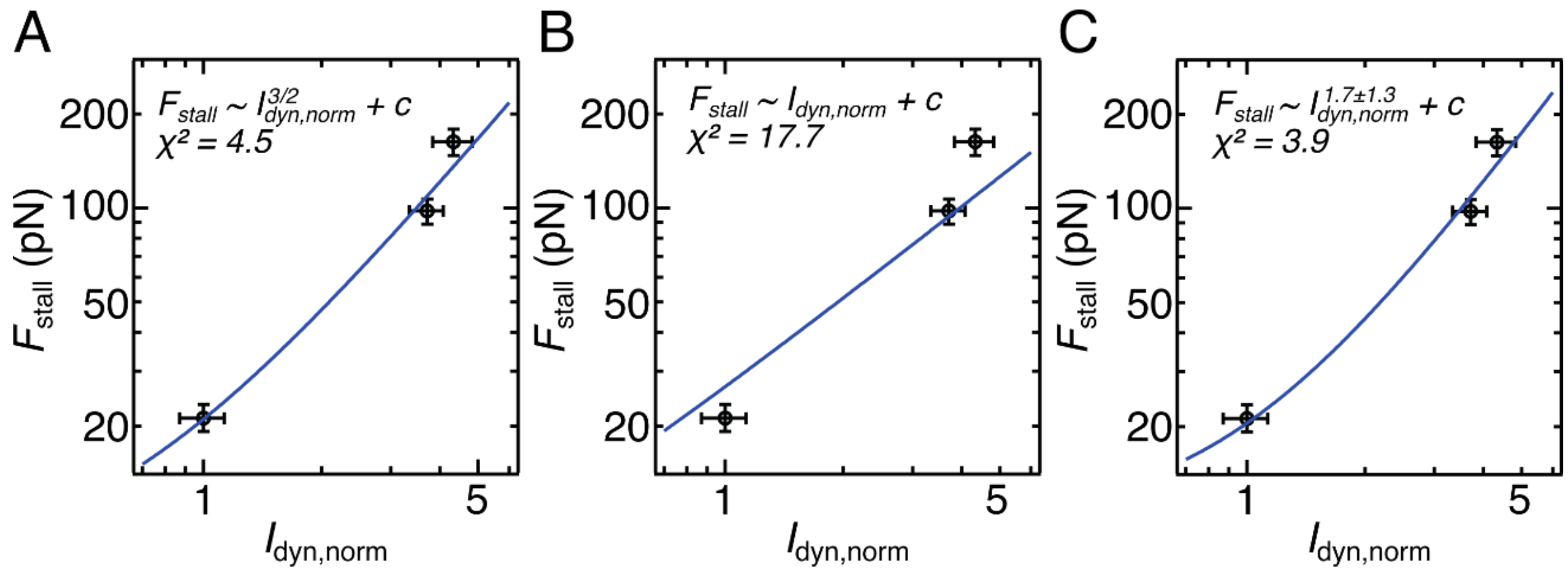

Figure S15: Stall forces as a function of the dynein fluorescence. To assess the force-motor scaling relationship, the stall phagosomal stall force F_{stall} was plotted against the corresponding normalized dynein intensity $I_{\text{dyn,norm}}$. They were fitted with $F_{\text{stall}} \sim I_{\text{dyn,norm}}^{3/2} + c$ **(A)**, $F_{\text{stall}} \sim I_{\text{dyn,norm}} + c$ **(B)**, and a free scaling exponent **(C)**. The constant offset c was included to acknowledge that due to our measurement method, at least one force producing unit was active during stall-force measurements. The χ^2 goodness-of-fit values suggest a better fit for $F_{\text{stall}} \sim I_{\text{dyn,norm}}^{3/2}$, which is consistent with the scaling exponent of 1.7 ± 1.3 for the free fit.

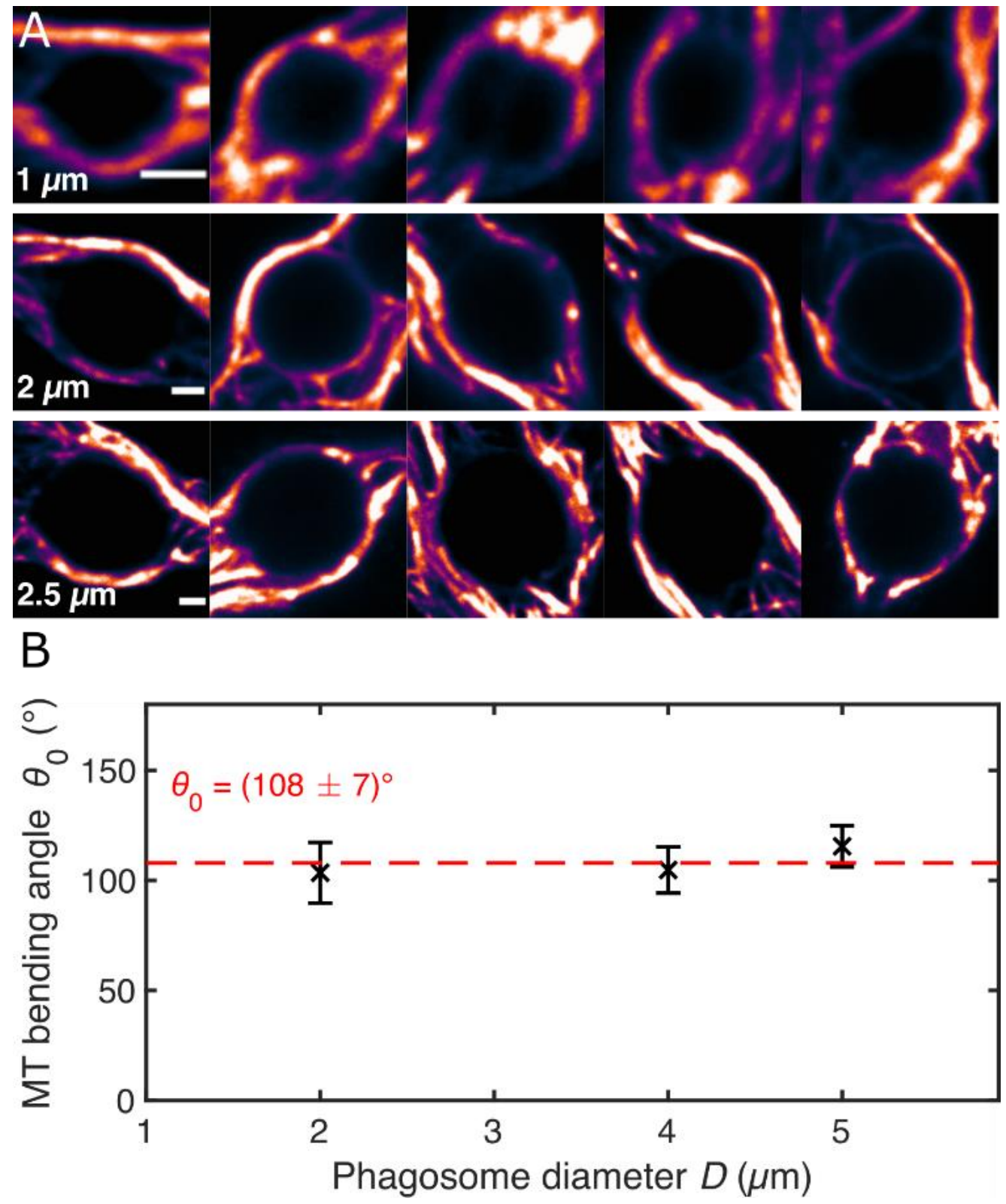

Figure S16: Analysis of microtubule binding angles. A: Examples of microtubules bending around phagosomes of different sizes. Scale bar: 1 µm. The confocal images show phagosomes in fixed cell which were not subjected to an external magnetic force. **B:** To investigate, whether the MT bending angle $\theta_{\mathbf{0}}$ depended on the organelle diameter D, we measured θ_0 using the angle tool of the image processing package Fiji. We always measured the interaction angle of microtubules that would run close to the microtubule center, $r \approx 0$ (compare Figure S8 B). We found that the microtubule bending angle was on average 108° ± 7° and that is was independent of the organelle radius. Therefore, we treated it as constant in our model. Sample size $N = 10$ (2 µm phagosomes), 14 (4 µm), 9 (5 µm). Error bars represent standard deviation.

Table S1: Resulting fit parameters from the cytoplasmic viscosity analysis. For the effective cytoplasmic drag η_{eff} see Table 1. The phagosome trajectories were analyzed using Stokes' law of friction: $v_{\text{avg}}(F_{\text{MT,avg}}) = \frac{1}{3\pi\, \eta_{\text{eff}}\, D}(F_{\text{stall}} - F_{\text{MT,avg}})$. The average free transport velocity corresponds to the phagosomes' average velocity without an external magnetic force: $v_0 = v_{\text{avg}}(F_{\text{MT,avg}} = 0)$.

D (µm)	F_{stall} (pN)	v_0 (nm s^{-1})
1	11 ± 10	23 ± 13
2	25 ± 10	20 ± 8
2.8	32 ± 15	24 ± 9
4	110 ± 40	25 ± 7
5	170 ± 40	37 ± 5

Table S2: Results for the fit parameters of the magnetic tweezers calibration. Results are shown for each particle size.

D (µm)	F_0 (pN)	r_0 (µm)	c	a (pN)	b (µm)	k (pN)
1	35	5.16	1.3466	1.35	15.61	0.4
2	180	-19.24	1.9962	24.8	27.22	0.5
2.8	929	-15.01	1.8582	44.1	34.47	1.0
4	399	-7.80	1.4488	154	29.31	3.8
5	1269	-7.33	1.7307	430	23.82	6.3

Table S3: Spectroscopic measurement of the degree of labeling (DOL) of the secondary antibody used to label dynein on isolated phagosomes. Measurements were performed in the storage buffer of the antibody (PBS @ pH 7.2 with 0.02% of sodium azide) and replicated 5 times. We the measured absorption at 280 nm (protein concentration) and 495 nm (dye concentration). Measurements were corrected for the absorption of the blank sample (PBS + 0.02% sodium azide). With the given spectroscopic properties of the DyLight 488 dye and IgG, we estimated that the degree of labeling of this antibody was 10 ± 1 fluorophores per antibody, which is in excellent agreement with the photobleaching measurements (11 ± 1 fluorophores per antibody). These measurements underline the validity of our stepwise photobleaching analysis.

	A_{280}	A_{495}	Degree of labeling
Individual replicates	0.838	0.254	9.52
	0.869	0.254	9.16
	0.860	0.289	10.6
	0.911	0.259	8.90
	0.845	0.254	9.43
**Average ± standard deviation**	**0.87 ± 0.03**	**0.26 ± 0.02**	**10 ± 1**

Table S4: Measured dynein intensities $I_{\mathbf{dyn}}$ on isolated phagosomes. Here, the dynein intensities measured on isolated phagosomes of the original assay and replication assay are provided in arb. u.

D (µm)	Original assay (95% CI)	Replication assay (95% CI)
2	6.9E+05 (5.5E+05 … 8.4E+05)	1.3E+06 (1.2E+06 … 1.5E+06)
3	1.3E+06 (0.2E+06 … 3.0E+06)	-
4	3.3E+06 (2.0E+06 … 4.8E+06)	5.0E+06 (4.5E+06 … 5.5E+06)
5	5.3E+06 (3.5E+06 … 7.3E+06)	5.8E+06 (5.1E+06 … 6.5E+06)

SI References

1. Keller, S., K. Berghoff, and H. Kress. 2017. Phagosomal transport depends strongly on phagosome size. *Sci. Rep.* 7:17068.
2. Crocker, J.C., and D.G. Grier. 1996. Methods of digital video microscopy for colloidal studies. *J. Colloid Interface Sci.* 179:298–310.
3. Chaudhary, A.R., F. Berger, C.L. Berger, and A.G. Hendricks. 2018. Tau directs intracellular trafficking by regulating the forces exerted by kinesin and dynein teams. *Traffic*. 19:111–121.
4. Chen, Y., N.C. Deffenbaugh, C.T. Anderson, and W.O. Hancock. 2014. Molecular counting by photobleaching in protein complexes with many subunits: best practices and application to the cellulose synthesis complex. *Mol. Biol. Cell*. 25:3630–3642.
5. Beaudet, D., C.L. Berger, and A.G. Hendricks. 2024. The types and numbers of kinesins and dyneins transporting endocytic cargoes modulate their motility and response to tau. *J. Biol. Chem.* 300:107323.
6. Holmes, K.L., and L.M. Lantz. 2001. Protein labeling with fluorescent probes. In: Methods in Cell Biology. . pp. 185–204.
7. Wu, K.-L., C. Yu, C. Lee, C. Zuo, Z.T. Ball, and H. Xiao. 2021. Precision Modification of Native Antibodies. *Bioconjug. Chem.* 32:1947–1959.
8. Lam, J., M. Herant, M. Dembo, and V. Heinrich. 2009. Baseline Mechanical Characterization of J774 Macrophages. *Biophys. J.* 96:248–254.
9. Berghoff, K., W. Gross, M. Eisentraut, and H. Kress. 2021. Using blinking optical tweezers to study cell rheology during initial cell-particle contact. *Biophys. J.* 120:3527–3537.
10. Kollmannsberger, P., and B. Fabry. 2011. Linear and nonlinear rheology of living cells. *Annu. Rev. Mater. Res.* 41.
11. Hendricks, A.G., E.L.F.F. Holzbaur, and Y.E. Goldman. 2012. Force measurements on cargoes in living cells reveal collective dynamics of microtubule motors. *Proc. Natl. Acad. Sci.* 109:18447–18452.
