# Supplementary material for: Many dynein teams collectively generate high forces during the transport of large organelles
Source: Biophys J. 2025 Sep 10;125(12):3009–24. doi: 10.1016/j.bpj.2025.09.012 (PMC13507173; doi:10.1016/j.bpj.2025.09.012)
Supplement: Supplementary file 2 [file mmc7.pdf]

Biophysical Journal
Article

Biophysical Society

Many dynein teams collectively generate high forces during the transport of large organelles

Simon Wieland,[1,2] Christina Steininger,[1] David E. Gitschier,[1] Marius M. Kaiser,[1] Wolfgang Gross,[1] Abdullah R. Chaudhary,[3] Jana Ritschar,[4] Christian Laforsch,[2] Adam G. Hendricks,[3] and Holger Kress[1,*]
[1]Biological Physics, University of Bayreuth, Bayreuth, Germany; [2]Animal Ecology I, University of Bayreuth, Bayreuth, Germany; [3]Department of Bioengineering, McGill University, Montreal, Quebec, Canada; and [4]Laboratory of Molecular Parasitology, University of Bayreuth, Bayreuth, Germany

ABSTRACT The transport of organelles is important to maintain cellular organization and function. Efficient retrograde transport of large organelles with a size of several micrometers requires high collective forces from multiple dynein motors. However, the exact transport forces and their dependence on the cargo size are unknown for large organelles. Furthermore, it is not known how many dynein motors are active during this transport and how they generate high collective forces sufficient to overcome the cytoplasmic drag. We measured forces generated during retrograde transport of phagosomes with diameters between 1 and 5 μm. Forces increased with phagosome volume and ranged from under 10 pN for the smallest up to 160 pN for the largest phagosomes. These forces matched the cytoplasmic drag to achieve equally fast transport with a velocity of 25 $\pm$ 4 nm s^{-1} for phagosomes of all sizes. To confirm the need for many dynein motors to generate such high forces, we labeled and quantified dynein on isolated phagosomes. We found up to 250 dyneins on the largest phagosomes and a dynein surface density that was independent of the phagosome size. We connected the dynein numbers and transport forces with a theoretical model of the microtubule distribution around the organelles. The model implies that, because larger organelles displace and bend the microtubules, disproportionately large numbers of dyneins can be active and contribute to the high transport forces of large phagosomes. Our results indicate that, during the transport of large organelles, many dyneins interact with multiple microtubules in a cargo-size-dependent manner to achieve sufficiently large transport forces.

SIGNIFICANCE Intracellular organelle transport is crucial for cell organization and function. Fast movement of large organelles from the cell periphery to the cell center requires many dynein motors. However, the exact transport forces and motor numbers for organelles with a diameter of several micrometers were unknown. We measured these parameters for organelles between 1 and 5 μm. Our findings show that transport forces surprisingly increase proportionally with organelle volume, with up to 250 motors on the largest organelles. We explained this scaling of forces by the distribution of microtubules around the cargoes. Microtubules, which act as tracks for the motors, bend around the organelles, enabling a disproportionately large number of motors to assist in the transport of very large organelles.

INTRODUCTION

Dynein-driven retrograde transport of organelles plays a key role in many cellular functions (1), such as the transport of endosomes and phagosomes (2,3), axonal transport (4), the nucleus (5), and the spatial organization of cells (6,7). Consequently, mutations in the dynein complex have been linked to a number of developmental, neuro-degenerative, and motor-neuron diseases (8,9). Due to its prominent role in retrograde organelle transport, many studies focused on investigating the mechanochemistry and biochemical regulation of dynein motility (1,10). In vitro force measurements on single mammalian dynein complexes indicate that dyneins produce forces between 1.1 pN (11,12) and 3–4 pN (13,14).

A reason for this large variability of measured forces is that purified cytoplasmic dynein is in an autoinhibited state that produces low forces and is incapable of processive runs (15,16). This weak processivity of single dynein complexes

Submitted February 14, 2025, and accepted for publication September 8, 2025.

*Correspondence: holger.kress@uni-bayreuth.de

Editor: Rudolf Merkel.

https://doi.org/10.1016/j.bpj.2025.09.012

© 2025 The Author(s). Published by Elsevier Inc. on behalf of Biophysical Society.
This is an open access article under the CC BY license (http://creativecommons.org/licenses/by/4.0/).

(17–19) can additionally lead to an underestimation of their force production in optical-tweezer measurements, because the stall force is not reached before the motors detach (20). However, when bound to cargo adaptors such as dynactin or BICD2, dynein switches from an autoinhibited state to a state that is much more processive and capable of producing higher forces (13,14).

Dynein's processivity drastically increases in vitro when two or more dynein complexes are active, indicating that individual motors can work together to drive persistent transport (15,18,19,21,22). Moreover, the forces generated collectively by multiple dyneins are additive, enabling robust transport of cargoes in vitro (19,23). This ability to work together is likely caused by adaptor proteins that recruit multiple dyneins (22) and dynein's large and flexible structure, allowing dynein to adapt its stepping in a load-dependent manner (23). For example, under a resisting load, reduced step sizes and increased backstepping of dynein have been observed, which enable dynein to function as a "gear mechanism" (12,23). Furthermore, a low unbinding rate from microtubules allows dynein to stay attached to microtubules even under strong hindering forces (24–26). Together, these molecular adaptations allow multiple dyneins to coordinate their movement and combine their forces (11,19,21).

In living cells, dynein likewise promotes robust and processive retrograde transport of organelles (11,27,28). In addition to the molecular adaptations enabling multiple dyneins to coordinate their stepping (11), dynein clusters into teams on the organelle's surface (28,29). One of these dynein teams then interacts with a single microtubule so that all dynein molecules in this team contribute to the force production (28). For example, multiple dyneins on intraflagellar transport trains can jointly exert forces of more than 60 pN (30,31). Furthermore, cytoplasmic dynein in living cells can produce retrograde transport forces of about 2–20 pN, also indicating the activity of multiple dynein complexes (11,32–35).

All in vivo studies focused on small organelles with a size of 1 μm or smaller (for example, latex bead compartments (11,32–34) and lipid droplets (35)). However, many organelles that are transported by dynein are much larger, such as nuclei (5,36) or some mitochondria (37). Especially in phagocytic cells, which clear pathogens by phagocytosis, robust transport of the phagosomes is crucial for their degradation (38–40). The size of the phagosomes is determined by the size of the internalized object. Often, the phagosomes contain bacteria (41), aggregates of bacteria (42), and particulate contaminants such as microplastic particles (43). Therefore, phagosomes span a large size range of 0.5 μm (small bacteria, atmospheric particulate pollution) to more than 20 μm (bacterial aggregates, microplastic particles) (39,42,44,45).

For organelles with a diameter of several micrometers, cytoplasmic viscosities in the order of 10–100 Pa s have been reported (46,47). Consequently, drag forces on the order of several 100 pN can oppose the intracellular transport of larger organelles (38,48). These drag forces slow down average transport velocities of μm-sized cargoes to 20–60 nm s^{-1} (38,40) compared to the 500–1000 nm s^{-1} that was measured for dynein in vitro (14,23,49). Due to these strong drag forces, the force generated by one team of dyneins moving on a single microtubule are insufficient to overcome the viscous drag of the cytoplasm for larger organelles. Nevertheless, there is fast transport of organelles of different sizes in living cells. For example, the retrograde transport of larger 3-μm organelles is even more persistent than the retrograde transport of smaller 1-μm organelles, which show a very bidirectional motion (38). Possibly, the numbers of involved motors and the resulting transport forces are responsible for this size-dependent transport behavior. However, up to now, neither the magnitude of the transport forces for large cargoes nor their dependence on the cargo size are known. Furthermore, it is currently not known how dyneins work together to generate forces high enough to drive the transport of such large organelles.

We investigated dynein's ability to collectively drive the transport of large organelles. Using magnetic tweezers, we measured transport forces during the retrograde motion of phagosomes with a diameter of 1–5 μm in living J774A.1 macrophages. Furthermore, we analyzed the motion of the phagosomes to determine the viscous drag opposing retrograde transport of these organelles. To connect the observed transport forces to the underlying molecular machinery, we labeled dynein on isolated phagosomes and quantified the motor numbers using stepwise photobleaching. With a theoretical model taking the displacement of microtubules by the phagosomes into account, we linked the observed transport forces with the corresponding dynein numbers for the phagosomes of different sizes. Our results suggest that, on large organelles, hundreds of dyneins cluster into many teams that likely interact with multiple microtubules. The high forces generated by these dynein teams are required to overcome the drag of the cytoplasm, enabling robust and fast retrograde transport of the organelles.

MATERIALS AND METHODS

Cell culture

J774A.1 cells (DSMZ, Braunschweig, Germany) were cultured in 25-cm^2 flasks (BioLite Cell Culture Treated Flasks, Thermo Fisher Scientific, Waltham, MA) at 37°C, 5% CO_2, humidified, in DMEM (Gibco life technologies, Carlsbad, CA) with 10% (v/v) fetal bovine serum (Gibco life technologies, Carlsbad, CA) and 1% penicillin/streptomycin (Thermo Fisher Scientific, Waltham, MA). Cells were passaged three times per week. After 35 passages, the cells were replaced.

Bead opsonization

Carboxylated magnetic microbeads (Dynabeads MyOne, $D = 1$ μm, catalog no. 65011, Thermo Fisher Scientific, Waltham, MA; Dynabeads M-270, $D = 2.8$ μm, catalog no. 14305D, Thermo Fisher Scientific, Waltham,

MA; micromer-M, $D = 2$, 4, and 5 μm, catalog nos. 08-02-203, 08-02-403, and 08-02-503, micromod, Rostock, Germany) were opsonized with mouse serum immunoglobulin (Ig)G (EMD Millipore, Darmstadt, Germany) following the procedure in (38,50). Opsonization was verified by immunofluorescence (DyLight-488 goat anti-mouse secondary antibody, Thermo Fisher Scientific, Waltham, MA).

Magnetic tweezers

The setup, previously described in (51), consists of an inverted microscope (Nikon Eclipse Ti-U, Nikon, Japan) placed on an optical table (PFA51504, Thorlabs, Newton, NJ) with a 60× oil immersion objective (CFI Plan Apochromat λ 60× oil objective, NA 1.40, Nikon) and a 20× objective (CFI Plan Achromat 20× objective, NA 0.4, Nikon). Images were acquired using a CMOS camera (Orca Flash V4.0 v2, Hamamatsu, Shizuoka, Japan) under bright-field illumination. The magnetic tweezers consist of a solenoid with a high-permeability soft-iron core that is sharpened to a tip to generate a magnetic gradient field. The coil consists of 1420 loops of a 0.5-mm-thick copper wire looped around a macrolon cylinder with a length of 50 mm and a radius of 10 mm. The μ-metal core (Vacuumschmelze Hanau, Germany) has a length of approximately 14.5 cm and a diameter of 5.5 mm. The tip was sharpened to an angle of 40°. The magnetic tweezers were operated at a coil current of 0.1 A (PSI18032-10T, Elektroautomatik, Germany). To this setup, high-precision linear stages (Physik Instrumente, Karlsruhe, Germany: L511 20DG10, x and y position; M403 10DG, z position) for positioning of the tip were added, allowing automated movement and tracking of the tip position during measurements. The force exerted on a magnetic bead was regulated by adjusting the distance between tip and bead. Samples were kept at 37°C during measurements using a custom-built heating system. Evaporating water was replaced at a rate of 180 μL h^{-1} by a syringe pump (LA 30, Landgraf Laborsysteme, Germany). The magnetic force on the beads F_{MT} depended on the tip-bead distance d and was calibrated with beads suspended in a glycerol-water mixture with known viscosity η. When moving toward the tweezers' tip, their velocity $v(d)$ was used to determine the magnetic force with Stokes' law of friction $F_{MT}(d) = 6\pi\,\eta\,R\,v(d)$. The calibration procedure is described in detail in the supporting material appendix.

Transport-force measurements

The day before the experiments, 40,000 cells were seeded onto a 22 × 40 mm^2 coverslip (VWR, Radnor, PA). Before the experiments, opsonized beads were diluted 1:1000 in image medium (MEM, 5% HEPES, 1% penicillin/streptomycin) and washed to remove detached IgG. 100 μL of this solution was added to a sample and incubated for 25 min to enable phagocytosis of the beads. The sample was mounted to the setup, and a cell with a suitable phagosome was chosen. The tip of the magnetic tweezers was positioned 25 μm above the phagosome. In this way, the magnetic force was directed parallel to the microtubules and therefore pulled in the opposite direction to the molecular motors (Fig. S1). The magnetic force F_{MT} was the projection of the three-dimensional magnetic force $F_{MT,3D}$ into the x-y plane (Fig. 1 A). The measurements were taken at 3–6 fps, and the phagosome position was tracked in real time using a cross-correlation tracker as previously described (38,52,53). If tracking failed due to changes in the focal plane, radial symmetry detection was used to resume tracking (54). Together with the tracking of the tip position, this enabled a live measurement of the tip-bead distance and therefore of the force acting on the phagosome. With a feedback loop, the tip position was automatically set for each frame of the measurement so that the force on the particle decreased linearly in time. A measurement started with a magnetic force F_{MT} higher than the expected stall force F_{stall}, pulling the phagosome into the cell periphery. During this phase of the measurement, we ensured that the magnetic forces were not higher than necessary to avoid potential damage to the

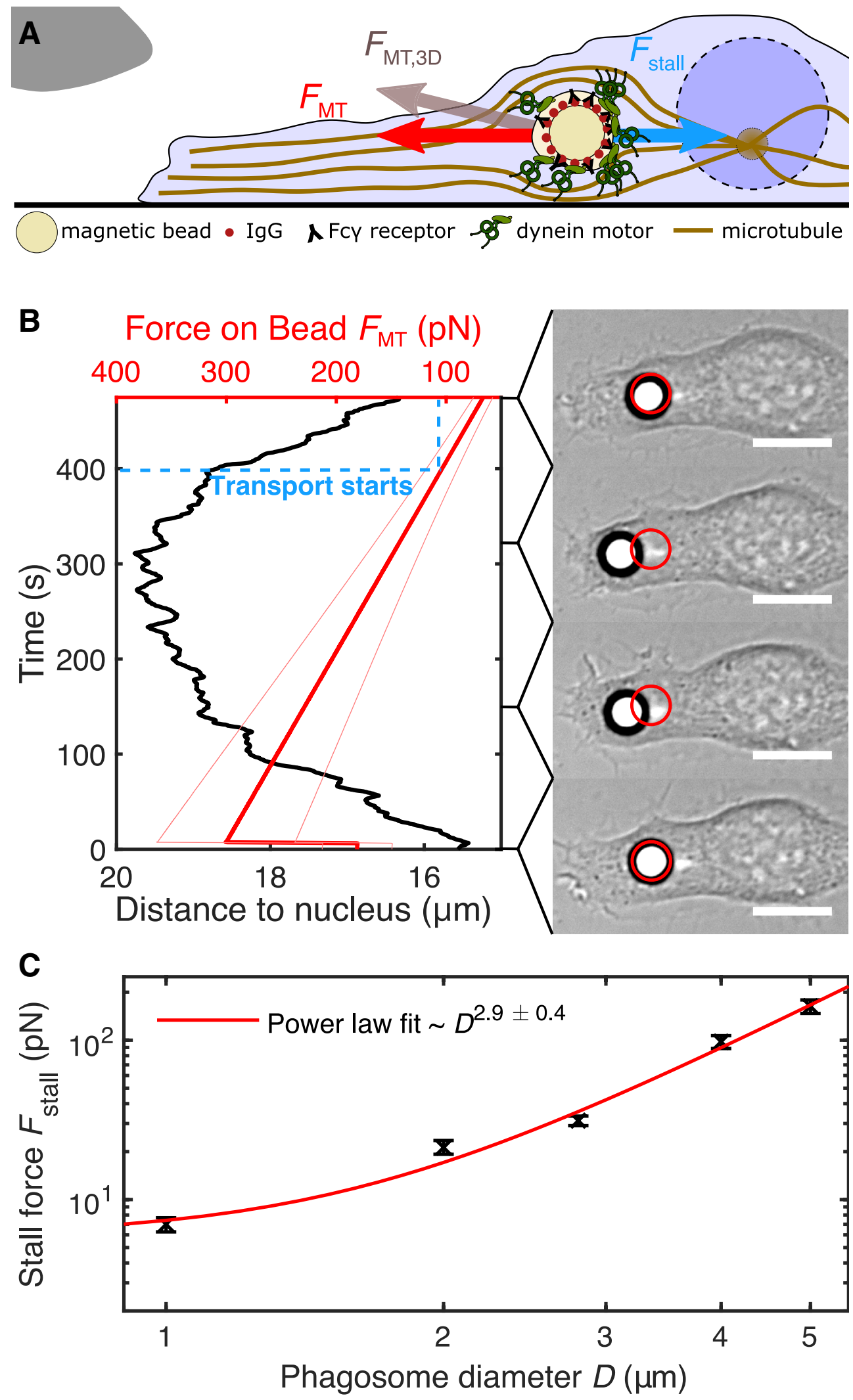

FIGURE 1 Stall-force measurements using magnetic tweezers. (A) In the magnetic gradient field generated by the magnetic tweezers, a force $F_{MT,3D}$ acted on these phagosomes. The projection onto the x-y plane of this magnetic force F_{MT} was directed against the force F_{stall} exerted by the molecular motors during retrograde transport of the phagosomes. At $F_{MT} \approx -F_{stall}$, the angle between $F_{MT,3D}$ and F_{MT} was approximately 10°–15°. (B) Here, an example of a stall-force measurement is shown. At the beginning of each measurement, the phagosomes were pulled into the cell periphery with a high magnetic force F_{MT}. Then, we lowered the force on the phagosome continuously until the force balance $F_{MT} \approx -F_{stall}$ was crossed and the retrograde transport of the phagosome started. By identifying the onset of transport, we determined the stall force of the phagosome F_{stall}. The red circle in the bright-field images of the measurement marks the original position of the phagosome. Scale bar: 10 μm. (C) The measured stall forces strongly depended on phagosome size. We measured average stall forces between 7.0 pN (95% confidence interval: 6.3–7.7 pN) (1-μm phagosomes) and 160 pN (95% confidence interval: 150–180 pN) (5-μm phagosomes). A power-law fit revealed a scaling exponent of 2.9 ± 0.4 of the stall forces F_{stall} with the phagosome diameter D. The fit included a constant offset to acknowledge that at least one force-producing unit was active during stall-force measurements due to our measurement method. Sample size $n = 116$ (1-μm phagosomes), 73 (2 μm), 67 (2.8 μm), 46 (4 μm), and 44 (5 μm). Error bars represent the 95% confidence interval estimated by bootstrapping.

microtubule cytoskeleton. Usually, forces were chosen such that the velocity of anterograde phagosome movement was not substantially higher than that of the natural retrograde motion mediated by dynein.

After phagosomes were pulled into the cell periphery, the force was decreased constantly over a period of about 5 min, until the maximum tip-bead distance of 500 μm was reached. This relatively long time between reaching the maximal F_{MT} and the onset of transport ensured that, even if damage to microtubules occurred, they had time to either self-repair (55–57) or be replaced by new microtubules (58,59). We defined transport as the movement of a phagosome toward the nucleus that persisted for at least 1 μm. Measurements in which disturbances such as cell migration or drastic changes in cell morphology occurred were excluded, as illustrated in Fig. S2. The time point at which transport toward the nucleus started corresponds to the force balance $F_{\mathrm{stall}} = -F_{\mathrm{MT}}$, enabling us to determine the stall force F_{stall}. Although during retrograde transport of phagosomes some fluctuations of phagosomes in the y direction occurred, these were always small compared to the transport along the x direction (compare, e.g., Video S3). Furthermore, no considerable motion along the z axis, which would lead to defocusing of the phagosomes, occurred.

Because we quantified retrograde stall forces by observing retrograde transport of phagosomes, we could not measure stall forces of zero, because at least one force-producing unit had to be actively driving retrograde transport. In particular, we did not include stall-force measurements from phagosomes without retrograde transport, since the absence of movement could have multiple causes beyond a stall force. Most importantly, phagosomes that showed no retrograde transport after moving them toward the cell periphery were potentially not completely internalized. Because stall-force assays were done in untransfected, unlabeled cells, we could not directly confirm internalization (e.g., via F-actin dynamics during phagocytosis) and instead relied on active transport as an indicator.

Cytoplasmic drag

Unloaded phagosome velocities have been shown to be much lower than the velocity of individual dyneins in in vitro assays (38,40), suggesting that motor forces are balanced by viscous drag forces of the cytoplasm. We aimed to quantify the cytoplasmic drag by analyzing the phagosomes' movement in cells. The average transport velocity v_{avg} was determined by fitting a line to the organelle trajectories in time intervals of 30 s, yielding v_{avg} as a function of the average magnetic force $F_{\mathrm{MT,avg}}$ exerted on the organelle during that time interval (Fig. S3). Assuming Stokes' law of friction, the average velocity depends linearly on the average force $F_{\mathrm{MT,avg}}$:

$$v_{\mathrm{avg}}\left(F_{\mathrm{MT,avg}}\right) = \frac{1}{3\pi\,\eta_{\mathrm{eff}}\,D}\left(F_{\mathrm{stall}} - F_{\mathrm{MT,avg}}\right). \qquad (1)$$

This equation was fitted to obtain the effective cytoplasmic drag η_{eff} and to verify the analysis of the average stall force F_{stall}. The uncertainty of the fitted parameters was estimated by bootstrapping. For each phagosome size, random subsets of 100 data points were sampled from all data points and fitted with Eq. 1. This was repeated 1000× each, which was sufficient for the mean of all fit results to converge. The standard deviation of the fitted parameters corresponded to their uncertainty.

siRNA knockdown of dynein

We reduced dynein expression with a small interfering RNA (siRNA) knockdown of the dynein intermediate chain *dync1i2*. A day before treatment, 80,000 cells were seeded on 18-mm coverslips in a 12-well plate. Per well, 3 μL of the transfection reagent (Lipofectamine RNAiMAX, Thermo Fisher Scientific, Waltham, MA) was diluted in 50 μL of Opti-MEM medium (Thermo Fisher Scientific, Waltham, MA). Then, per well, 1 μL of 10 μM siRNA stock solution (*dync1i2* siRNA: Silencer Select pre-designed siRNA, siRNA ID s65062, sequence (sense strand): 5′-GCA UCGAGUUGUUAGUUGUtt-3'. Negative-control siRNA: Silencer Select Negative Control #2 siRNA (sequence undisclosed). Thermo Fisher Scientific, Waltham, MA) was added to 50 μL of Opti-MEM, combined with the diluted transfection reagent and incubated for 5 min at room temperature (RT). Finally, 100 μL of this transfection solution (10 pmol siRNA) was added to each well and incubated for 48 h.

Dynein expression was quantified by western blotting following standard western blot protocol (given in the supporting material appendix) and using a mouse anti-dynein intermediate-chain primary antibody (Invitrogen Dynein Monoclonal Antibody (74.1) MA1-070, Thermo Fisher Scientific, Waltham, MA) and mouse anti α-tubulin antibody as loading control (alpha-tubulin (DM1A) mouse monoclonal antibody, Cell Signaling Technology, Cambridge, UK). The secondary antibody was horseradish peroxidase-conjugated goat anti-mouse (Immun-Star Goat Anti-Mouse (GAM)-HRP Conjugate #1705047, Bio-Rad Laboratories, Hercules, CA).

Ciliobrevin D treatment of cells

Complementary to the siRNA knockdown, we measured transport forces in cells treated with ciliobrevin D (EMD Millipore, Merck, Darmstadt, Germany), a small-molecule inhibitor of the ATPase activity of dynein. 20 min before an experiment, the medium in the samples (prepared analogously to the wild-type samples) was exchanged for image medium containing 50 μM ciliobrevin D. At this concentration, the ATPase activity of dynein is reduced to about 50% (60).

Phagosome isolation

For the isolation of phagosomes, we followed the procedure described in (34,61). Cells were seeded in four culture flasks and grown to 75% confluency. Before the procedure, 500 μL of the respective carboxylate-bead stock solution (micromer-M, $D = 2$ μm, 3 μm, 4 μm, 5 μm, micromod, Rostock, Germany; 1- and 2.8-μm Dynabeads were excluded due to strong autofluorescence) was washed 3× in PBS and dispersed in 11.5 mL of cell culture medium. The old medium was replaced with 3 mL of the bead-medium solution per flask. The cells were incubated for 60 min at 37°C and 5% CO_2 to allow phagocytosis. After incubation, the cells were washed with PBS to remove unphagocytosed particles. Cells were scraped from the flasks and centrifuged at 200 × g for 2 min. The supernatant was discarded, and the cells were suspended in 1 mL of HB (homogenization buffer, consisting of motility assay buffer [MAB; 10 mM PIPES, 50 mM K-Acetate, 4 mM $MgCl_2$, and 1 mM EGTA; pH 7.0] with 10% sucrose, 1× cOmplete EDTA-free protease inhibitors [Sigma Aldrich, Darmstadt, Germany], 10 μM ATP, and 30 μM DTT) . The cells were homogenized by pressing them 40–50 times through a syringe with a 27G needle monitored under a bright-field microscope. After sufficient homogenization, the phagosomes were separated using a strong magnet and washed 3× in HB with all steps performed on ice to maintain protein integrity. Finally, the isolated phagosomes were suspended in 600 μL of homogenization buffer, aliquoted into 30 μL, and stored at −80°C until needed.

Immunofluorescence of dynein

Before the immunofluorescence experiments, coverslips were treated with a hydrophobic coating. Coverslips were cleaned for 10 min in acetone, sonicated for 20 min in methanol, sonicated for another 20 min in 0.5 M potassium hydroxide (KOH), and rinsed 3× with MilliQ water before drying overnight. Dried coverslips were soaked in Rain X 26014 rain repellent for 5 min, dried for 15 min, and gently rubbed with a Kimwipe. The hydrophobicity was checked by placing a small droplet of MilliQ water on a treated coverslip, showing contact angles of 90°–100°, indicating a successful hydrophobic treatment. Then, small flow channels were assembled from the coverslips and the phagosomes flowed into the flow channels, fixed with

4% paraformaldehyde, and labeled with the primary (Invitrogen Dynein Monoclonal Antibody (74.1) MA1-070, Thermo Fisher Scientific, Waltham, MA) and secondary antibody (Invitrogen Goat Anti-Mouse IgG, DyLight 488 conjugated, Thermo Fisher Scientific, Waltham, MA) following standard protocol (supporting material appendix).

Imaging was performed using a Epi/ total internal reflection fluorescence (TIRF) microscope (Leica Microsystems, Wetzlar, Germany) with an HC PL Apo 100×/1.47 NA oil immersion objective (Leica) and a Leica DFC 9000 GT sCMOS (scientific complementary metal-oxide semiconductor) camera. The samples were illuminated using a 488-nm laser at 4.4-mW power and imaged with an exposure time of 500 ms and 2 × 2 binning.

We validated our immunofluorescence assay by completely replicating our experiments with another batch of isolated phagosomes, different types of primary and secondary antibodies, a different fluorophore, and a different microscopy setup (Fig. S4). In addition, we labeled kinesin-2. An overview of the replication experiment is given in the supporting material appendix.

Dynein quantification

The phagosomes were imaged in the epifluorescence mode of the microscope. Since, occasionally, individual phagosomes were not completely isolated from cell debris, they were very bright and overexposed. These phagosomes were not considered in the evaluation. The integrated dynein intensity I_{dyn} was measured in a circular region of interest with a diameter of $1.5 \times D$. For each phagosome, the background intensity was measured in a similarly sized region of interest nearby and subtracted from the dynein signal. Unspecific binding of the primary antibody to the carboxylate beads was corrected by subtracting the fluorescence of the stained unphagocytosed beads from the dynein fluorescence. The values for I_{dyn} are provided in Table S4.

For each phagosome sample, photobleaching experiments were performed by imaging the field of view for 10 min with a rate of 2 fps. We detected steps in the bleaching curves using an algorithm established by Chen et al. (62), based on a two-sample *t*-test to identify time intervals with constant mean and variance, fitting a piecewise constant function to the photobleaching curves (Figs. S5 *A*, *B*, and S6). The resulting step-size distributions (Fig. S5, *C* and *D*) were fitted using a Gaussian mixture model (34,62), yielding a unitary step size $I_{\mathrm{unitary}}^{\mathrm{epi}} = (160 \pm 30)$ arbitrary units and $I_{\mathrm{unitary}}^{\mathrm{TIRF}} = (235 \pm 50)$ arbitrary units. Details of the Gaussian mixture model fit are given in the supporting material appendix. We validated the photobleaching procedure by determining the degree of labeling of the secondary antibodies by stepwise photobleaching and comparing this value to the degree of labeling measured spectroscopically (supporting material appendix).

We then quantified the number of fluorophores bound to a primary-secondary antibody complex. We diluted the primary antibodies 1:10,000 in 60 μL of MAB, flowed them into the sample chamber, and let them attach to the coverslip overnight at 4°C. Then, we blocked the coverslip surface with Pluronic F-127. We incubated the sample for 45 min at RT with the secondary antibodies, in the same way that we treated the phagosome samples (Fig. S5 *G*).

The samples were imaged in the TIRF mode of the microscope. We measured the fluorescence intensity of each individual spot on the coverslip and corrected it for background. Then, we calculated the number of fluorophores in a single primary-secondary antibody complex:

$$N_{\mathrm{pAB}} = \frac{I_{\mathrm{pAB}}}{I_{\mathrm{unitary}}^{\mathrm{TIRF}}}. \qquad (2)$$

On average, we found that 70 ± 40 fluorophores were bound to a single primary antibody (Fig. S5 *H*). The number is higher than in previous studies because multiple secondary antibodies bind to one primary antibody.

With the number of fluorophores per primary/secondary antibody complex N_{pAB}, the total intensity of a single dynein-antibody complex is $I_{\mathrm{singledyn}} = 2 \times N_{\mathrm{pAB}} \times I_{\mathrm{unitary}}^{\mathrm{epi}}$, since dynein has two copies of the intermediate chain and thus two binding sites for the monoclonal primary antibody. Consequently, the number of dyneins on a phagosome is $N_{\mathrm{dyn}} = I_{\mathrm{dyn}}/I_{\mathrm{singledyn}}$. Examples of photobleaching curves, phagosomes, their corresponding intensities, and motor numbers are shown in Fig. S6.

Furthermore, we prepared samples in which we stained the membrane of the isolated phagosomes with FM 4-64 (Thermo Fisher Scientific, Waltham, MA). The intensity of the phagosomal membrane I_{mem} was evaluated analogously to the dynein intensity I_{dyn}. We evaluated the scaling behavior of the motor-area density by evaluating $I_{\mathrm{dyn}}/I_{\mathrm{mem}}$ for all phagosome diameters D.

Confocal images of the microtubule skeleton

100,000 cells were seeded on an 18-mm round coverslip (VWR, Radnor, PA) 1 day before the sample preparation. The beads (D = 2, 4, and 5 μm) were washed 3× in PBS, diluted 1:1000 in cell culture medium, and 100 μL of the diluted bead solution was added to the cells and incubated for 60 min to allow phagocytosis. Then, cells were washed 3× with PBS, fixed with 4% paraformaldehyde in PBS for 15 min at RT, and labeled with the primary antibody (α-tubulin (DM1A) mouse monoclonal antibody, Cell Signaling Technology, Cambridge, UK) and secondary antibody (Invitrogen goat anti-mouse IgG, DyLight 488 conjugated, Thermo Fisher Scientific, Waltham, MA) according to standard protocol (supporting material appendix). Imaging was performed using a custom-built spinning-disk confocal microscope (63) consisting of a Leica DMI 6000 body (Leica Microsystems, Wetzlar, Germany) equipped with a CSU-X1 spinning-disk unit (Yokogawa Microsystems, Tokyo, Japan), a HC PL APO 100×/1.4 NA oil immersion objective (Leica Microsystems), and a Photometrics Evolve 512 charge-coupled device camera (Photometrics, Tucson, AZ). The images show the displacement and bending of the microtubule cytoskeleton caused by the phagosomes without an external magnetic force.

Modeling of elastic forces due to deformations of the cell cortex

We quantified possible forces due to elastic deformations (Fig. S7 *A*) of the cell cortex by the phagosomes by modeling the deformations heuristically as cosine shaped with smooth transitions to the undeformed cell at its edges and a radius of curvature that is equal to the bead radius at its maximum (Fig. S7, *B* and *C*). For a bead with radius $R = D/2$ that is at position x_{B} along the x axis, the deformation of the cell cortex along the x' axis can be written as

$$f(x') = h\left[\frac{1}{2}\cos\left(\frac{1}{a}\left(x' - x'_{\mathrm{B}}\right)\right) + \frac{1}{2}\right], \qquad (3)$$

where h is the height of the deformation perpendicular to the undeformed cortex, and πa is the radius of the deformed region of the cell cortex. To determine the width a of the deformation, we used the condition that the radius of curvature of the deformation at $x' = x'_{\mathrm{B}}$ must be equal to the bead radius R.

Assuming that the deformation of the cell cortex is rotationally symmetrical around x'_B, we calculated the area of the deformed cell cortex. With the known tension of the cell cortex of J774A.1 cells $\sigma = 140 \pm 20$ pN μm^{-1} (64), we determined the energy $W_{\mathrm{deformation}}$ required for a given deformation. The force that pushes the bead along the x axis is the spatial derivative of this energy, which was evaluated numerically:

$$F_{\mathrm{Actin}} = -\frac{\partial}{\partial x_{\mathrm{B}}} W_{\mathrm{deformation}}. \qquad (4)$$

A step-by-step derivation of the model is given in the supporting material appendix.

Creep-recovery measurements

To analyze creep of the cell cortex under sustained stress, samples were prepared in the same way as for the transport-force measurements. The 2.8-μm beads opsonized with IgG were added to the cells, incubated for 25 min to enable phagocytosis, the sample was mounted to the magnetic tweezers setup and a suitable cell was chosen. Then a measurement was started and images were acquired with a frame rate of 3–6 fps. During the measurements, real-time tracking was used to follow the phagosome's position. Using the feedback loop of the magnetic tweezers, the MT tip was positioned automatically to keep the force on the phagosomes constantly at 100 pN. The current of the magnetic tweezers was turned on and off periodically with a period of 60 s to measure the step response of the system (magnetic force on, creep; magnetic force off, recovery).

RESULTS

Transport forces increase with the third power of the organelle diameter

Murine macrophages were incubated with IgG-opsonized magnetic beads to generate phagosomes with a defined diameter D. We measured the stall forces F_{stall} of these phagosomes during their retrograde transport using magnetic tweezers (Figs. 1 *A*, *B*, S2, and S9; Videos S1, S2, S3, S4, and S5). To this end, phagosomes containing magnetic beads were first pulled away from the nucleus toward the cell periphery with a high magnetic force F_{MT}. We positioned the tip of the magnetic tweezers in a line with the cells' long axes, such that the magnetic forces and phagosome motions were parallel to the orientation of the microtubule cytoskeleton (Fig. S1) and opposite to the direction of motion of dynein (65). Then, the magnetic force was gradually lowered until transport of the phagosome started. At the onset of transport, the force balance between the magnetic force and the stall force generated by the molecular motors $F_{\text{MT}} = -F_{\text{stall}}$ was crossed, which allowed us to quantify F_{stall} (Figs. 1, *B* and S2). The results of the force measurements for phagosomes with diameters D of 1, 2, 2.8, 4, and 5 μm (Fig. 1 *C*) are summarized in Table 1. We found that the stall forces of the phagosomes strongly depended on their size. For the smallest phagosomes, we measured an average transport force of 7.0 pN (95% confidence interval: 6.3–7.7 pN). The forces continuously increased with phagosome size up to 160 pN (95% confidence interval: 150–180 pN) for the largest phagosomes.

TABLE 1 Summary of the Measured Stall forces F_{stall}, Effective Cytoplasmic Viscosities η_{eff}, and the Number of dyneins per Phagosome for all Phagosome Sizes

D (μm)	F_{stall} (pN)	η_{eff} (Pa s)	N_{dyn}
1	7.0 (6.3–7.7)	10 ± 7	–
2	21 (19–23)	25 ± 10	30 (10–50)
2.8	31 (29–33)	28 ± 3	60 (0–140)
4	100 (90–110)	100 ± 30	150 (40–260)
5	160 (150–180)	100 ± 20	240 (70–410)

F_{stall} and number of dyneins per phagosome are presented as mean and 95% confidence interval estimated by bootstrapping.

To quantify the size dependence of the observed stall forces, we fitted a power law of the form

$$F_{\text{stall}}(D) = a \times \left(\frac{D}{1\ \mu\text{m}}\right)^{\alpha} + c. \quad (5)$$

We found a scaling exponent α of 2.9 ± 0.4. For the scaling factor a, we observed a value of (1.5 ± 0.5) pN, and for the offset c a value of (6 ± 4) pN. The offset was included to acknowledge that at least one force-producing unit was active during stall-force measurements due to our measurement method.

High transport forces are necessary to overcome the drag of the cytoplasm

To enable robust and fast intracellular transport of organelles, the forces generated by the molecular motors need to be high enough to overcome the drag of the cytoplasm. We therefore determined the effective cytoplasmic drag coefficient η_{eff} by analyzing the average transport velocity v_{avg} of the phagosomes in the transport-force measurements as a function of the average magnetic force $F_{\text{MT,avg}}$ over intervals of 30 s (Fig. S3). In these measurements, the phagosomes were first pulled toward the cell periphery with a high magnetic force $F_{\text{MT,avg}}$ (increasing distance to the nucleus, $v_{\text{avg}} < 0$). Then, the magnetic force was gradually lowered, and dynein-mediated transport to the nucleus was started (distance to the nucleus decreased, $v_{\text{avg}} > 0$). In each 30-s interval, the magnetic force $F_{\text{MT,avg}}$ changed only slightly and was assumed to be constant. Overall, $v_{\text{avg}}(F_{\text{MT,avg}})$ approximately followed a linear relationship (Fig. S3 *B*; Fig. 2 *A*–*E*). We fitted these data with Stokes' law of friction (Eq. 1) to obtain the stall force and to quantify the cytoplasmic drag. Since the cytoplasm is not a simple Newtonian fluid, we had to introduce an effective cytoplasmic drag coefficient η_{eff}, which depends on the phagosome diameter D (Table S1).

The average stall forces $F_{\text{MT,avg}}(v_{\text{avg}} = 0)$ extracted from this analysis agree with the stall forces extracted directly from the individual phagosome trajectories. From the cytoplasmic viscosity analysis, we determined stall forces (Table S1) between (11 ± 10) pN for the smallest phagosomes ($D = 1\ \mu$m) and (170 ± 40) pN for the largest phagosomes ($D = 5\ \mu$m). These stall forces provide a verification of the first analysis method since they do not require the identification of the onset of transport. Therefore, these results confirm the stall-force values shown in Table 1. Here, the relatively large uncertainty of the stall force observed for the 1-μm phagosomes was likely related to their low transport persistence, which was also reported previously (38). Due to the lower persistence of retrograde motion, individual $v_{\text{avg}}(F_{\text{MT,avg}})$ data points had a larger variance for the 1-μm phagosomes compared to larger phagosomes, and therefore fitting Eq. 1 was associated with larger uncertainties.

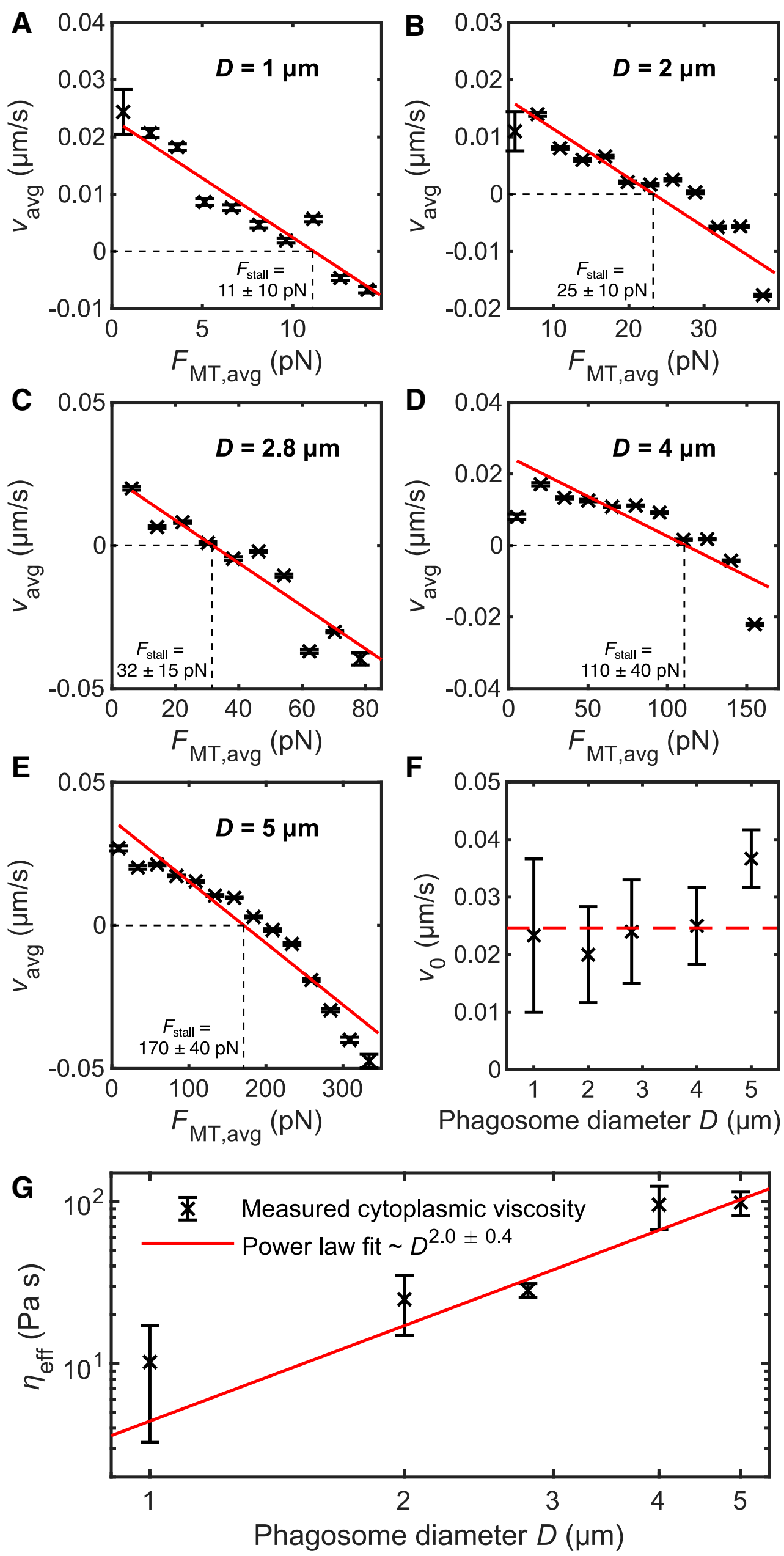

FIGURE 2 Analysis of the cytoplasmic drag. (*A–E*) We determined the effect of the drag of the cytoplasm during intracellular organelle transport by analyzing the average transport velocity v_{avg} as a function of the average magnetic force $F_{\mathrm{MT,avg}}$. The $v_{\mathrm{avg}}(F_{\mathrm{MT,avg}})$ data followed approximately a linear relationship, which we fitted with Stokes' law of friction (Eq. S1, red lines). From the fit we obtained the average stall forces and the effective cytoplasmic drag. The average stall force $F_{\mathrm{MT,avg}}(v_{\mathrm{avg}} = 0)$ is very consistent with F_{stall} obtained from the individual analysis of the trajectories (Table 1), supporting the validity of our transport criterion. Sample size $n = 840$ derived from 116 individual trajectories (1-μm phagosomes), 1613 from 73 (2 μm), 862 from 67 (2.8 μm), 977 from 46 (4 μm), and 764 from 44 (5 μm). For clarity, data points were displayed as average over intervals of 1.5 pN (1-μm phagosomes), 3 pN (2 μm), 8 pN (2.8 μm), 15 pN (4 μm), and 25 pN (5 μm). Error bars represent the SE of mean. (*F*) The free-transport velocity $v_0 = v_{\mathrm{avg}}(F_{\mathrm{MT,avg}} = 0)$ did not systematically depend on the diameter *D*. The average v_0 was (25 ± 4) nm s^{-1}. (*G*) The effective cytoplasmic drag η_{eff} strongly depended on *D*. We observed effective cytoplasmic drag coefficients between (10 ± 7) Pa s and (100 ± 20) Pa s. The scaling exponent of the size-dependence of η_{eff} was 2.0 ± 0.4.

The free-transport velocity $v_0 = v_{\mathrm{avg}}(F_{\mathrm{MT,avg}} = 0)$, which is the transport velocity of the organelles without an external load, did not systematically depend on phagosome size (Fig. 2 *F*; Table S1). We measured an average free-transport velocity of (25 ± 4) nm s^{-1}. Similar studies on retrograde transport of μm-sized cargoes consistently reported similar average transport velocities of 20–60 nm s^{-1} (38,40).

In contrast, the effective cytoplasmic drag η_{eff} depended strongly on the phagosome size (Fig. 2 *G*; Table 1), which is likely related to the fact that the cytoplasm is a complex fluid (48,66). Although the $v_{\mathrm{avg}}(F_{\mathrm{MT,avg}})$ relationship heuristically followed Stokes' law of friction, the size dependence of the drag coefficient obtained in this way indicated the non-Newtonian nature of the cytoplasm. We assume that this size dependence of the drag coefficient was caused by a combination of viscous forces of the cytoplasm and inelastic deformations of elements, such as actin filaments and microtubules; however, more research would be needed to obtain a mechanistic model of this cytoplasmic drag coefficient η_{eff}. Nevertheless, for the smallest phagosomes, we observed an effective cytoplasmic viscosity of (10 ± 7) Pa s, which continuously increased up to (100 ± 20) Pa s for the largest phagosomes. We quantified the size dependence of the cytoplasmic viscosity heuristically using a power law:

$$\eta_{\mathrm{eff}}(D) = d \times \left(\frac{D}{1\mu\mathrm{m}}\right)^{\beta}. \tag{6}$$

For the scaling factor *d*, we found (4.3 ± 1.3) Pa s. We observed a scaling exponent β of 2.0 ± 0.4, which agrees very well with the scaling behavior of the stall forces. Since the free-transport velocity v_0 was independent of *D*, the scaling behavior of the observed stall forces can be written as

$$F_{\mathrm{stall}}(D) \propto aD^{\alpha} \propto dD^{\beta} \times D \Rightarrow \alpha = \beta + 1 \tag{7}$$

using Stokes' law. Therefore, the independently observed scaling exponents α and β are very consistent. Overall, this analysis shows that the very high transport forces measured for the large organelles are necessary to overcome the drag of the cytoplasm.

Dynein is the main driver of retrograde phagosomal transport

To investigate the underlying molecular mechanisms of the observed transport forces, we examined the role of dynein during the retrograde transport of the phagosomes. We tested the hypothesis that dynein is primarily responsible for driving the observed retrograde organelle transport by reducing the expression level of dynein and by inhibiting its ATPase activity. We reduced dynein intermediate-chain

dync1i2 expression levels by 50% using RNAi (Fig. S10 *A*). The corresponding stall forces measured for phagosomes with a diameter of 5 μm were reduced to about 50% compared to wild-type cells and cells treated with negative-control siRNA. Consistently, the inhibition of dynein's ATPase activity with ciliobrevin D at a concentration at which the ATPase activity of dynein is reduced to about 50% (60) led to a 50% decrease in the stall force of the phagosomes (Fig. S10 *B*). Therefore, these results indicate that dynein is the main driver of retrograde transport of the phagosomes and that its force production is directly related to the number of active dynein complexes.

Alternatively, the measured forces during retrograde transport could originate from elastic deformations of the cell cortex by the phagosomes (Fig. S7 *A*). To evaluate this, we modeled the cortex deformations by phagosomes of different sizes (supporting material materials and methods; Fig. S7, *B* and *C*). With the known cortical tension of J774A.1 (64), we derived the maximal elastic force that might push the phagosomes toward the nucleus (Fig. S7 *D*). This maximal elastic force would only be reached if the cell had no possibility to adapt to extracellular forces (i.e., if it behaved as a purely elastic material). The elastic forces, dependent on phagosome size and position, ranged from 10 to 400 pN, similar to the measured transport forces. However, the elastic forces increased linearly with phagosome size, not matching the D^3 scaling of the measured transport forces (Fig. S7, *E* and *F*).

Furthermore, the cell cortex is not purely elastic but has both elastic and viscous properties (67–69), as also shown by our creep-recovery measurements (Fig. S11). We observed that, under a sustained magnetic force, the cell cortex is subjected to creep, which leads to dissipation of the cortical tension, so that the phagosome does not fully relax to its original position once the magnetic force is released. Our measurements indicate that this creep occurs on timescales of about 15–20 s, which coincides with the time for actin turnover and remodeling of the cell cortex (67,68). It is well established that, on timescales shorter than the actin turnover time, the cell cortex behaves like an elastic solid, and, on longer timescales, it transitions to a viscous behavior (67). In our measurements of the transport force, the time between reaching the maximal magnetic force and the onset of retrograde transport is much longer than the remodeling time of the cell cortex, ranging from 250 s (2.8-μm phagosomes) to 400 s (1- and 2-μm phagosomes). Therefore, we conclude that most elastic forces of the cell cortex were already dissipated at the onset of transport and only molecular motors contributed to the measured forces.

The dynein surface density does not depend on the phagosome diameter

Next, we aimed to connect the measured transport forces to the number of dyneins present on the phagosomes. To this end, we isolated phagosomes from the cells and immunofluorescently labeled dynein on their surface (Fig. S4). The dynein fluorescence on the phagosomes was enriched at discrete spots, indicating that dynein clustered into multiple dynein teams (Fig. 3 *A*), consistent with previous studies investigating the distribution of dyneins on organelles (28,29). We were able to replicate these experiments with another set of isolated phagosomes and different types of primary and secondary antibodies (Fig. S12). Additionally, we labeled kinesin-2 on the isolated phagosomes, which was reported to be present on isolated phagosomes previously (32,34). Kinesin did not form clusters on phagosomes (Fig. S12 *C*), indicating that the punctate localization is specific to dynein.

We quantified the scaling behavior of the amount of dynein attached to the phagosomes by evaluating their fluorescence intensity. The dynein fluorescence increased approximately with D^2 (Fig. 3 *B*). Fitting a power law $I = I_0 D^\gamma$ to the dynein fluorescence revealed a scaling exponent γ of 2.30 $\pm$ 0.15, which is distinct from the scaling exponent $\alpha = 2.9 \pm 0.4$ observed for the transport forces. We replicated this result with another set of isolated phagosomes and different types of primary and secondary antibodies and fluorophores (Fig. S13). To further exclude possible artifacts (e.g., size-dependent damage of the phagosomes caused by the isolation process), we additionally labeled the phagosomal membrane to quantify the amount of dynein per membrane area. We found that the dynein intensity per membrane intensity did not depend on *D*, indicating that the dynein surface density of the phagosomes is size independent (Fig. 3 *C*).

In addition to the scaling behavior of the amount of dynein, we also quantified the number N_{dyn} of individual dynein motors attached to phagosomes in the size range 2–5 μm using stepwise photobleaching (34,62) (Figs. S5 and S6). The stepwise photobleaching provides a calibration of the measured dynein intensity of the phagosomes to determine the absolute number of molecular motors. Therefore, the observed D^2 scaling behavior of the dynein numbers is independent of this calibration. We estimated 30 dyneins (95% confidence interval: 10–50) were bound to the smallest phagosomes ($D = 2$ μm). The number of motors continuously increased with increasing *D*, up to 250 dyneins (95% confidence interval: 70–410) for the largest phagosomes with a diameter of $D = 5$ μm (Table 1). On average, we measured a dynein surface density ρ_{Dyn} of 3 $\pm$ 1 dyneins μm^{-2}. Two earlier studies measured the numbers of dyneins attached to phagosomes isolated from J774 cells and observed similar dynein surface densities. For early phagosomes with a diameter of 0.5 μm, a dynein density of 4 dyneins μm^{-2} was found (65). Similarly, for late phagosomes, dynein densities of 3–6 dyneins μm^{-2} (34) and 6.5 dyneins μm^{-2} (65) were measured. These numbers further reinforce our hypothesis that the dynein surface density is independent of phagosome size over a large range (Fig. S14). The resulting force generated per

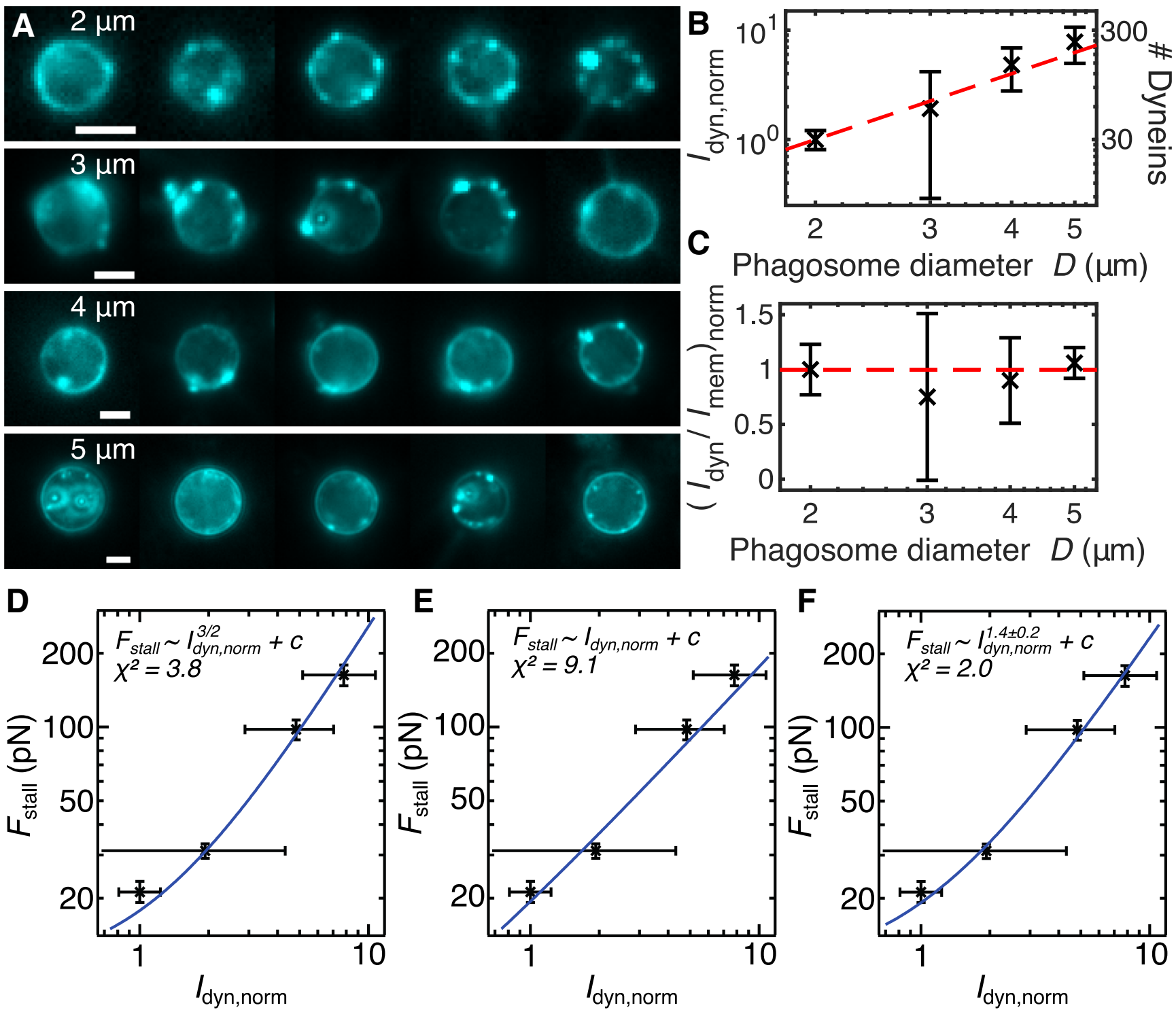

FIGURE 3 Labeling and quantification of dynein on isolated phagosomes. (*A*) Dynein forms several distinct clusters on phagosomes of all sizes, indicating that multiple teams of dyneins are present on the phagosomes' surfaces. Scale bars: 2 μm. (*B*) The normalized fluorescence intensity of the labeled dynein $I_{dyn,norm}$ increased with D^2, showing that the dynein number is proportional to the phagosome surface. Sample size $n = 92$ (2-μm phagosomes), 25 (3 μm), 31 (4 μm), and 22 (5 μm). Error bars represent the 95% confidence interval estimated by bootstrapping. (*C*) We confirmed the result by evaluating the fluorescence intensity of dynein relative to the fluorescence of the labeled phagosomal membrane I_{dyn}/I_{mem}. This ratio did not depend on D, indicating that the dynein surface density was independent of the phagosome size. Sample size $n = 182$ (2 μm phagosomes), 272 (3 μm), 224 (4 μm), and 327 (5 μm). (*D–F*) Force-motor scaling relationships fitted with $F_{stall} \sim I_{dyn,norm}^{3/2} + c$ (*D*), $F_{stall} \sim I_{dyn,norm} + c$ (*E*), and a free scaling exponent (*F*). The constant offset c was included to acknowledge that, due to our measurement method, at least one force-producing unit was active during stall-force measurements. The χ^2 goodness-of-fit values seem to suggest a better fit for $F_{stall} \sim I_{dyn,norm}^{3/2} + c$, which is consistent with the scaling exponent of 1.4 $\pm$ 0.2 for the free fit.

dynein $F_{dyn} = F_{stall}/N_{dyn}$ was approximately 0.5–0.7 pN, indicating that not all dyneins attached to a phagosome were active during retrograde transport. Similar to this result, Ucar and Lipowsky observed in simulations of collective force generation by up to 7 dyneins that the collective force increased linearly by ca. 0.7 pN per dynein (70).

To test whether the scaling behavior of the dynein numbers on the phagosomes and the transport forces were indeed distinct from each other, we analyzed the stall forces as a function of the dynein fluorescence. If $F_{stall} \sim D^3$ and $I_{dyn} \sim D^2$, then it should be expected that $F_{Stall} \sim I_{dyn}^{3/2}$. Otherwise, if the transport forces scaled purely with the number of dyneins attached to the organelles, as observed for small dynein numbers <10 dyneins, where all dyneins participate in the transport (11,70), then $F_{stall} \sim I_{dyn}$ should be expected. We fitted F_{stall} to both $I_{dyn}^{3/2}$ and I_{dyn} (Fig. 3, *D* and *E*). Like the power law fit in Eq. 5, these fits included a force offset, because our method required the phagosomes to be actively transported toward the nucleus to measure a stall force. Therefore, we always measured the stall force of at least one force-producing unit. Both the $F_{stall} \sim I_{dyn}^{3/2}$ and $F_{stall} \sim I_{dyn}$ fit intersect all datapoints within their margin of errors. However, the χ^2 goodness-of-fit values were more in favor of $F_{stall} \sim I_{dyn}^{3/2}$ (χ^2: 3.8) than of $F_{stall} \sim I_{dyn}$ (χ^2: 9.1). When leaving the exponent as a free fit parameter, the model converges to an exponent of 1.4 $\pm$ 0.2 (χ^2: 2.0), which is more consistent with $F_{stall} \sim I_{dyn}^{3/2}$ (Fig. 3 *F*). The replication dataset further supports a force-motor scaling with the exponent 3/2, with a χ^2 value of 4.5 for the $F_{stall} \sim I_{dyn}^{3/2}$ fit, compared to a χ^2 value of 17.7 for the $F_{stall} \sim I_{dyn}$ fit (Fig. S15).

Large organelles displace microtubules, enabling more dyneins to be active

To resolve this discrepancy, we developed a model that assumes that, in the cytoplasm, organelles displace microtubules from their original positions, increasing the microtubule density on the organelles' surface. Due to the microtubule displacement, more binding sites are available for dynein at the surface of larger organelles compared to smaller organelles that only interact with a few microtubules (Fig. 4 *A*). This assumption is backed by confocal images of the microtubule cytoskeleton. The smaller phagosomes (2-μm diameter) displaced only a few microtubules, barely increasing the microtubule surface density. However, the large phagosomes (5-μm diameter) displaced many more microtubules in the cytoplasm, leading to a visibly increased microtubule density at their surface (Fig. 4, *B* and *C*). Due to this increased microtubule density, more dyneins can actively contribute to transport forces of larger organelles. Our model assumes, consistent with previous results (70), that active dyneins combine their forces linearly.

In this paragraph, we explain the key ideas of the model. A step-by-step derivation of the model is provided in the supporting material materials and methods. The model assumes that the total number of microtubules that are displaced by a phagosome is proportional to the organelle's

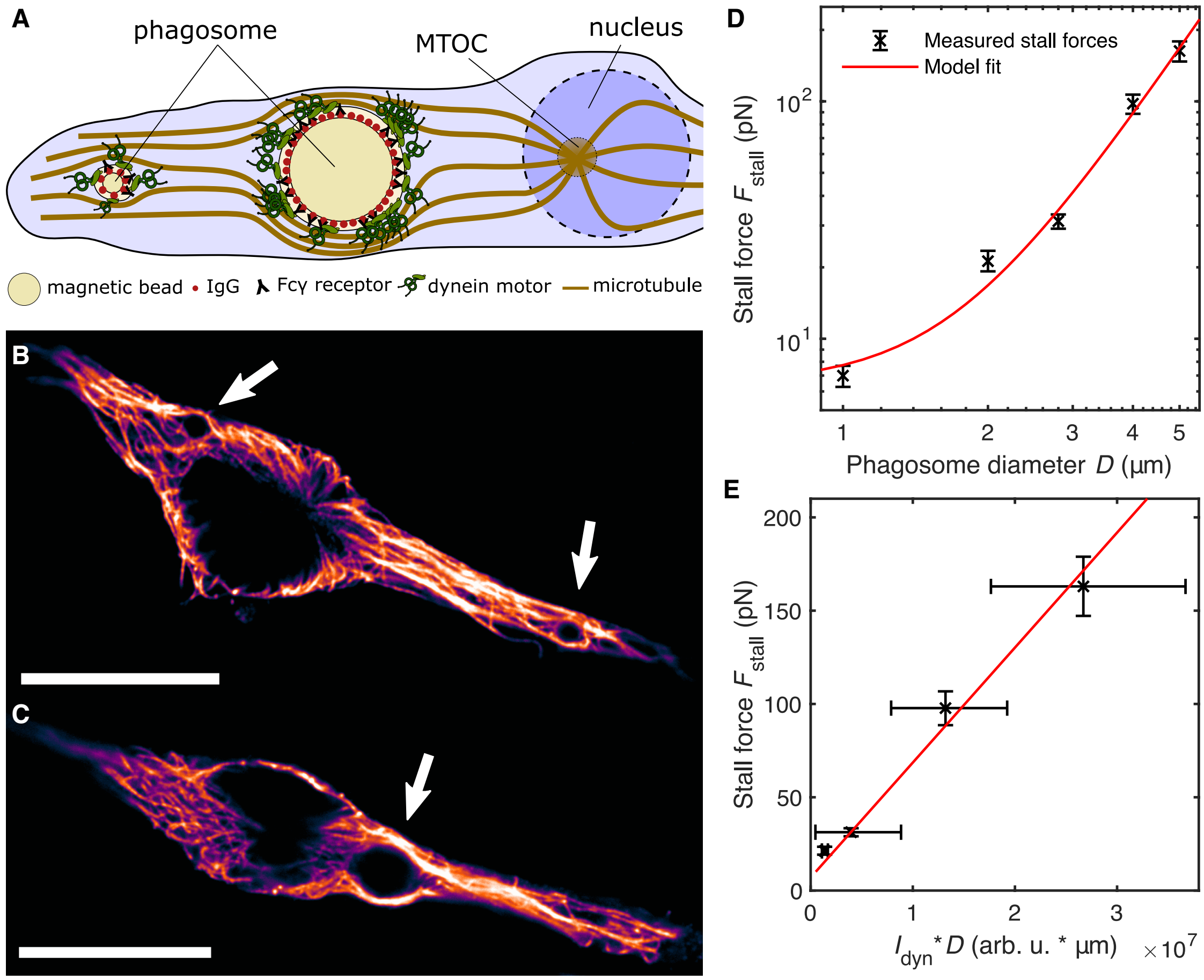

FIGURE 4 Modeling the displacement of microtubules. Because the number of dyneins attached to a phagosome only increases with D^2, whereas the stall forces increase with D^3, a larger fraction of the motors attached to larger phagosomes need to interact with microtubules to explain the observed scaling behavior of F_{stall}. (*A*) The enhanced motor-microtubule interactions can be explained taking the displacement of microtubules by the phagosomes into account. Larger phagosomes displace more microtubules compared to the smaller phagosomes, leading to an increased microtubule density at the phagosomes' surface. Therefore, more binding sites for dynein are available around the larger phagosomes. (*B* and *C*) In confocal images of the microtubule cytoskeleton of fixed cells (no external magnetic force), small phagosomes (white arrows) with $D = 2$ μm (*B*) only displaced a few microtubules, barely enhancing the microtubule density on their surface. In contrast, the large phagosomes (white arrow) with $D = 5$ μm (*C*) displaced many more microtubules in the cytoplasm, leading to a visibly increased microtubule surface density. Scale bar: 15 μm. (*D*) Modeling the displacement of microtubules yields $F_{stall}(D) = fD^3 + c_m$ for the stall forces. This simple model provides an explanation for the scaling behavior of the observed stall forces and fits the force data very well. We measured a force density f of (1.3 ± 0.1) pN μm^{-3} and an offset c_m of (7 ± 2) pN. (*E*) The same model can be written in terms of the measured dynein intensity on phagosomes (compare Eq. 12). Plotting data and model in this way demonstrates the agreement of our model with both measured stall forces and dynein intensities. Sample size $n = 116$ (1 μm phagosomes), 73 (2 μm), 67 (2.8 μm), 46 (4 μm), and 44 (5 μm). Error bars represent the 95% confidence interval estimated by bootstrapping.

cross-sectional area (constant microtubule area density ρ_{MT}). The displaced microtubules bend around the phagosome, depending on their distance r to the phagosome center. Therefore, the interaction length $l_{int}(r)$, on which a microtubule that would cross the phagosome at a given radius r will interact with it, is

$$l_{int}(r) = \frac{D}{2}\theta_{int}(r), \tag{8}$$

with the interaction angle $\theta_{int}(r)$. We expect that only the projection of the force of a motor at a given angle θ on the phagosome contributes to the phagosomal stall force. Therefore, we weighted the interaction length with $\cos(\theta)$ to obtain an effective interaction length $l_{int,eff}$ of motors that contribute to the stall force. Dyneins with a maximal distance to a microtubule of one motor length d_M may interact with this microtubule (Fig. S8 *A*), leading to an effective area of interaction $A_{int,eff}$ of about $2\, l_{int,eff}\, d_M$ on the phagosome. Summing up all the interaction areas for all the displaced microtubules by integrating over the cross-sectional area of the phagosome $A_{\varnothing}$ yields the effective number of all possible dynein-microtubule interactions contributing to the stall force:

$$N_{int,eff}(D) = \rho_{Dyn}\, \rho_{MT} \int_{A_{\varnothing}} A_{int,eff}\, dA. \tag{9}$$

Together with the force of a single dynein F_{SD} and the model offset c_m, we modeled the measured stall forces:

$$F_{stall}(D) = F_{SD} N_{int,eff}(D) + c_m = fD^3 + c_m, \quad (10)$$

with the force density $f = 0.5 F_{SD} \rho_{Dyn} \rho_{MT} d_M$. The constant offset was included because at least one force-producing unit was active during stall-force measurements as a consequence of our measurement method. In addition, to assess the forces predicted by the model with respect to dynein numbers, we can write it to directly include the measured dynein intensity on phagosomes I_{dyn} by rewriting the dynein surface density ρ_{Dyn}:

$$\rho_{Dyn} = \frac{N_{Dyn}}{\pi D^2} = \frac{I_{dyn}}{\pi I_{singledyn} D^2}. \quad (11)$$

To convert from the dynein number N_{Dyn} to the dynein intensity I_{dyn} on phagosomes in our assay, we used the fluorescence intensity of a single dynein complex in our assay, $I_{singledyn}$. Taken together, this yields

$$F_{stall} = f' I_{phago} D + c_m, \quad (12)$$

with the modified force density $f' = \frac{0.5 F_{SD} \rho_{MT} d_M}{\pi I_{singledyn}} = \frac{f}{\pi \rho_{Dyn} I_{singledyn}}$.

This model fits the experimentally determined stall forces very well and provides an explanation for how the dynein numbers increasing with D^2 generate stall forces increasing with D^3 (Eq. 10; Fig. 4 *D*). In addition, we plotted the modeled stall forces against the dynein intensities measured on the isolated phagosomes (Eq. 12; Fig. 4 *E*). There is a clear agreement of the measured forces and dynein intensities with our model.

We measured a force density f of (1.3 ± 0.1) pN μm^{-3} and an offset c_m of (7 ± 2) pN. From the fitted force density f, we estimated the cytoplasmic microtubule density ρ_{MT} of (11 ± 8) microtubules μm^{-2} for a dynein surface density ρ_{Dyn} of (3 ± 1) dyneins μm^{-2}, a single dynein stall force F_{SD} of 1–4 pN (11–14,20), and a dynein length d_M of 65 nm (71).

DISCUSSION

Understanding how large groups of molecular motors interact with each other and the cytoskeleton to produce large collective forces is of major biological and biomedical importance. Here, we demonstrated that the transport of large organelles is coordinated by up to hundreds of dyneins that cluster into many teams and likely interact with multiple microtubules. The involvement of such large numbers of dyneins was measured in two independent ways: first, the dynein-inhibition experiments showed that this motor is mainly responsible for the retrograde transport. The combination with the direct measurement of the transport forces of up to 160 pN (95% confidence interval: 150–180 pN) shows that such large numbers of motors need to be involved. Second, the motor numbers of the phagosomes were directly quantified by stepwise photobleaching.

Clustering of dyneins into teams may be caused by the enrichment of dynein on lipid rafts (28) and by dynamic clustering under load (29). This clustering of dynein may be a general mechanism of dynein's collective action (15,28,29), as, within a single dynein team, the load is distributed between individual motors via the elastic lipid membrane of the organelles (28). The ability of dyneins to adjust their step length and frequency of backstepping under a resisting load (12,23) permits leading dyneins to slow down while trailing dyneins can catch up, allowing them to combine their individual forces (11,28). Additionally, their high binding affinity to microtubules allows the dyneins to stay attached even at high loads (11,24–26).

We hypothesize that similar mechanisms may mediate the collective force generation of multiple dynein teams. Forces may be transmitted between dynein teams via the phagosomal membrane. Leading dynein teams may thus experience an increased resisting force, on average slowing down the motion of individual dyneins in such a team. In contrast, trailing dynein teams may bear less load, allowing the individual dyneins in these teams to increase their step length and catch up. The high binding affinity of dyneins to microtubules may prevent dynein teams that momentarily experience very high loads from falling off. In this way, the dynein teams may evenly share the load between them and produce high forces collectively. This mechanism of collective action of dynein teams may also affect their net transport velocity: to enable trailing dynein teams to catch up, leading dynein teams need to slow down or halt their motion, which may, on average, decrease the cargo velocity. Consistently, we noticed that the average free-transport velocity of the intracellular cargoes was about 25 nm s^{-1}, which is in agreement with previous studies on large intracellular cargoes with diameters of a few micrometers (38) and much lower than the velocity of 500–1000 nm s^{-1} that was measured for dynein in vitro (14,23,49). Additionally, individual dyneins that were studied in vitro with no or small cargoes experience a much smaller drag compared to the groups of dyneins on phagosomes in vivo and can therefore move faster. This is evident from in vitro measurements of the retrograde transport velocity of purified phagosomes and beads decorated with dyneins, which move with velocities of ca. 200–1200 nm s^{-1} (3,14,34). However, these experiments are not directly comparable to our measurements, since the reconstituted organelles were placed on top of straight microtubules on a glass coverslip. In this situation, as suggested by the model of Rai et al. (28), only one team of dyneins can be active due to the limited area of interaction with the microtubules, and the load is not shared between multiple dynein teams. Furthermore, for small phagosomes (1-μm diameter) transient velocities of more than 100 nm s^{-1} have been documented in vivo (72) on timescales that are smaller than the ones investigated here.

Because of the ability of dynein teams to produce forces collectively and their size-dependent interactions with the microtubule cytoskeleton, we observed that the forces during retrograde organelle transport scale with approximately D^3. In the power-law fit of the stall forces, we included an offset c (experimental data) or c_m (model), since at least one force-producing unit must be active to observe retrograde organelle transport. Likely, this minimal force-producing unit consists of two dyneins due to the pairwise recruitment of dynein to the phagosomes via the Rab7/RILP complex (11,73,74). Therefore, an offset of 2–8 pN is expected (11–14). We observed an offset of 6–7 pN, consistent with reports of a stall force of 3–4 pN for a single dynein, indicating that cargo adaptors were involved in the retrograde transport (14,18,75). Our observation of a minimal transport force agrees with earlier reports about organelle stall forces in vivo. For even smaller phagosomes (diameter of 0.74 μm) stall forces of 6–10 pN were measured (11), and, for lipid droplets (diameter of 0.4–0.6 μm), average stall forces of 2–10 pN (75,76) were found. However, the stall forces were broadly distributed, with individual forces as low as 1–2 pN. These low forces measured in optical-tweezer experiments likely were related to the motors' processivity, if the stall force is not reached before the motors detach (20). These observations together with the results presented in this study may potentially suggest that the scaling behavior of retrograde transport forces with cargo size is divided into distinct regimes: for small cargoes ($\lesssim$1 μm), the observed stall forces seemed to be independent of cargo size (11,75,76). In this regime, dyneins in only one team may interact with one microtubule (28) and combine their forces linearly, modulated by force-dependent motor detachment (70). Steric interactions of dyneins within such a dynein team may potentially limit the collective force production, leading to a size-independent stall force. A similar effect has been observed for myosins and kinesins, where only a limited number of motors can simultaneously engage with an actin filament or microtubule (77,78).

In contrast, for cargoes with diameters of a few micrometers, microtubule displacement and bending will play a role, as suggested by our model and data. In this regime, the transport force also linearly depends on the number of active dyneins. However, this number additionally increases with the increasing microtubule density on the phagosomes' surface due to their displacement and bending. This effectively leads to the observed scaling exponent of $F \sim D^3$.

We found that the transport forces decreased by 50% after a 50% reduction of the expression level of dynein and after a 50% reduction of the ATPase activity of dynein. These results indicate that dynein is the main driver for the observed transport processes. Inhibiting the expression or activity of dynein may affect kinesin's activity as well, which is known as the "paradox of co-dependence" (79,80). How groups of opposing molecular motors coordinate their movement to drive bidirectional transport was investigated by numerous in vitro and in vivo studies (11,21,28,30,32–34,80,81). Consistent with our observations, some of these studies reported that kinesins did not functionally interfere with dynein during retrograde runs (21,30,33,81).

Additionally, we considered the possibility that other contributions to the observed transport forces might originate from the elastic deformation of the cell cortex by the phagosomes. We found that these elastic forces scale linearly with the phagosome diameter D and can therefore not explain the observed D^3 scaling of the retrograde transport forces. Our findings show, in agreement with previous results (67,68), that these elastic forces start to dissipate on timescales longer than $\sim$10 s. Since in our measurements the time between reaching the maximal magnetic force and the onset of retrograde transport is much longer than this time, potential elastic deformations of the cell cortex by the phagosomes likely did not contribute to the measured stall forces. Taken together, this shows that dynein is predominantly responsible for the retrograde transport.

There is plenty of evidence for the collective force generation of dyneins, which can work together in large teams with more than 10 individual motors (11,28,32). Our study further supports these observations of dyneins' collective action, suggesting that multiple dynein teams work together to produce very large collective forces of up to 300 pN, indicating the activity of $\sim$100 motors. However, in the case of kinesins, the picture is less clear. There are several theoretical studies demonstrating that multiple kinesins can, in principle, combine their forces (82,83). These studies are backed by experimental evidence, which shows that, for organelles such as latex bead compartments and lipid droplets, 2–3 kinesins may combine their forces to overcome the collective force of $\sim$10 dyneins (32,84). In intraflagellar trains, average anterograde transport forces of 21 pN were observed, indicating the collective action of more than four kinesins (31). Moreover, in microtubule gliding assays, much higher forces were observed, indicating the combined action of many kinesins (85). However, microtubule gliding assays and intraflagellar transport are unique systems that may not be directly comparable to the transport of organelles in the cytoplasm, which was studied here. We are not aware of any evidence observing the cooperation of many ($\sim$10–100) kinesins during intracellular organelle transport, as was observed for dyneins.

In the light of these studies, it has to be considered that kinesin's mechanochemistry differs from that of dynein, which makes it seem unlikely that large numbers of kinesins can work collectively in teams in the same way as dyneins do. The detachment rate of dynein may decrease in the superstall regime (catch bond) (11,86), remain constant, or slightly increase under load (ideal bond, slip-ideal bond) (25,26,87), depending on the design of the study. Furthermore, the tenacity of dynein motor teams is proportional to the motor copy number in the sub- and superstall regime

(11). In contrast to dynein, kinesin's detachment rate increases in the superstall regime with increasing load (slip bond) (86,88,89). Additionally, neither kinesin's processivity nor its velocity increase with the motor number (84), and the load is usually not shared between multiple kinesins (90). Consequently, the tenacity of kinesins does not increase with increasing motor number (11). However, it is important to note that the mechanochemical properties of molecular motors measured in vitro can be affected by factors like the assay geometry and the stiffness of the optical trap used in the experiments (20,91). To fully understand how dynein and kinesin combine their forces, more detailed studies are needed to connect their individual mechanochemical characteristics with their ability to produce forces collectively.

Overall, the ability of dyneins to produce forces collectively may potentially restrict the ability of kinesins to oppose multiple teams with more than 100 dyneins that drive the retrograde transport of large organelles (23,28,32,33,92). In the case of small organelles, however, we show that only a few dyneins are active, generating forces in the order of 10 pN, which can be balanced by 2–3 kinesins (32,93,94). This may explain why large 3-μm phagosomes were transported very persistently toward the perinuclear region, whereas small 1-μm phagosomes engaged in a bidirectional, tug-of-war-like motion with frequent phases of persistent anterograde transport (38). Similarly, in fibroblasts, it was observed that larger latex-bead compartments were located close to the nucleus after intracellular transport, whereas smaller particles remained in the cell periphery (95). These findings agree with the hypothesis of Rai et al. (28), which suggests that the clustering of dyneins on late phagosomes enhances their collective force generation, allowing them to overcome the anterograde forces of 2–3 kinesins and enable more persistent transport toward the perinuclear region.

Taken together, these results seem to suggest a size-dependent mechanism of intracellular cargo transport in which dyneins, due to their strong ability to produce large forces collectively, can outcompete kinesins on larger organelles, leading to more persistent and less bidirectional retrograde transport. In this study, we investigated this phenomenon for phagosomal transport, since phagocytosis enables the reliable generation of organelles of a very defined size and shape (96). However, not all cellular cargoes have the same properties as the phagosomes used in this study. For example, softer organelles might be subjected to deformations due to stresses from the microtubules and large forces applied by motor teams (97), unlike our phagosomes. Nevertheless, the mechanism we propose here might be applicable to a wider variety of organelles, because it only depends on basic geometric assumptions (constant motor density, displacement and bending of microtubules). Size-dependent interactions of organelles with microtubules possibly contribute to the transport of a range of organelles, as even membrane-less organelles displace the microtubule cytoskeleton in a size-dependent manner (98). A possible application outside of phagocytosis might be the intracellular positioning of nuclei, which also requires the collective action of large numbers of dyneins and kinesins (5,36). This size-dependent regulation of bidirectional cargo transport may complement biochemical mechanisms, which include, for example, cues embedded in the microtubule tracks, such as microtubule-associated proteins, posttranslational microtubule modifications, and microtubule bundling (99,100), or the selective activation of individual motor types, which is linked to the coordination of different cargo adaptors (100).

Our results together with the theoretical model also highlight the role of the microtubule cytoskeleton for the generation of large collective forces. From our model, we derived a prediction for the cytoplasmic microtubule density ρ_{MT} of (11 $\pm$ 8) microtubules μm^{-2}. This value is well within the range of previous reports, underlining the validity of our model. In neutrophils, a cytoplasmic microtubule density of 1 microtubule μm^{-2} was observed (101), in neuron dendrites 50–70 microtubules μm^{-2} were found (102,103), and in axons up to 150 microtubules μm^{-2} (104). There is a remarkable coincidence between the microtubule density and the range of directed organelle transport: in neutrophils and macrophages, distances of only 1–10 μm need to be covered, in dendrites 10–100 μm (105), and in axons 1 mm to 1 m (106). Our model suggests that the number of active dynein teams depends on the microtubule density, indicating that the cytoplasmic microtubule density may be an important factor to regulate the robustness and persistence of intracellular organelle transport.

CONCLUSIONS

The experimental results and theoretical model we present in this study suggest that dynein's inherent ability to generate forces collectively and its interactions with the microtubule cytoskeleton may both be important to drive fast intracellular transport of organelles of different sizes. Since processes such as the degradation of pathogens by phagocytes or the clearance of particulate contaminants like microplastics rely on the efficient transport of organelles spanning orders of magnitude in size, these findings are relevant not only for fundamental cell biology but also from a biomedical perspective.

DATA AND CODE AVAILABILITY

All data needed to evaluate the conclusions in the paper are present in the paper and/or the supporting material. All code used in the evaluation of the data and additional raw data are available from the corresponding author upon request.

ACKNOWLEDGMENTS

We would like to acknowledge and honor Erich Sackmann, whose pioneering contributions to cell biophysics and magnetic force measurement techniques have greatly influenced our work. We thank Thomas Scheibel and Hendrik Bargel for their support with the TIRF setup and Matthias Weiss and Konstantin Speckner for their assistance with the confocal images of the microtubules. This work received funding from the Deutsche Forschungsgemeinschaft (DFG, German Research Foundation)—project number 391977956—SFB 1357 and DFG GZ: INST 91/367-1 FUGG. S.W. and D.E.G. were supported by the Elite Network of Bavaria (Study Program Biological Physics). S.W., D.E.G., W.G., and J.R. were supported by the University of Bayreuth Graduate School.

AUTHOR CONTRIBUTIONS

H.K., A.G.H., and S.W. designed the research. S.W., C.S., D.E.G., and M. M.K. performed the experiments and evaluated the data with support from A.R.C. and J.R. H.K. and S.W. developed the theoretical model. W.G. developed and implemented the setup and procedures for the magnetic force measurements. S.W. wrote the manuscript. All authors discussed the results and revised the manuscript.

DECLARATION OF INTERESTS

The authors declare no competing interests.

SUPPORTING MATERIAL

Supporting material can be found online at https://doi.org/10.1016/j.bpj.2025.09.012.

REFERENCES

1. Reck-Peterson, S. L., W. B. Redwine, …, A. P. Carter. 2018. The cytoplasmic dynein transport machinery and its many cargoes. *Nat. Rev. Mol. Cell Biol.* 19:382–398.
2. Aniento, F., N. Emans, …, J. Gruenberg. 1993. Cytoplasmic dynein-dependent vesicular transport from early to late endosomes. *J. Cell Biol.* 123:1373–1387.
3. Blocker, A., F. F. Severin, …, G. Griffiths. 1997. Molecular Requirements for Bi-directional Movement of Phagosomes Along Microtubules. *J. Cell Biol.* 137:113–129.
4. Schnapp, B. J., and T. S. Reese. 1989. Dynein is the motor for retrograde axonal transport of organelles. *Proc. Natl. Acad. Sci. USA.* 86:1548–1552.
5. Tanaka, T., F. F. Serneo, …, J. G. Gleeson. 2004. Lis1 and doublecortin function with dynein to mediate coupling of the nucleus to the centrosome in neuronal migration. *J. Cell Biol.* 165:709–721.
6. Harada, A., Y. Takei, …, N. Hirokawa. 1998. Golgi Vesiculation and Lysosome Dispersion in Cells Lacking Cytoplasmic Dynein. *J. Cell Biol.* 141:51–59.
7. Varadi, A., L. I. Johnson-Cadwell, …, G. A. Rutter. 2004. Cytoplasmic dynein regulates the subcellular distribution of mitochondria by controlling the recruitment of the fission factor dynamin-related protein-1. *J. Cell Sci.* 117:4389–4400.
8. Lipka, J., M. Kuijpers, …, C. C. Hoogenraad. 2013. Mutations in cytoplasmic dynein and its regulators cause malformations of cortical development and neurodegenerative diseases. *Biochem. Soc. Trans.* 41:1605–1612.
9. Marzo, M. G., J. M. Griswold, …, S. M. Markus. 2019. Molecular basis for dyneinopathies reveals insight into dynein regulation and dysfunction. *eLife.* 8:e47246.
10. Canty, J. T., R. Tan, …, A. Yildiz. 2021. Structure and Mechanics of Dynein Motors. *Annu. Rev. Biophys.* 50:549–574.
11. Rai, A. K., A. Rai, …, R. Mallik. 2013. Molecular Adaptations Allow Dynein to Generate Large Collective Forces inside Cells. *Cell.* 152:172–182.
12. Mallik, R., B. C. Carter, …, S. P. Gross. 2004. Cytoplasmic dynein functions as a gear in response to load. *Nature.* 427:649–652.
13. Belyy, V., N. L. Hendel, …, A. Yildiz. 2014. Cytoplasmic dynein transports cargos via load-sharing between the heads. *Nat. Commun.* 5:5544.
14. Belyy, V., M. A. Schlager, …, A. Yildiz. 2016. The mammalian dynein–dynactin complex is a strong opponent to kinesin in a tug-of-war competition. *Nat. Cell Biol.* 18:1018–1024.
15. Torisawa, T., M. Ichikawa, …, K. Furuta. 2014. Autoinhibition and cooperative activation mechanisms of cytoplasmic dynein. *Nat. Cell Biol.* 16:1118–1124.
16. Zhang, K., H. E. Foster, …, A. P. Carter. 2017. Cryo-EM Reveals How Human Cytoplasmic Dynein Is Auto-inhibited and Activated. *Cell.* 169:1303–1314.e18.
17. Trokter, M., N. Mücke, and T. Surrey. 2012. Reconstitution of the human cytoplasmic dynein complex. *Proc. Natl. Acad. Sci. USA.* 109:20895–20900.
18. Ross, J. L., K. Wallace, …, E. L. F. Holzbaur. 2006. Processive bidirectional motion of dynein–dynactin complexes in vitro. *Nat. Cell Biol.* 8:562–570.
19. Mallik, R., D. Petrov, …, S. P. Gross. 2005. Building Complexity: An In Vitro Study of Cytoplasmic Dynein with In Vivo Implications. *Curr. Biol.* 15:2075–2085.
20. Brenner, S., F. Berger, …, A. Gennerich. 2020. Force production of human cytoplasmic dynein is limited by its processivity. *Sci. Adv.* 6:eaaz4295.
21. Derr, N. D., B. S. Goodman, …, S. L. Reck-Peterson. 2012. Tug-of-War in Motor Protein Ensembles Revealed with a Programmable DNA Origami Scaffold. *Science.* 338:662–665.
22. Urnavicius, L., C. K. Lau, …, A. P. Carter. 2018. Cryo-EM shows how dynactin recruits two dyneins for faster movement. *Nature.* 554:202–206.
23. Elshenawy, M. M., J. T. Canty, …, A. Yildiz. 2019. Cargo adaptors regulate stepping and force generation of mammalian dynein–dynactin. *Nat. Chem. Biol.* 15:1093–1101.
24. Cleary, F. B., M. A. Dewitt, …, A. Yildiz. 2014. Tension on the linker gates the ATP-dependent release of dynein from microtubules. *Nat. Commun.* 5:4587.
25. Nicholas, M. P., F. Berger, …, A. Gennerich. 2015. Cytoplasmic dynein regulates its attachment to microtubules via nucleotide state-switched mechanosensing at multiple AAA domains. *Proc. Natl. Acad. Sci. USA.* 112:6371–6376.
26. Ezber, Y., V. Belyy, …, A. Yildiz. 2020. Dynein harnesses active fluctuations of microtubules for faster movement. *Nat. Phys.* 16:312–316.
27. Ori-McKenney, K. M., J. Xu, …, R. B. Vallee. 2010. A cytoplasmic dynein tail mutation impairs motor processivity. *Nat. Cell Biol.* 12:1228–1234.
28. Rai, A., D. Pathak, …, R. Mallik. 2016. Dynein Clusters into Lipid Microdomains on Phagosomes to Drive Rapid Transport toward Lysosomes. *Cell.* 164:722–734.
29. Chowdary, P. D., L. Kaplan, …, B. Cui. 2018. Dynamic Clustering of Dyneins on Axonal Endosomes: Evidence from High-Speed Darkfield Imaging. *Biophys. J.* 115:230–241.
30. Laib, J. A., J. A. Marin, …, W. H. Guilford. 2009. The reciprocal coordination and mechanics of molecular motors in living cells. *Proc. Natl. Acad. Sci. USA.* 106:3190–3195.
31. Shih, S. M., B. D. Engel, …, A. Yildiz. 2013. Intraflagellar transport drives flagellar surface motility. *eLife.* 2:e00744.

32. Hendricks, A. G., E. L. F. Holzbaur, and Y. E. Goldman. 2012. Force measurements on cargoes in living cells reveal collective dynamics of microtubule motors. *Proc. Natl. Acad. Sci. USA.* 109:18447–18452.
33. Blehm, B. H., T. A. Schroer, …, P. R. Selvin. 2013. In vivo optical trapping indicates kinesin's stall force is reduced by dynein during intracellular transport. *Proc. Natl. Acad. Sci. USA.* 110:3381–3386.
34. Chaudhary, A. R., F. Berger, …, A. G. Hendricks. 2018. Tau directs intracellular trafficking by regulating the forces exerted by kinesin and dynein teams. *Traffic.* 19:111–121.
35. Sims, P. A., and X. S. Xie. 2009. Probing Dynein and Kinesin Stepping with Mechanical Manipulation in a Living Cell. *ChemPhysChem.* 10:1511–1516.
36. Tanenbaum, M. E., A. Akhmanova, and R. H. Medema. 2011. Bi-directional transport of the nucleus by dynein and kinesin-1. *Commun. Integr. Biol.* 4:21–25.
37. Shami, G. J., D. Cheng, …, F. Braet. 2021. Three-dimensional ultrastructure of giant mitochondria in human non-alcoholic fatty liver disease. *Sci. Rep.* 11:3319.
38. Keller, S., K. Berghoff, and H. Kress. 2017. Phagosomal transport depends strongly on phagosome size. *Sci. Rep.* 7:17068.
39. Vieira, O. V., R. J. Botelho, and S. Grinstein. 2002. Phagosome maturation: aging gracefully. *Biochem. J.* 366:689–704.
40. Yu, Y., Z. Zhang, …, Y. Yu. 2022. Kinetics of phagosome maturation is coupled to their intracellular motility. *Commun. Biol.* 5:1014.
41. Aderem, A., and D. M. Underhill. 1999. Mechanisms of phagocytosis in macrophages. *Annu. Rev. Immunol.* 17:593–623.
42. Alhede, M., M. Lorenz, …, T. Bjarnsholt. 2020. Bacterial aggregate size determines phagocytosis efficiency of polymorphonuclear leukocytes. *Med. Microbiol. Immunol.* 209:669–680.
43. Wieland, S., A. F. R. M. Ramsperger, …, H. Kress. 2024. Nominally identical microplastic models differ greatly in their particle-cell interactions. *Nat. Commun.* 15:922.
44. Koelmans, A. A., P. E. Redondo-Hasselerharm, …, M. Kooi. 2022. Risk assessment of microplastic particles. *Nat. Rev. Mater.* 7:138–152.
45. Seaton, A., W. MacNee, …, D. Godden. 1995. Particulate air pollution and acute health effects. *Lancet.* 345:176–178.
46. Hiramoto, Y. 1969. Mechanical properties of the protoplasm of the sea urchin egg. *Exp. Cell Res.* 56:201–218.
47. Bausch, A. R., W. Möller, and E. Sackmann. 1999. Measurement of Local Viscoelasticity and Forces in Living Cells by Magnetic Tweezers. *Biophys. J.* 76:573–579.
48. Xie, J., J. Najafi, …, N. Minc. 2022. Contribution of cytoplasm viscoelastic properties to mitotic spindle positioning. *Proc. Natl. Acad. Sci. USA.* 119:e2115593119.
49. Elshenawy, M. M., E. Kusakci, …, A. Yildiz. 2020. Lis1 activates dynein motility by modulating its pairing with dynactin. *Nat. Cell Biol.* 22:570–578.
50. Eisentraut, M., A. Sabri, and H. Kress. 2023. The spatial resolution limit of phagocytosis. *Biophys. J.* 122:868–879.
51. Zahn, C., S. Keller, …, H. Kress. 2017. Measurement of the magnetic moment of single Magnetospirillum gryphiswaldense cells by magnetic tweezers. *Sci. Rep.* 7:3558.
52. Berghoff, K., W. Gross, …, H. Kress. 2021. Using blinking optical tweezers to study cell rheology during initial cell-particle contact. *Biophys. J.* 120:3527–3537.
53. Lewis, J. P. 1995. Fast template matching. *In* Vision Interface Canadian Image Processing. and Pattern Recognition Society, pp. 15–19.
54. Loy, G., and A. Zelinsky. 2003. Fast radial symmetry for detecting points of interest. *IEEE Trans. Pattern Anal. Mach. Intell.* 25:959–973.
55. Aumeier, C., L. Schaedel, …, M. Théry. 2016. Self-repair promotes microtubule rescue. *Nat. Cell Biol.* 18:1054–1064.
56. Schaedel, L., K. John, …, M. Théry. 2015. Microtubules self-repair in response to mechanical stress. *Nat. Mater.* 14:1156–1163.
57. Gazzola, M., A. Schaeffer, …, M. Théry. 2023. Microtubules self-repair in living cells. *Curr. Biol.* 33:122–133.e4.
58. Schulze, E., and M. Kirschner. 1987. Dynamic and stable populations of microtubules in cells. *J. Cell Biol.* 104:277–288.
59. Sammak, P. J., and G. G. Borisy. 1988. Direct observation of microtubule dynamics in living cells. *Nature.* 332:724–726.
60. Firestone, A. J., J. S. Weinger, …, J. K. Chen. 2012. Small-molecule inhibitors of the AAA+ ATPase motor cytoplasmic dynein. *Nature.* 484:125–129.
61. Hendricks, A. G., Y. E. Goldman, and E. L. F. Holzbaur. 2014. Reconstituting the Motility of Isolated Intracellular Cargoes. *In* Methods in Enzymology. R. D. Vale, ed Academic Press, pp. 249–262.
62. Chen, Y., N. C. Deffenbaugh, …, W. O. Hancock. 2014. Molecular counting by photobleaching in protein complexes with many subunits: best practices and application to the cellulose synthesis complex. *Mol. Biol. Cell.* 25:3630–3642.
63. Stadler, L., K. Speckner, and M. Weiss. 2018. Diffusion of Exit Sites on the Endoplasmic Reticulum: A Random Walk on a Shivering Backbone. *Biophys. J.* 115:1552–1560.
64. Lam, J., M. Herant, …, V. Heinrich. 2009. Baseline Mechanical Characterization of J774 Macrophages. *Biophys. J.* 96:248–254.
65. Beaudet, D., C. L. Berger, and A. G. Hendricks. 2024. The types and numbers of kinesins and dyneins transporting endocytic cargoes modulate their motility and response to tau. *J. Biol. Chem.* 300:107323.
66. Hu, J., S. Jafari, …, M. Guo. 2017. Size- and speed-dependent mechanical behavior in living mammalian cytoplasm. *Proc. Natl. Acad. Sci. USA.* 114:9529–9534.
67. Salbreux, G., G. Charras, and E. Paluch. 2012. Actin cortex mechanics and cellular morphogenesis. *Trends Cell Biol.* 22:536–545.
68. Fritzsche, M., A. Lewalle, …, G. Charras. 2013. Analysis of turnover dynamics of the submembranous actin cortex. *Mol. Biol. Cell.* 24:757–767.
69. Hurst, S., B. E. Vos, …, T. Betz. 2021. Intracellular softening and increased viscoelastic fluidity during division. *Nat. Phys.* 17:1270–1276.
70. Uçar, M. C., and R. Lipowsky. 2020. Collective Force Generation by Molecular Motors Is Determined by Strain-Induced Unbinding. *Nano Lett.* 20:669–676.
71. Grotjahn, D. A., S. Chowdhury, …, G. C. Lander. 2018. Cryo-electron tomography reveals that dynactin recruits a team of dyneins for processive motility. *Nat. Struct. Mol. Biol.* 25:203–207.
72. Al-Haddad, A., M. A. Shonn, …, S. A. Kuznetsov. 2001. Myosin Va bound to phagosomes binds to F-actin and delays microtubule-dependent motility. *Mol. Biol. Cell.* 12:2742–2755.
73. Johansson, M., N. Rocha, …, J. Neefjes. 2007. Activation of endosomal dynein motors by stepwise assembly of Rab7–RILP–p150Glued, ORP1L, and the receptor βlll spectrin. *J. Cell Biol.* 176:459–471.
74. Wu, M., T. Wang, …, H. Song. 2005. Structural basis for recruitment of RILP by small GTPase Rab7. *EMBO J.* 24:1491–1501.
75. Reddy, B. J. N., M. Mattson, …, S. P. Gross. 2016. Load-induced enhancement of Dynein force production by LIS1–NudE in vivo and in vitro. *Nat. Commun.* 7:12259.
76. Leidel, C., R. A. Longoria, …, G. T. Shubeita. 2012. Measuring Molecular Motor Forces In Vivo: Implications for Tug-of-War Models of Bidirectional Transport. *Biophys. J.* 103:492–500.
77. Lombardo, A. T., S. R. Nelson, …, D. M. Warshaw. 2017. Myosin Va molecular motors manoeuvre liposome cargo through suspended actin filament intersections in vitro. *Nat. Commun.* 8:15692.
78. Bensel, B. M., S. B. Previs, …, D. M. Warshaw. 2024. Kinesin-1-transported liposomes prefer to go straight in 3D microtubule intersections by a mechanism shared by other molecular motors. *Proc. Natl. Acad. Sci. USA.* 121:e2407330121.
79. Hancock, W. O. 2014. Bidirectional cargo transport: moving beyond tug of war. *Nat. Rev. Mol. Cell Biol.* 15:615–628.

80. Ali, F. A., A. J. Zwetsloot, …, A. Straube. 2023. KIF1C activates and extends dynein movement through the FHF cargo adaptor. Preprint at bioRxiv. https://doi.org/10.1101/2023.10.26.564242.
81. D'Souza, A. I., R. Grover, …, S. Diez. 2023. Vesicles driven by dynein and kinesin exhibit directional reversals without regulators. *Nat. Commun.* 14:7532.
82. Khataee, H., and J. Howard. 2019. Force Generated by Two Kinesin Motors Depends on the Load Direction and Intermolecular Coupling. *Phys. Rev. Lett.* 122:188101.
83. Hendricks, A. G., B. I. Epureanu, and E. Meyhöfer. 2009. Collective dynamics of kinesin. *Phys. Rev. E.* 79:031929.
84. Shubeita, G. T., S. L. Tran, …, S. P. Gross. 2008. Consequences of Motor Copy Number on the Intracellular Transport of Kinesin-1-Driven Lipid Droplets. *Cell.* 135:1098–1107.
85. Bormuth, V., A. Jannasch, …, E. Schäffer. 2008. Optical trapping of coated microspheres. *Opt. Express.* 16:13831–13844.
86. Kunwar, A., S. K. Tripathy, …, S. P. Gross. 2011. Mechanical stochastic tug-of-war models cannot explain bidirectional lipid-droplet transport. *Proc. Natl. Acad. Sci. USA.* 108:18960–18965.
87. Gicking, A. M., T. C. Ma, …, W. O. Hancock. 2022. Kinesin-1,-2, and-3 motors use family-specific mechanochemical strategies to effectively compete with dynein during bidirectional transport. *eLife.* 11:e82228.
88. Ohashi, K. G., L. Han, …, W. O. Hancock. 2019. Load-dependent detachment kinetics plays a key role in bidirectional cargo transport by kinesin and dynein. *Traffic.* 20:284–294.
89. Arpağ, G., S. R. Norris, …, E. Tüzel. 2019. Motor Dynamics Underlying Cargo Transport by Pairs of Kinesin-1 and Kinesin-3 Motors. *Biophys. J.* 116:1115–1126.
90. Jamison, D. K., J. W. Driver, …, M. R. Diehl. 2010. Two Kinesins Transport Cargo Primarily via the Action of One Motor: Implications for Intracellular Transport. *Biophys. J.* 99:2967–2977.
91. Pyrpassopoulos, S., H. Shuman, and E. M. Ostap. 2020. Modulation of Kinesin's Load-Bearing Capacity by Force Geometry and the Microtubule Track. *Biophys. J.* 118:243–253.
92. Jamison, D. K., J. W. Driver, and M. R. Diehl. 2012. Cooperative Responses of Multiple Kinesins to Variable and Constant Loads. *J. Biol. Chem.* 287:3357–3365.
93. Carter, N. J., and R. A. Cross. 2005. Mechanics of the kinesin step. *Nature.* 435:308–312.
94. Visscher, K., M. J. Schnitzer, and S. M. Block. 1999. Single kinesin molecules studied with a molecular force clamp. *Nature.* 400:184–189.
95. Kodali, V. K., W. Roos, …, J. E. Curtis. 2007. Cell-assisted assembly of colloidal crystallites. *Soft Matter.* 3:337–348.
96. Champion, J. A., and S. Mitragotri. 2006. Role of target geometry in phagocytosis. *Proc. Natl. Acad. Sci. USA.* 103:4930–4934.
97. McIntosh, B. B., S. Pyrpassopoulos, …, E. M. Ostap. 2018. Opposing Kinesin and Myosin-I Motors Drive Membrane Deformation and Tubulation along Engineered Cytoskeletal Networks. *Curr. Biol.* 28:236–248.e5.
98. Böddeker, T. J., K. A. Rosowski, …, E. R. Dufresne. 2022. Non-specific adhesive forces between filaments and membraneless organelles. *Nat. Phys.* 18:571–578.
99. Balabanian, L., A. R. Chaudhary, and A. G. Hendricks. 2018. Traffic control inside the cell: microtubule-based regulation of cargo transport. *Biochemist.* 40:14–17.
100. Cason, S. E., and E. L. F. Holzbaur. 2022. Selective motor activation in organelle transport along axons. *Nat. Rev. Mol. Cell Biol.* 23:699–714.
101. Rich, A. M., and S. T. Hoffstein. 1981. Inverse correlation between neutrophil microtubule numbers and enhanced random migration. *J. Cell Sci.* 48:181–191.
102. Katrukha, E. A., D. Jurriens, …, L. C. Kapitein. 2021. Quantitative mapping of dense microtubule arrays in mammalian neurons. *eLife.* 10:e67925.
103. Kubota, Y., F. Karube, …, Y. Kawaguchi. 2011. Conserved properties of dendritic trees in four cortical interneuron subtypes. *Sci. Rep.* 1:89.
104. White, L. A., P. W. Baas, and S. R. Heidemann. 1987. Microtubule stability in severed axons. *J. Neurocytol.* 16:775–784.
105. Fiala, J. C., J. Spacek, and K. M. Harris. 2007. Dendrite structure. *In* Dendrites. G. Stuart, N. Spruston, and M. Häusser, eds Oxford University Press, pp. 1–41.
106. Fletcher, D. A., and J. A. Theriot. 2004. An introduction to cell motility for the physical scientist. *Phys. Biol.* 1:T1–T10.Supporting References
107. Crocker, J. C., and D. G. Grier. 1996. Methods of digital video microscopy for colloidal studies. *J. Colloid Interface Sci.* 179:298–310.
108. Holmes, K. L., and L. M. Lantz. 2001. Protein labeling with fluorescent probes. *In* Methods in Cell Biology. Z. Darzynkiewicz, H. A. Crissman, and J. P. Robinson, eds Academic Press, pp. 185–204.
109. Wu, K.-L., C. Yu, …, H. Xiao. 2021. Precision Modification of Native Antibodies. *Bioconjug. Chem.* 32:1947–1959.
110. Kollmannsberger, P., and B. Fabry. 2011. Linear and nonlinear rheology of living cells. *Annu. Rev. Mater. Res.* 41:75–97.

Biophysical Journal, Volume *125*

Supplemental information

Many dynein teams collectively generate high forces during the transport of large organelles

Simon Wieland, Christina Steininger, David E. Gitschier, Marius M. Kaiser, Wolfgang Gross, Abdullah R. Chaudhary, Jana Ritschar, Christian Laforsch, Adam G. Hendricks, and Holger Kress

Supporting Information for

Many dynein teams collectively generate high forces during the transport of large organelles

Simon Wieland[1,2], Christina Steininger[1], David E. Gitschier[1], Marius M. Kaiser[1], Wolfgang Gross[1], Abdullah R. Chaudhary[3], Jana Ritschar[4], Christian Laforsch[2], Adam G. Hendricks[3], Holger Kress[1,*]

[1] Biological Physics, University of Bayreuth, Bayreuth, Germany
[2] Animal Ecology I, University of Bayreuth, Bayreuth, Germany
[3] Department of Bioengineering, McGill University, Montreal, Canada
[4] Laboratory of Molecular Parasitology, University of Bayreuth, Bayreuth, Germany

*Corresponding Author: Holger Kress

Email: holger.kress@uni-bayreuth.de

This PDF file includes:

SI Appendix
Figures S1 to S16
Tables S1 to S4
SI References

SI Appendix

Calibration of the magnetic tweezers. We calibrated the force of the magnetic tweezers on the magnetic beads by suspending ca. 1.6×10^6 beads in 1 ml of a 75%/25% (v/v) glycerol-water solution. The viscosity of this solution was calibrated in the temperature range of 20-25°C using an Ostwald viscosimeter. It followed approximately a linear relation

$$\eta(T) = 0.103\ \mathrm{Pa\ s} - 0.0026\ \mathrm{Pa\ s\ °C^{-1}} \times T,$$

with the solution's temperature T in °C. The uncertainty of the viscosity s_η was about 5%. The tip of the magnetic tweezers was immersed in 150 µl of the calibration solution, placed 100 µm above the coverslip and brought into focus. Then, the coil current was turned on and the beads started moving towards the tip of the magnetic tweezers (Figure S9 A). From the bead trajectories, their velocity was determined as previously described (1). Briefly, we detected the particles in each frame with a peak-finding algorithm. Using the tracking algorithm by Crocker and Grier (2), the individual particle positions were linked to trajectories. The instantaneous particle velocity $\boldsymbol{v}$ was then calculated for each particle position along the trajectories over intervals of three frames. Here, only particles in a range of ±40° from the tip axis were considered to ensure radial symmetry of the magnetic gradient field. The tip of the magnetic tweezers was detected by automated image analysis and the shortest distance d between the particle and the tip surface was calculated for each particle position. Using Stokes' law of friction, we obtained the force-distance relationships for the individual particle types:

$$F_{\mathrm{MT}}(d) = 6\pi\, \eta\, R\, v(d)$$

Heuristically, we fitted the force-distance relationship with a power law to obtain the calibration curves:

$$F_{\mathrm{MT}}(d) = \frac{F_0}{\left(\frac{d - r_0}{d_0}\right)^c}$$

The parameter d_0 was set to 25 µm. The fit results for F_0, r_0, and c are shown in Table S2 for all particle types. Three factors contributed to the uncertainty of the calibration curve: velocity uncertainties due to tracking errors, variations in size and magnetic moment of the individual particles, and the uncertainty of the viscosity. Both tracking errors and variations of the beads' size and magnetic moment resulted in variations of $F_{\mathrm{MT}}(d)$. To determine their magnitude, we fitted an exponential function to the absolute values of the differences between the data points and the fitted calibration curve, averaged over intervals of 3 µm in the parameter d:

$$s_{\mathrm{scatter}}(d) = a \exp\left(-\frac{d}{b}\right) + k$$

The fit results for the parameters a, b, and k are shown in Table S2. Together with the uncertainty of the viscosity, the total uncertainty of the calibration curve is

$$s_F(d) = \left[\left(\frac{\partial F(d)}{\partial \eta} \times s_\eta\right)^2 + s_{\mathrm{scatter}}^2(d)\right]^{\frac{1}{2}} = \left[\left(\frac{s_\eta}{\eta} \times F(d)\right)^2 + s_{\mathrm{scatter}}^2(d)\right]^{\frac{1}{2}}$$
$$= \left[\left(0.05 \times F(d)\right)^2 + s_{\mathrm{scatter}}^2(d)\right]^{\frac{1}{2}}.$$

The resulting calibration curves are shown with their respective uncertainties in Figure S9 B-F.

Western blot protocol. After transfection, the medium of the cells was replaced by 100 µl of homogenization buffer (HB, consisting of motility assay buffer (MAB): 10 mM PIPES, 50 mM K-Acetate, 4 mM MgCl2, and 1 mM EGTA; pH 7.0; with 10% sucrose, 1x cOmplete EDTA-free

protease inhibitors (Sigma Aldrich, Darmstadt, Germany), 10 µM ATP, and 30 µM DTT), cells were chilled on ice, scraped from the bottom of the wells, and transferred to a 500 µl Eppendorf tube. The cells were homogenized on ice in an ultrasound bath 3x for 10 s. Then, 20 µl of 6x Laemmli buffer was added, and the lysate was heated to 70°C for 10 min. The lysate was stored at -20°C.
Prior to blotting, the lysates were thawed and heated to 95°C and centrifuged at 17,000 g for 30 s to remove debris. A precast SDS-PAGE gel (10% Mini-PROTEAN® TGX™ Precast Protein Gels, BioRad Laboratories Inc., Hercules, CA) was inserted into the electrophoresis cell (Mini-PROTEAN Tetra cell, BioRad Laboratories Inc., Hercules, CA) and filled with running buffer (0.29% w/v Tris, 1.44% w/v Glycine, 0.1% SDS w/v in MilliQ water). To each pocket of the gel, 20 µl of the lysate were added. In one pocket, 5 µl of a protein ladder (PageRuler Plus Prestained Protein Ladder, Thermo Fisher Scientific, Waltham, MA) were added. Electrophoresis was started for 15 min at 80 V, and was continued at 120 V for approximately 1 h, until the protein ladder was spread out over the whole gel. Before the western blot, the nitrocellulose membrane (Amersham Protran 0.45 µm NC Nitrocellulose Blotting Membrane, GE Healthcare, Chicago, IL), filter paper (Mini Trans-Blot Filter paper, BioRad Laboratories Inc., Hercules, CA), and sponges were soaked thoroughly in the transfer buffer (0.29% w/v Tris, 1.45% w/v Glycine, 20% v/v methanol in MilliQ water). Then, the blot was assembled, inserted into the electrophoresis cell, filled with transfer buffer, and ran at 100 mA overnight at room temperature.
After blotting, the nitrocellulose membrane was washed in PBS. To verify successful blotting, proteins were stained using Ponceau S (Sigma Aldrich, Darmstadt, Germany). The Ponceau dye was then washed out with MilliQ water. To prevent unspecific binding of the antibodies, the membrane was incubated 2x 15 min in PBS-T (PBS with 0.1% Tween-20) with 5% w/v milk powder. In between and after the incubation, the membrane was washed 3x with PBS-T. The primary antibodies (mouse anti dynein intermediate chain: Invitrogen Dynein Monoclonal Antibody (74.1) MA1-070, Thermo Fisher Scientific, Waltham, MA. mouse anti α-tubulin as loading control: alpha-Tubulin (DM1A) Mouse mAB, Cell Signaling Technology, Cambridge, UK) were diluted 1:1000 in PBS-T containing 5% w/v milk powder. The membrane was incubated for 1h at room temperature with the primary antibodies, then washed 3x with PBS-T. The secondary antibody (goat anti mouse, horseradish peroxidase: Immun-Star Goat Anti-Mouse (GAM)-HRP Conjugate #1705047, BioRad Laboratories Inc., Hercules, CA) was diluted 1:1000 in PBS-T containing 5% w/v milk powder and incubated for 1h at room temperature with the membrane. Finally, the membrane was washed 3x with PBS-T and then 3x with PBS. The membrane was then transferred to a transparent plastic bag. 400 µl of the chemiluminescence substrate (Clarity ECL Western Blot Substrate, BioRad Laboratories Inc., Hercules, CA) were prepared and homogeneously distributed over the nitrocellulose membrane. The bag was tightly sealed. The chemiluminescence signal was recorded using a biomolecular imager (ImageQuant LAS 4000, GE Healthcare, Freiburg, Germany).

Dynein immunofluorescence protocol. For the immunofluorescent labeling of dynein on the phagosomes, small flow channels were assembled with the treated coverslips and microscopy slides using Parafilm and vacuum grease. The channels were rinsed 3x with MAB. Then, 15 µl of the isolated phagosomes were suspended in 60 µl of MAB and flown into the flow channel. The phagosomes were left for 1 h to settle on the hydrophobic coverslip. After washing 3× with MAB, 60 µl of 5 µM Pluronic F-127 (Sigma Aldrich, Darmstadt, Germany) was added to block the coverslip for 30 min. After washing 3× with MAB, phagosomes were fixed for 15 min with 4% paraformaldehyde (PFA) and then washed 2x with MAB-T (MAB with 0.1% Tween 20) and 1x with MAB before overnight incubation at 4°C with the primary antibody (Invitrogen Dynein Monoclonal Antibody (74.1) MA1-070, Thermo Fisher Scientific Inc., Waltham, MA). The next day, samples were washed 2x with MAB-T and 1x with MAB and incubated for 45 min at RT with the secondary antibody (Invitrogen Goat Anti-Mouse IgG, DyLight 488 conjugated, Thermo Fisher Scientific Inc., Waltham, MA). After washing 2x with MAB-T and 1x with MAB, the flow chamber was sealed with vacuum grease. Samples were imaged on the same day. To ensure specificity of the primary antibody, western blots of the total cell lysate and isolated phagosomes were performed. Furthermore, suitable control samples were always prepared (phagosomes stained without primary antibody to ensure specificity of the secondary antibody; unphagocytosed beads with primary and secondary antibody and only secondary antibody). There was a slight fluorescent signal from the unphagocytosed beads stained with primary and secondary antibody, indicating a weak unspecific

binding of the primary antibody to the carboxylate beads. There was no notable unspecific binding of the secondary antibody.

Replication of the dynein immunofluorescence assay. To validate our dynein immunofluorescence assay, we completely replicated our experiments by producing a new batch of isolated phagosomes and labeled dynein with different types of primary and secondary antibodies. Additionally, we labeled kinesin-2.

J774 cells were grown as described above in 10 cm cell culture dishes until they reached 80% confluency. Carboxylated beads (D = 2 µm, 4 µm, 5 µm) were washed 3× in PBS, and 100 µl of each stock solution was suspended in 10 ml of D10-, with 2.5 ml added per dish (four dishes per bead size). Cells were incubated for 60 min to enable phagocytosis, and subsequently the phagosomes were isolated and purified as described above. However, instead of homogenizing the cells with a syringe, we used a dounce homogenizer (VWR).

For the immunofluorescence assay, we followed the same procedure as before but used a different set of antibodies: Dynein HC Antibody, Santa Cruz, Catalogue Number sc-9115 (primary, 1:500 dilution, incubated at 4°C overnight), and Goat Anti-Rabbit IgG – Atto 488, Sigma Aldrich, Catalogue Number 18772 (secondary, 1:1000 dilution, incubated for 45 min at room temperature). We also labeled kinesin-2 using a monoclonal primary antibody (Mouse Anti KIF3A, ab24626, abcam, directly conjugated with Alexa-568) diluted to 1:500 and incubated at 4°C overnight.

Samples were imaged using an inverted light microscope (Nikon Eclipse Ti-E, Nikon, Japan) equipped with a 100× objective (Apo TIRF 100× OIL DIC N2). The sample was illuminated either with a diascopic brightfield lamp, or with fluorescence excitation lasers (488 nm or 561 nm, respectively). Images were captured with an EMCCD camera (Andor DU-897, Oxford Instruments, UK). Dynein samples were imaged at 12 mW laser power (488 nm) and an exposure time of 500 ms. Kinesin-2 samples were imaged at 10 mW laser power (561 nm) and an exposure time of 500 ms.

An overview of the workflow, including experimental procedures, measured parameters, and the corresponding control experiments is given in Figure S4. The results of the phagosome isolation and immunofluorescence experiments and replication experiments are given in Figure S12.

Determining the unitary step size in the photobleaching experiments. The photobleaching curves were fitted with a piecewise constant function using a step finding algorithm. The resulting step sizes correspond to the intensity of one or multiple fluorophores. Examples of photobleaching curves are shown in Figures S5 and S6. In Figure S5 A photobleaching curves measured in the epifluorescence mode of the microscope on samples with labelled phagosomes are shown. In the phagosome samples, photobleaching curves of small fluorescent spots on the coverslip were evaluated. In larger objects, e.g., complete phagosomes, where the total intensity is 100× to 1000× higher than the unitary step size it is difficult to resolve individual steps, since the variance of the signal (scaling with the square root of the number of fluorophores) became much larger than the intensity of a single fluorophore. The analyzed fluorescent spots, possibly originating from individual antibodies bound unspecifically to the coverslip, had much lower integrated intensities than the phagosomes, making it easier to resolve individual steps. On average, these fluorescent spots had 13 unitary steps. From the unitary step size calibrated with these fluorescent spots, we extrapolated the number of fluorophores on the phagosomes. In Figure S5 B photobleaching curves of samples with individual antibodies measured in the TIRF mode of the microscope are shown. To avoid overfitting, only downward steps > 70 arb. u. were considered for the estimation of the unitary step size.

From the distribution of the step sizes the intensity of a single fluorophore was determined by fitting a Gaussian mixture model (3, 4):

$$\mathrm{GMM}(k, \mu_i, \sigma_i) = \sum_{i=1}^{k} P_i \mathcal{N}_i(\mu_i, \sigma_i),$$

with k normally distributed components $\mathcal{N}_i$ with mean value μ_i, variance σ_i, and weight P_i. The optimal number of components k was chosen to minimize the Bayesian information criterion of the

fit (3, 5). The step size distributions and fitted Gaussian mixture models for epifluorescence and TIRF mode are shown in Figure S5 C and D. The unitary step size, corresponding to the intensity of a single fluorophore, was calculated from the fitted Gaussian mixture model:

$$I_{\mathrm{unitary}} = \sum_{i=1}^{k} \frac{1}{i} P_i \mu_i.$$

For each step size distribution, the Gaussian mixture model was fitted 1000× with random starting points, until the mean of the unitary step size converged. The uncertainty of the unitary step size was calculated from the standard deviation of the unitary step size distribution. We measured a unitary step size of (160 ± 30) arb. units in epifluorescence mode and a unitary step size of (235 ± 50) arb. units in TIRF mode.

Determining the degree of labeling (DOL) of the secondary antibodies. To determine the number of fluorophores coupled to a single secondary antibody, we diluted the secondary antibodies 1:10,000 in 60 µl of MAB, flowed them into a sample chamber and let them attach to the coverslip overnight at 4°C (Figure S5 E). We imaged them in the TIRF mode, measured the intensity of each spot on the coverslip, and corrected the measurements for background. The number of fluorophores per secondary antibody was then calculated as:

$$N_{\mathrm{sAB}} = I_{\mathrm{sAB}} / I_{\mathrm{unitary}}^{\mathrm{TIRF}}$$

On average, we found that 11 ± 1 fluorophores were bound to a single secondary antibody (Figure S5 F). To verify our method, we compared this result to the spectroscopically measured degree of labeling. Using an UV-Vis spectrophotometer (Genesys 10S UV-Vis Spectrophotometer, Thermo Fisher Scientific, Waltham, MA), we measured the absorbance of the antibody solution at 280 nm A_{280} to determine the protein concentration and the absorbance at 495 nm A_{495} to measure the concentration of fluorophores (Table S3). The degree of labeling is then:

$$\mathrm{DOL} = \frac{\epsilon_{\mathrm{IgG}} A_{495}}{(A_{280} - A_{495} \times \mathrm{CF})\epsilon_{\mathrm{DyLight-488}} l_{\mathrm{d}}},$$

with ϵ_{IgG} and $\epsilon_{\mathrm{DyLight-488}}$ the extinction coefficients of IgG at 280 nm and the DyLight-488 fluorophore at 495 nm, the sample path length l_{d}, and a correction factor CF accounting for the absorption of the fluorophore at 280 nm. We measured a degree of labeling of 10 ± 1 fluorophore per single secondary antibody, aligning well with the degree of labeling measured by stepwise photobleaching, thus verifying our photobleaching approach. The degree of labeling that we measured by photobleaching and spectroscopy is technically possible, as there are up to 90 lysines on a single antibody, to which an amine-reactive dye such as DyLight-488 could couple (6, 7). This makes an even larger degree of labeling in principle possible, and likely also explains the variation of the number of fluorophores per secondary antibody that we observed.

Immunofluorescence of the microtubule cytoskeleton. After the cells were fixed with PFA, they were washed 3x and then permeabilized with PBS containing 0.1% Triton-X for 10 min. After washing the cells 3x, they were incubated for 2h at RT with the primary antibody (alpha-Tubulin (DM1A) Mouse mAB, Cell Signaling Technology, Cambridge, UK), washed 3x with PBS-T, incubated for 45 min with the secondary antibody (Invitrogen Goat Anti-Mouse IgG, DyLight 488 conjugated, Thermo fisher Scientific Inc., Waltham, MA), and washed 3x in PBS-T. Finally, they were washed 1x with PBS and transferred to ultrapure water. The coverslips were then mounted on microscopy slides using Fluoromount G (Thermo Fisher Scientific Inc., Waltham, MA).

Modeling of elastic forces due to deformations of the cell cortex. To quantify the potential contributions of cell cortex deformations to the transport forces, we modeled the deformation to estimate the magnitude and scaling behavior of these elastic forces.

In scanning electron micrographs of cells which internalized the magnetic beads, we observed that the beads slightly deformed the outer cortex of the cells, depending on their distance to the nucleus (Figure S7 A). For our model of the deformation of the cell cortex, we assumed that the cell has a wedge-like shape, and the beads deform the cell cortex of the cell if their diameter is larger than the height of the cell at their respective position (Figure S7 B). Heuristically, we assumed that this deformation has the shape of a cosine which smoothly transitions to the undeformed cell at its edges and has a radius of curvature that is equal to the bead radius at its maximum. A sketch of the geometry is shown in Figure S7 C. This model of the deformation shown in Figure S7 B resembles the deformations observed in the scanning electron micrograph.
For a bead with radius $R = D/2$ which is at position x_B along the x axis, the deformation of the cell cortex along the x' axis can be written as:

$$f(x') = h\left[\frac{1}{2}\cos\left(\frac{1}{a}(x' - x'_\mathrm{B})\right) + \frac{1}{2}\right],$$

where h is the height of the deformation perpendicular to the undeformed cortex, and πa the radius of the deformed area of the cell cortex. The deformation height h depends on the position of the bead x_B:

$$\begin{aligned} h &= R - h' \\ &= R - \cos(\theta)\, h'' \\ &= R - y_\mathrm{B}\cos(\theta) + R\cos(\theta) \\ &= R - x_\mathrm{B}\tan(\theta)\cos(\theta) + R\cos(\theta) \\ &= R(1+\cos(\theta)) - x_\mathrm{B}\sin(\theta). \end{aligned}$$

To ensure a smooth transition of the deformation into the undeformed cell, the interval of the deformation ranges from $x'_0 = x'_\mathrm{B} - \pi a$ to $x'_1 = x'_\mathrm{B} + \pi a$. To determine the width of the deformation a, we use the additional condition that the radius of curvature of the deformation at $x' = x'_\mathrm{B}$ must be equal to the bead radius R. The radius of curvature of a function $f(x')$ is defined as

$$r(x') = \left|\frac{(1 + f'(x')^2)^{\frac{3}{2}}}{f''(x')}\right|,$$

where $f'(x')$ and $f''(x')$ are the first and second derivatives of $f(x')$. At the position of the bead the radius of curvature of the deformation therefore is

$$r(x' = x'_\mathrm{B}) = \frac{2a^2}{h} = R.$$

With the deformation height h, this yields

$$a = \left(\frac{1}{2}R[R(1+\cos(\theta)) - x_\mathrm{B}\sin(\theta)]\right)^{\frac{1}{2}}.$$

We assumed that the deformation of the cell cortex is rotationally symmetric around x'_B. Therefore, to calculate the area of the deformed cell cortex in the interval $[x'_0, x'_1]$, we used Pappus' centroid theorem. The arc length L of the function $f(x')$ in the interval from x'_B to $x'_\mathrm{B} + \pi a$ cannot be calculated analytically and was therefore determined numerically. The distance d of its geometric centroid to the axis of rotation is $d = \frac{1}{2}\pi a$ due to the function's symmetry. In total, the area of the deformed actin cortex is

$$A_\mathrm{deformation} = 2\pi d L$$

The corresponding area of the undeformed cell cortex in the interval $[x'_0, x'_1]$ is:

$$A_{\mathrm{undeformedCell}} = \pi^3 a^2$$

The total change in the area of the cell cortex due to the deformation caused by the bead is therefore:

$$\Delta A = A_{\mathrm{deformation}} - A_{\mathrm{undeformedCell}}.$$

With the known tension of the cell cortex of J774A.1 cells σ = 140 ± 20 pN μm^{-1}, which includes tensions of the actin cortex as well as the plasma membrane (8), we can calculate the energy required for a deformation of ΔA of the cell cortex:

$$W_{\mathrm{deformation}} = \sigma \Delta A.$$

The force which pushes the bead along the x axis is the spatial derivative of this energy:

$$F_{\mathrm{Actin}} = -\frac{\partial}{\partial x_{\mathrm{B}}} W_{\mathrm{deformation}}.$$

This derivative was also calculated numerically. The resulting forces by the actin cortex are shown in Figure S7 D. Depending on the size of the bead and its position along the cell, the forces are on the order of a few 10 pN to a few 100 pN. The forces increase with increasing diameter of the beads, however, they only scale linearly with D, and not with D^3 as measured in our experiments (Figure S7 E and F).

Modeling of the stall forces. We developed a theoretical model to explain the scaling behavior of the observed transport forces, taking the size-dependent displacement of microtubules by the phagosomes into account. This size-dependent displacement and bending was directly observed in confocal images of the microtubule cytoskeleton (Figures 4, S8, S16). The model assumes that the total number of microtubules that are displaced by a phagosome is proportional to the organelle's cross-sectional area:

$$N_{\mathrm{MT}} = \rho_{\mathrm{MT}}\, \pi \left(\frac{D}{2}\right)^2.$$

The cytoplasmic microtubule area density ρ_{MT} is the density of microtubules running through a cross-section of the cell orthogonal to its long axis. The model assumes that microtubules which are displaced by a phagosome bend around it, depending on their distance r to the phagosome center. They touch the phagosomes over an interaction angle $\theta_{\mathrm{int}}(r)$ (Figure S8 A). Microtubules with $r = 0$ are strongly displaced and bent by the phagosomes, so that they touch the phagosome over an angle $\theta_{\mathrm{int}}(r = 0) = \theta_0$. With increasing r, the microtubules are displaced and bent less by the phagosomes. Ultimately, microtubules with $r > \frac{D}{2}$ are not displaced or bent by the phagosomes and do not interact with their surface. This displacement and bending of microtubules was directly visible in the confocal images of the microtubules (Figure S8 B). We found experimentally that θ_0 was on average 108° ± 7° and independent of D. Therefore, we treated it as a constant in our model (Figure S16).
Overall, the interaction length $l_{\mathrm{int}}(r)$ on which a microtubule that would cross the phagosome at a given radius r will interact with it, is

$$l_{\mathrm{int}}(r) = \frac{D}{2} \theta_{\mathrm{int}},$$

with the interaction angle θ_{int}. As discussed above, the interaction angle decreases with increasing radius r:

$$\theta_{int} = \theta_0 \left(1 - \frac{2r}{D}\right)$$

Furthermore, only the projection of the force of a motor at a given angle θ along the interaction length of a microtubule contributes to the phagosomal stall force. We assume that motor force is directed along the microtubules, so that a motor at $\theta = \pm 90°$ will direct all its force perpendicular to the force applied by the magnetic tweezers and therefore does not contribute to the stall force. Similarly, we expect that for a motor at $\theta = 0°$, all its force will contribute to the phagosomal stall force (Figure S8 A). Therefore, we weighted the interaction length with $\cos(\theta)$ to obtain an effective interaction length of motors that contribute to the stall force:

$$l_{\mathrm{int,eff}} = \frac{D}{2} \int_{-\frac{\theta_0}{2}(1-\frac{2r}{D})}^{\frac{\theta_0}{2}(1-\frac{2r}{D})} \cos(\theta)\, \mathrm{d}\theta = D \sin\left(\frac{\theta_0}{2}\left(1 - \frac{2r}{D}\right)\right).$$

Dyneins with a maximal distance to a microtubule of one motor length d_{M} may interact with this microtubule (Figure S8 A), leading to an effective area of interaction $A_{\mathrm{int,eff}}$ of about $2\, l_{\mathrm{int,eff}}\, d_{\mathrm{M}}$ on the phagosome. Summing up all the areas of interaction for all the displaced microtubules by integrating over the cross-sectional area of the phagosome $A_{\emptyset}$ yields the effective number of all possible dynein-microtubule interactions that contribute to the stall force:

$$N_{\mathrm{int,eff}}(D) = \rho_{\mathrm{Dyn}}\, \rho_{\mathrm{MT}} \int_{A_{\emptyset}} A_{\mathrm{int,eff}}\, \mathrm{d}A = \rho_{\mathrm{Dyn}}\, \rho_{\mathrm{MT}}\, 2\, d_{\mathrm{M}}\, D \int_0^{2\pi} \mathrm{d}\varphi \int_0^{D/2} \sin\left(\frac{\theta_0}{2}\left(1 - \frac{2r}{D}\right)\right) r\, \mathrm{d}r$$

The integral over r yields

$$\frac{D^2}{2\theta_0^2}\left(\theta_0 - 2\sin\left(\frac{\theta_0}{2}\right)\right) \approx 0.04\, D^2,$$

with $\theta_0 = 108°$ (Figure S16). The integral over φ simply yields 2π. Therefore, the effective number of possible dynein-microtubule interactions is:

$$N_{\mathrm{int,eff}}(D) \approx 0.5\, \rho_{\mathrm{Dyn}}\, \rho_{\mathrm{MT}}\, d_{\mathrm{M}}\, D^3$$

Together with the force of a single dynein F_{SD} and an offset c_{m} we modeled the measured stall forces:

$$F_{\mathrm{stall}}(D) = F_{\mathrm{SD}}\, N_{\mathrm{int,eff}}(D) + c_{\mathrm{m}} = f D^3 + c_{\mathrm{m}},$$

with the force density $f = 0.5\, F_{\mathrm{SD}}\, \rho_{\mathrm{Dyn}}\, \rho_{\mathrm{MT}}\, d_{\mathrm{M}}$. This model fits the experimentally determined stall forces very well and provides a potential explanation for how the dynein numbers increasing with D^2 generate stall forces increasing with D^3.

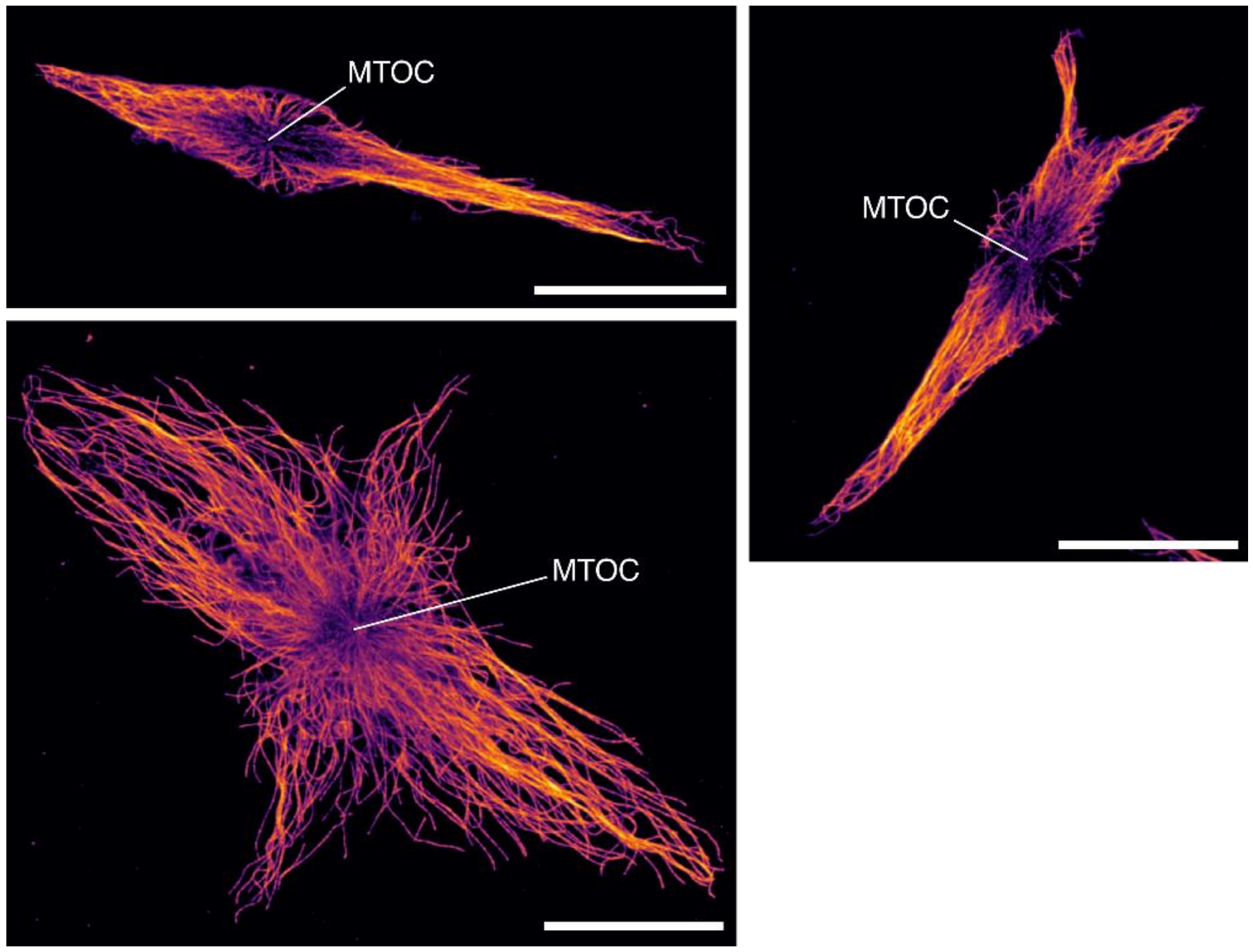

Figure S1: Confocal images of the microtubule skeleton of the J774A.1 cells. The images show that the microtubules originated from a common point, the microtubule organizing center (MTOC). In the cell's lamellipodia, the microtubules were oriented fairly parallel. Beaudet et al. (5) demonstrated that in J774A.1 macrophages, the microtubules are primarily oriented with their plus-ends out. In our transport measurements, the magnetic forces were directed along the long axis of the cells, and therefore acted parallel to the microtubules and opposite to the direction of motion of dynein. Scale bar: 20 µm.

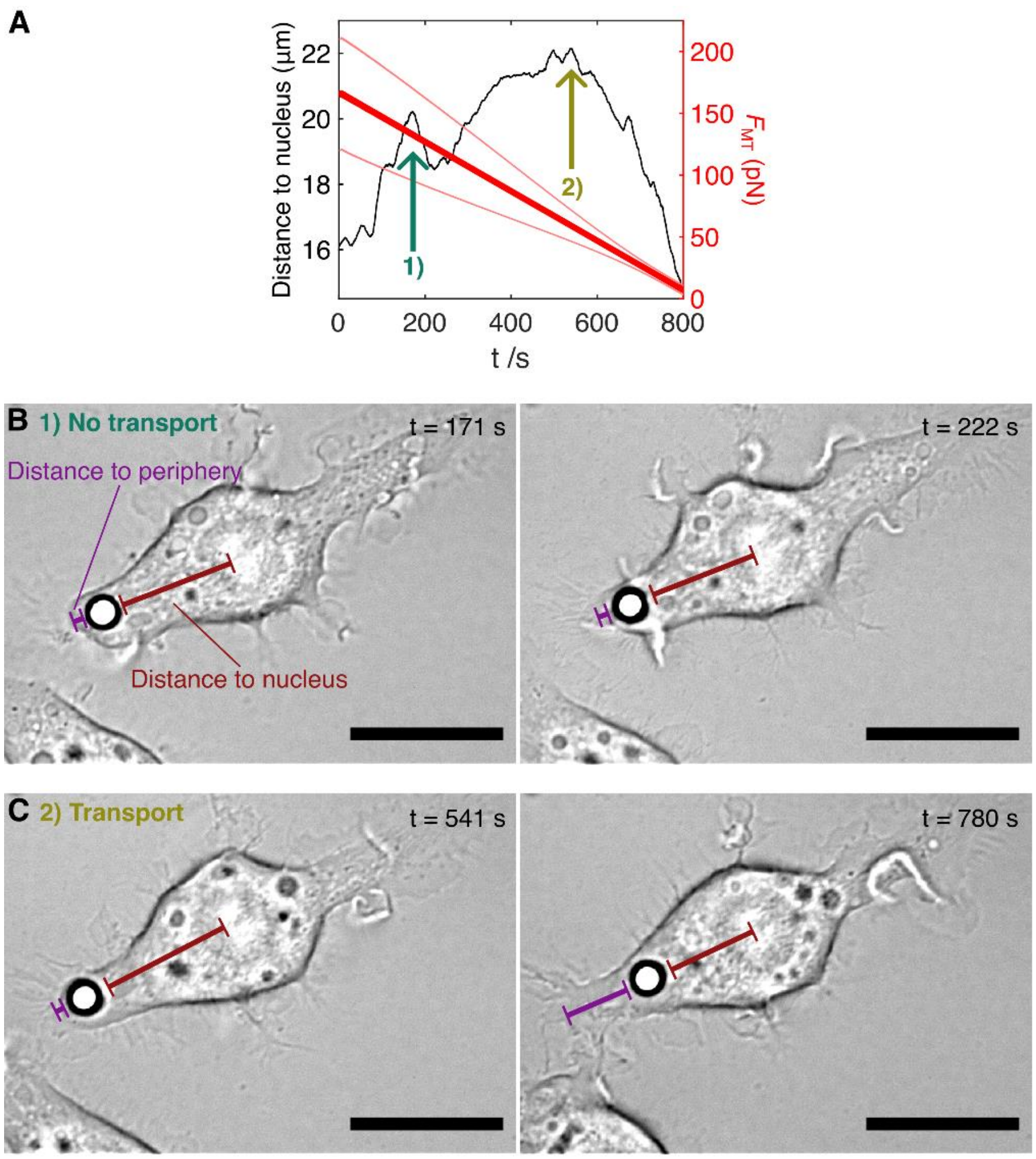

Figure S2: Example of the process to identify retrograde transport events. Here, a measurement of the retrograde transport force for a phagosome with a diameter of is shown. **A:** There are two possible transport events, marked 1) and 2) in the phagosome trajectory. In both cases, the phagosome moves persistently towards the nucleus for more than 1 µm. **B:** However, in the first case, the retrograde movement is caused by remodeling of the cell cortex and retraction of the cell's lamellipodium. During this movement, the phagosome's distance to the nucleus decreased, but its distance to the cell periphery did not change. Therefore, movements like this were not considered for the evaluation of the transport forces. **C:** In the second case, the distance to the nucleus decreased, and the distance to the periphery increased. Therefore, this movement was likely not related to dynamic changes of the cell cortex and was included in the evaluation of the transport forces. Scale bar: 20 µm.

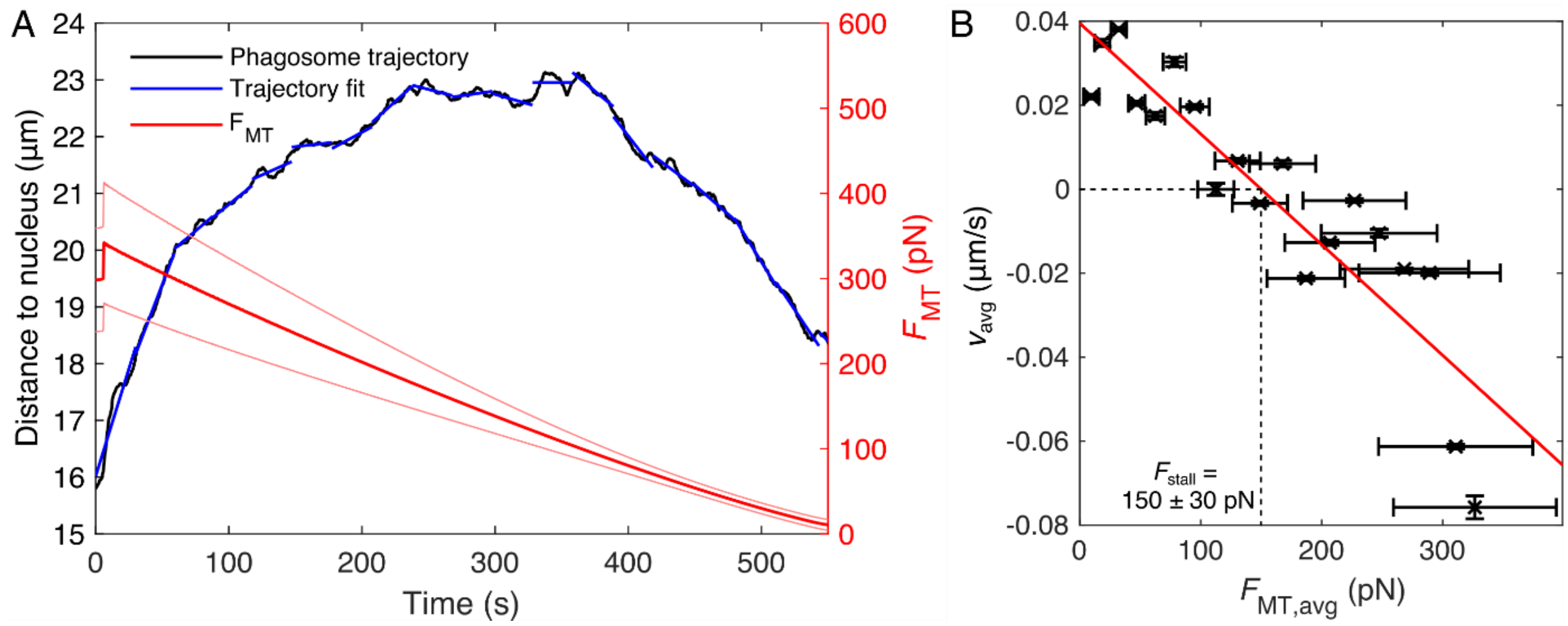

Figure S3: Analysis of the cytoplasmic drag. Stokes' law of friction was fitted to $v_{\mathrm{avg}}(F_{\mathrm{MT,avg}})$. Here, the evaluation procedure for an example measurement with a 5 µm phagosome is shown. **A:** v_{avg} was estimated by fitting a piecewise linear function to the bead trajectories in intervals of 30 s. $F_{\mathrm{MT,avg}}$ was calculated as the arithmetic mean of the magnetic force on the particles in the corresponding intervals. **B**: The data obtained in this way were then fitted with SI equation 1. For this measurement, we observed an effective cytoplasmic viscosity of (80 ± 10) Pa s and a stall force of (150 ± 30) pN.

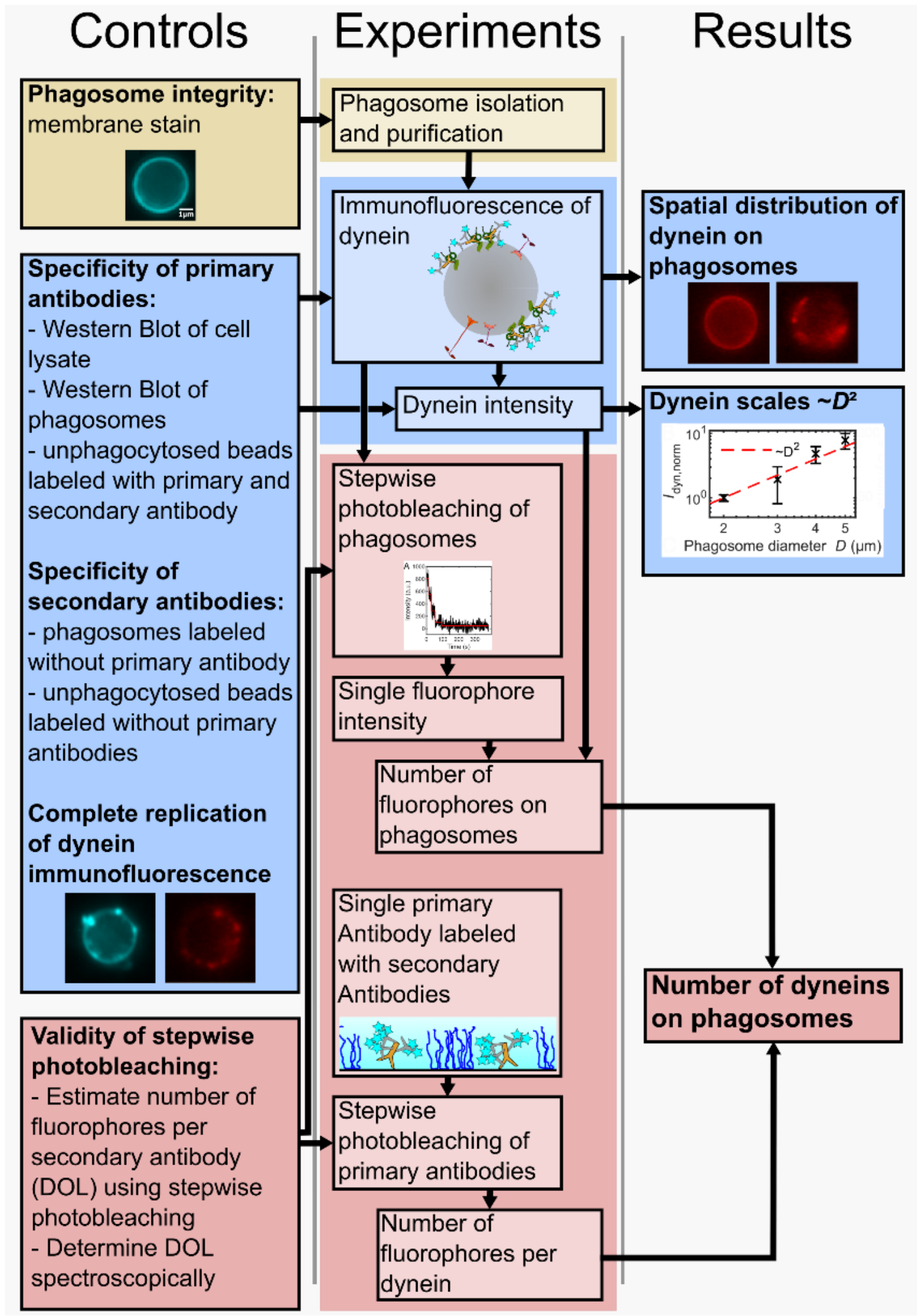

Figure S4: Overview of experiments and corresponding controls. To visualize and quantify dynein on phagosomes we followed a three-step procedure: First, phagosomes were isolated from the cells (color-coded in yellow), then dynein on the isolated phagosomes was labeled via immunofluorescence (color-coded in blue), and lastly dynein was quantified using stepwise photobleaching (color-coded in red). For each step, we performed extensive control experiments.

Briefly, we isolated the phagosomes by homogenizing cells with internalized beads in a homogenization buffer containing EGTA, protease inhibitors, ATP, and DTT. To ensure that the isolated phagosomes were intact, we stained the phagosomal membrane using the fluorescent membrane dye FM4-64.

In the next step, we immunofluorescently labeled dynein on the isolated phagosomes. Briefly, we let the isolated phagosomes sediment on silanized coverslips, which were then blocked using Pluronic F-127. We incubated them with the primary antibodies overnight at 4°C. After thoroughly washing them 2x with buffer containing 0.1% Tween-20 and 1x with buffer only, the phagosomes were incubated with the secondary antibodies for 45 minutes at room temperature. Again, they were washed 2x with buffer containing 0.1% Tween-20 and 1x with buffer only. In the images of the phagosomes, we observed that dynein was not homogeneously distributed over their surface, indicating that dyneins cluster into teams. Furthermore, we measured the total intensity of the dynein signal, which increased with the phagosome surface ($\sim D^2$), indicating that the dynein density on the phagosomes does not depend on phagosome size. To ensure that these conclusions drawn from the immunofluorescence assay are valid we performed extensive controls. We tested the

specificity of the monoclonal primary antibody with Western Blots of total cell lysate and the isolated phagosomes. Furthermore, we labeled unphagocytosed beads with primary and secondary antibodies to ensure that no unspecific binding to the beads occurred. In addition, we tested the specificity of the secondary antibody by labeling both phagosomes and unphagocytosed beads with the secondary antibody only. Lastly, we replicated the immunofluorescence assay of dynein with new phagosomes and different types of primary and secondary antibodies.

To quantify the number of dyneins on the phagosomes from their fluorescence intensity, we performed stepwise photobleaching on the phagosomes. This technique allows us to estimate the intensity of single fluorophores in the sample. To ensure a good resolution of the individual steps, we focused on “dim” trajectories that only had about 5-15 steps. Knowing the intensity of a single fluorophore, we extrapolated the number of fluorophores attached to the phagosomes. To determine the number of dyneins, we additionally needed to calibrate the number of fluorophores per dynein. We therefore incubated silanized coverslips in a solution of diluted primary antibodies, so that individual antibodies could attach to the coverslip. After blocking the coverslip with Pluronic F-127, we labeled the primary antibodies with the secondary antibodies as described above. Performing stepwise photobleaching on these samples yielded the number of fluorophores attached to a primary-secondary antibody complex. Since we used a monoclonal antibody with a known epitope on the dynein intermediate chain, we calculated the number of fluorophores attached to a single dynein. Combining the number of fluorophores on the phagosomes with the number of fluorophores per single dynein allowed us to determine the number of dyneins per phagosome. We validated this technique by estimating the degree of labeling (DOL) of the secondary antibody using stepwise photobleaching. We then compared this value to the DOL measured with a well-established spectroscopical method (Table S3). Both values agreed within a 10% margin, underlining the validity of the stepwise photobleaching.

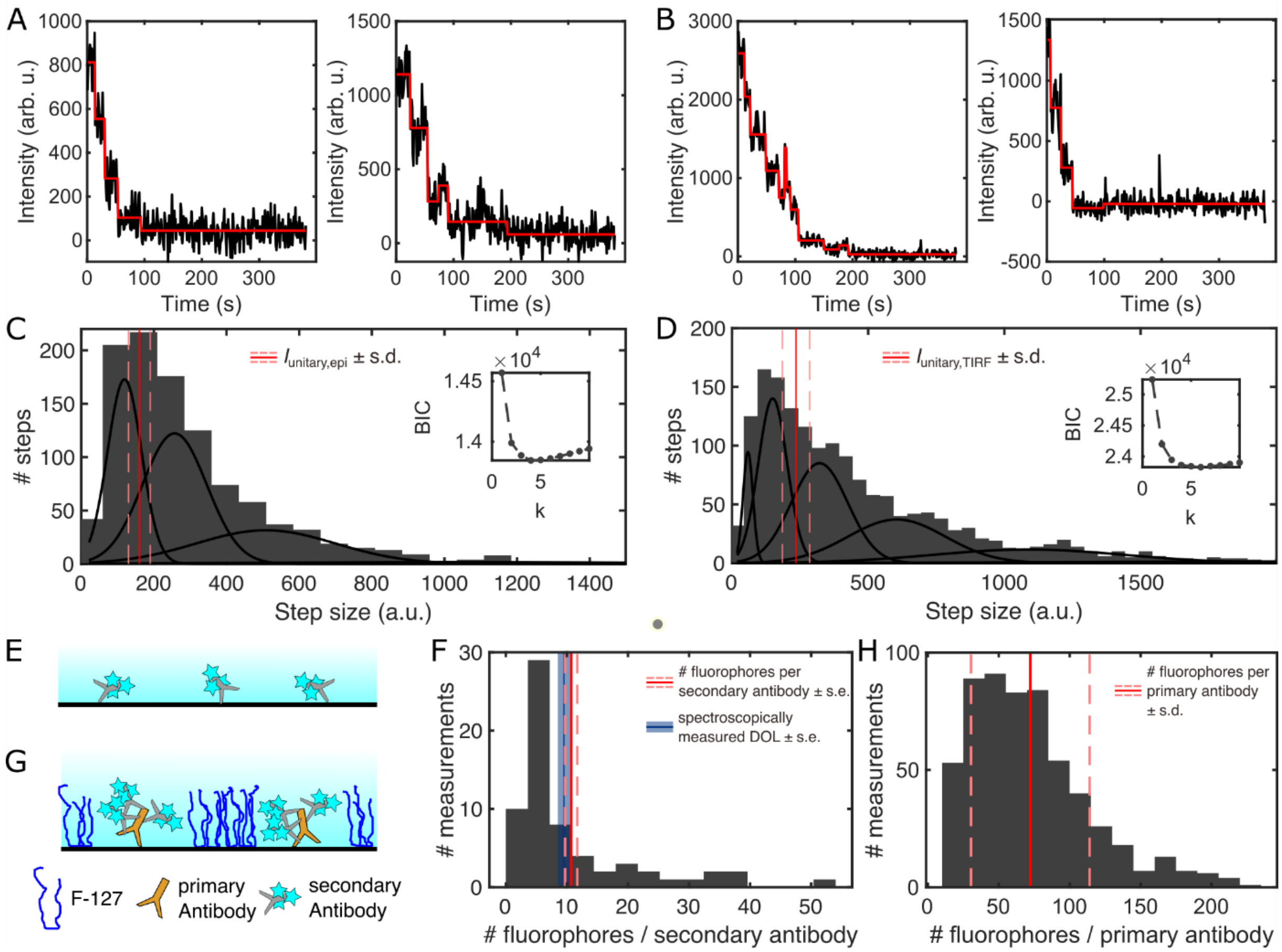

Figure S5: Quantification of dynein numbers by stepwise photobleaching. A: Examples of photobleaching curves measured in the epifluorescence mode of the microscope. Discrete steps in the signal correspond to one or multiple fluorophores bleaching. The signal was fitted with a piecewise constant function using an algorithm based on a two-sided t-test. More details about the acquisition of the photobleaching curves are provided in Figure S6. **B:** Examples of photobleaching curves measured in the TIRF mode of the microscope, fitted with a piecewise constant function. **C:** The distribution of step sizes was fitted with a Gaussian mixture model to estimate the unitary step size in the epifluorescence mode, which corresponds to the intensity of a single fluorophore. The number of components for the Gaussian mixture was determined by evaluating the Bayesian information criterion (small inset). We found a unitary step size of (160 ± 30) arb. u. in the epifluorescence mode. **D:** In TIRF mode, we analogously estimated a unitary step size of (235 ± 50) arb. units. **E:** To estimate the numbers of fluorophores bound to a single secondary antibody, we let diluted secondary antibodies attach to the coverslip and evaluated their intensity in TIRF mode. **F:** Using the unitary step size in TIRF mode, we found that on average 11 ± 1 fluorophores are bound to a single secondary antibody. This agreed very well with the independently measured spectroscopic degree of labeling (DOL) of 10 ± 1 fluorophores per secondary antibody. The variation of the number of fluorophores per secondary antibody likely originated from the dye's chemical coupling process, binding covalently to lysine residues on the IgG. With roughly up to 90 lysine residues on a single antibody, larger numbers of fluorophores per antibody are in principle possible (6, 7). **G:** Similarly, we determined the number of fluorophores bound to a single primary-secondary antibody complex. Therefore, we let diluted primary antibodies attach to the coverslip, blocked the coverslip with F-127, and labelled the primary antibodies with the secondary antibodies. **H:** We observed that on average 70 ± 40 fluorophore are present in a single primary-secondary

antibody complex. Since each dynein complex carries two dynein intermediate chains, we expect two primary antibodies and therefore about 140 fluorophores per dynein complex.

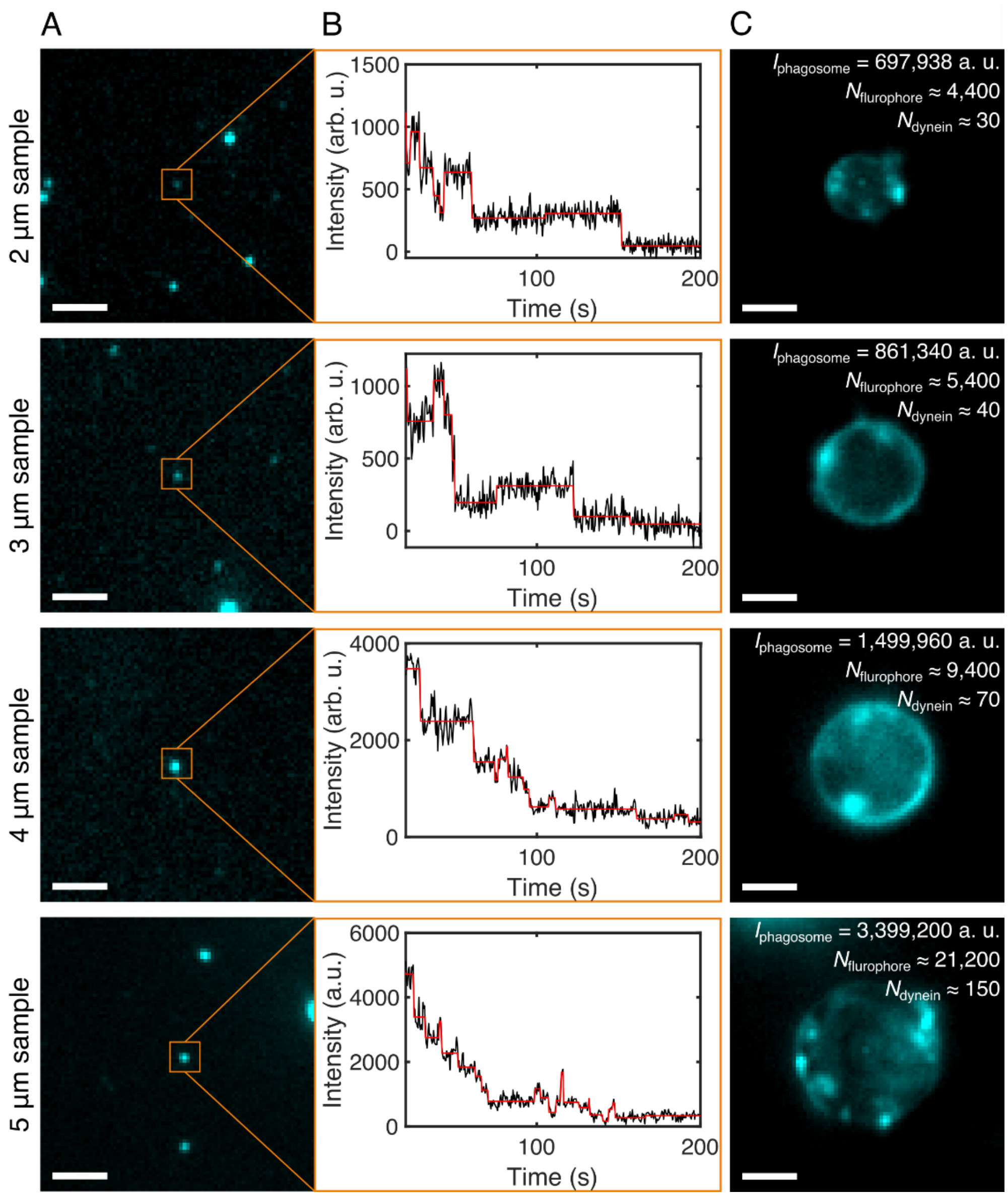

Figure S6: Detailed examples of photobleaching curves. A: In the phagosome samples with immunofluorescently labeled dynein, photobleaching curves of small fluorescent spots on the coverslips were evaluated. These spots might have originated from unspecific binding of the antibody to the coverslips or binding to remnants of cell debris in the phagosome samples, which adhered to the coverslips in small amounts. On average, the intensity of these fluorescent spots was 13× the unitary step size. In larger objects, like complete phagosomes, resolving individual steps was challenging due to the signal variance exceeding the single fluorophore intensity. **B:** The resulting photobleaching curves were fitted with a piecewise constant function. To avoid overfitting, only downward steps > 70 arb. u. were considered. Each step corresponds to the intensity decrease for one or multiple bleaching fluorophores. The photobleaching curves from all samples were evaluated together to determine the intensity of a single fluorophore $I_{\mathrm{unitary}}^{\mathrm{epi}}$ = 160 ± 30 arb. u. **C:** Examples of phagosomes with immunofluorescently labeled dynein. The fluorescent spots shown in **A** and the phagosomes shown in **C** are from the same sample, respectively. Using $I_{\mathrm{unitary}}^{\mathrm{epi}}$ we calculated the number of fluorophores $N_{\mathrm{Fluorophore}}$ per phagosome. With the number of

fluorophores per dynein (Figure S5), we then determined the number of dyneins N_{dynein} per phagosome. Scale bars: 2 μm.

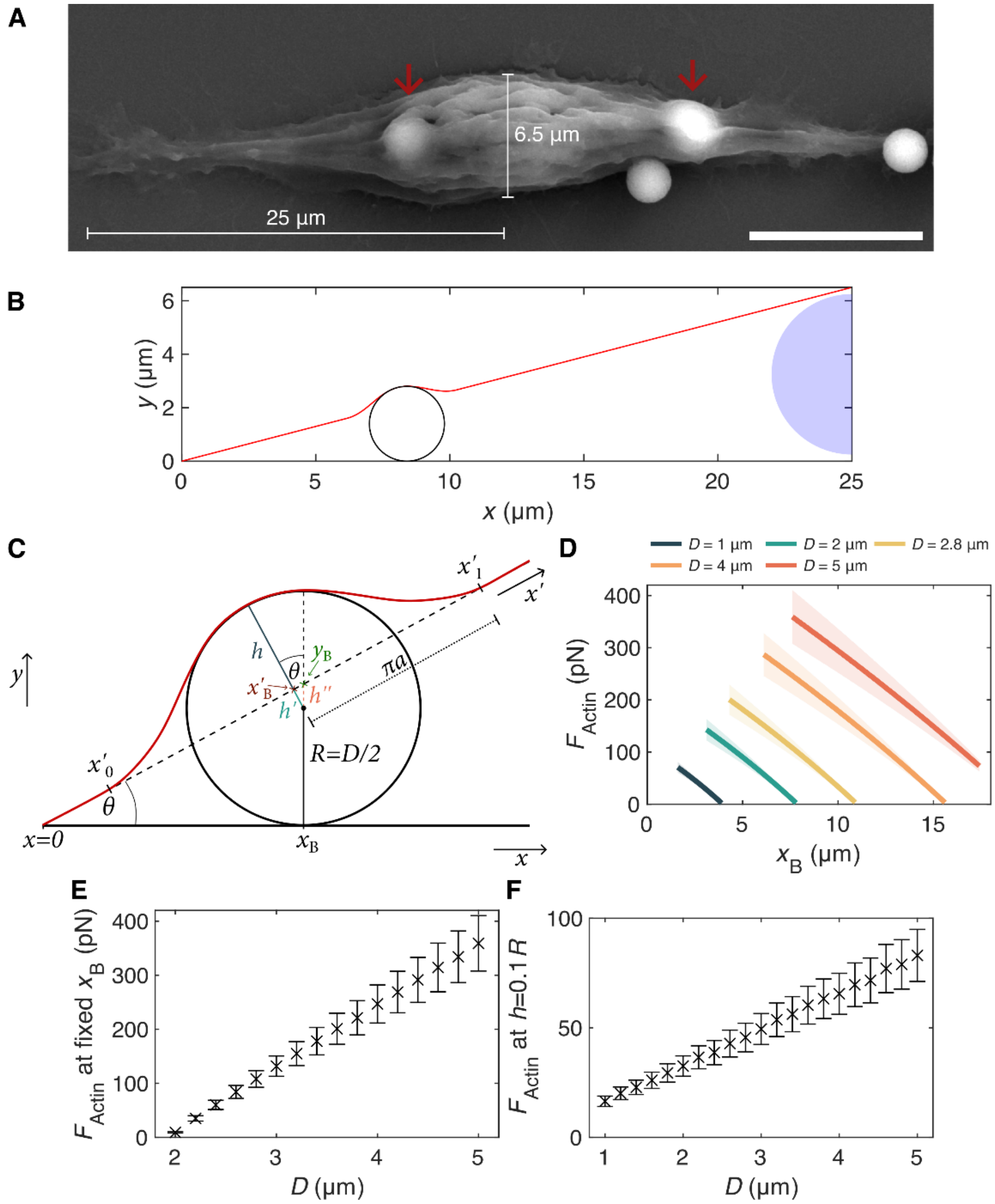

Figure S7: Model of the elastic forces by the cell cortex. A: Scanning electron micrograph of a J774A.1 cell which internalized two 2.8 µm beads (highlighted with red arrows). Depending on their position, the beads slightly deformed the cell cortex. **B:** To model this deformation, we assumed that the cell has a wedge-like shape, and the deformation can be described heuristically by a cosine, which smoothly transitions into the undeformed cell at its edges and has a radius of curvature that is equal to the bead radius at its center. **C:** Sketch of the geometry of the deformation. **D:** Resulting forces of the elastic models. Forces are on the order of 10-400 pN and depend on the phagosomes' size and position along the cell. **E:** At a fixed bead position x_B of 7.4 µm, the elastic forces due to the actin cortex increase linearly with phagosome size. **F:** Likewise, the elastic forces increase linearly with phagosome size for a fixed deformation of $2h/D$ = 0.1.

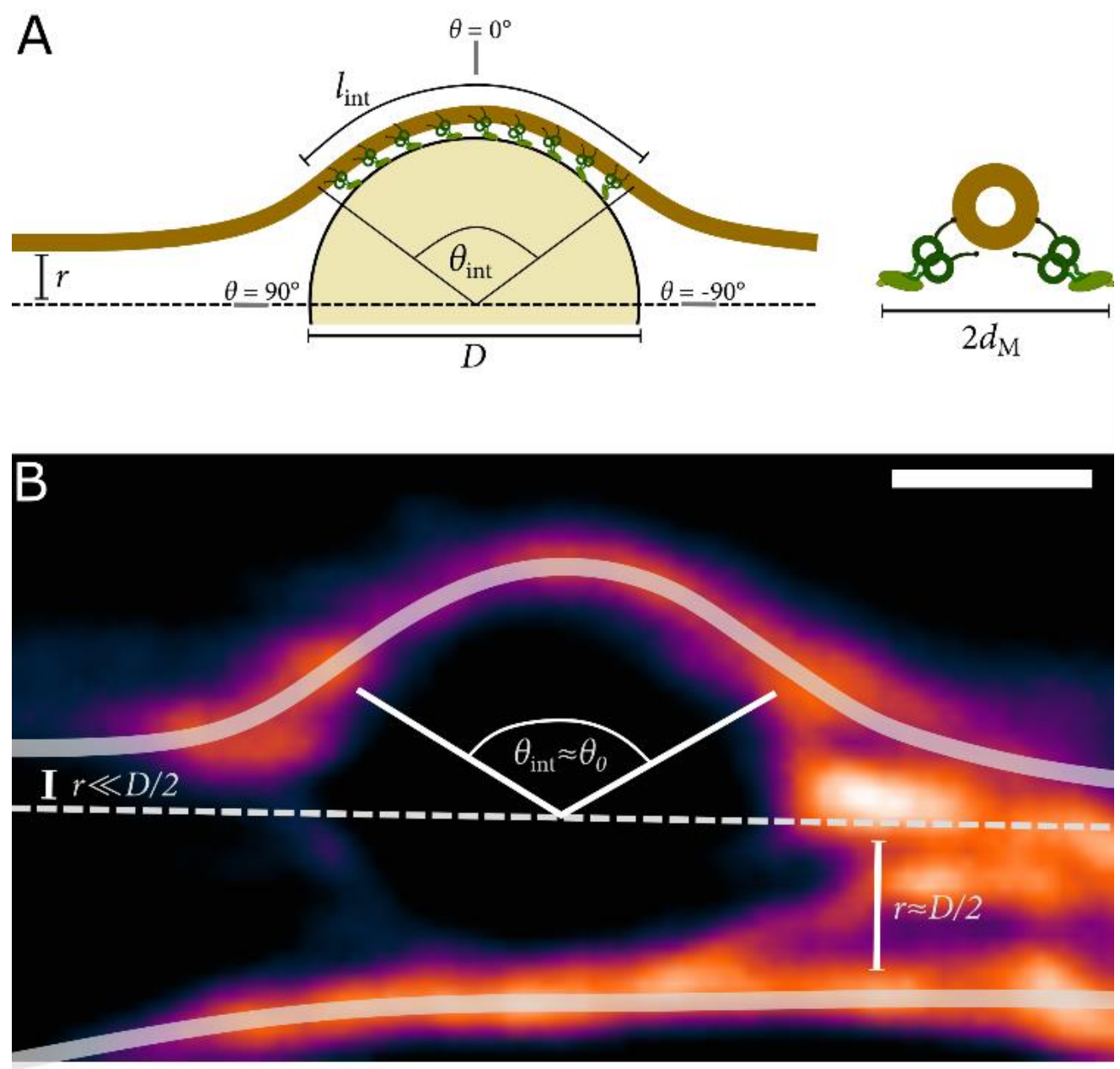

Figure S8: Geometry of microtubule bending. A: Microtubules bend around organelles depending on their distance r to the phagosome center. Microtubules that would run close to the phagosome center $r \approx 0$ are strongly displaced and bent by the phagosome. These microtubules touch the phagosomes over an angle of $\theta_{\mathrm{int}}(r \approx 0) \approx \theta_0$. Motors that are closer than d_{M} to this microtubule may interact with it, leading to an interaction area A_{int} on the phagosome in which motors can interact with this microtubule. **B:** The displacement and bending of microtubules by phagosomes was directly visible in the confocal microtubule images. Here, a microtubule with $r \ll \frac{D}{2}$ is strongly displaced and bent around the phagosome, another microtubule with $r \approx \frac{D}{2}$ barely is affected. The confocal image shows a phagosome in a fixed cell which was not subjected to an external magnetic force. Scale bar: 1 µm.

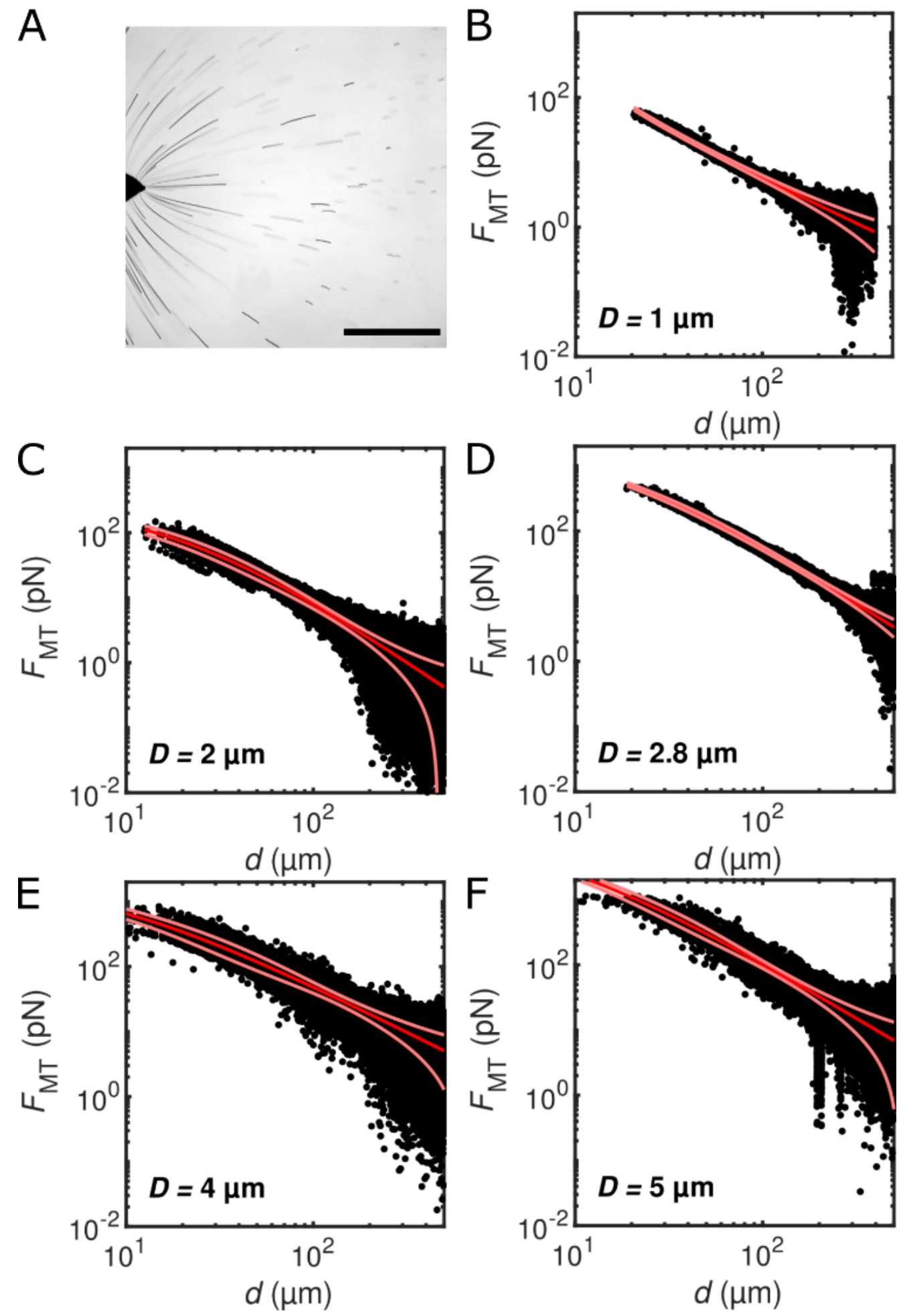

Figure S9: Calibration of magnetic tweezers. We analyzed the motion of the magnetic particles towards the tip of the magnetic tweezers in a viscous glycerol-water solution. **A:** Minimal intensity projection of a calibration measurement. Scale bar: 200 µm. The magnetic beads move along the magnetic field gradient with a velocity v that depends on the tip-bead distance d. Using Stokes' law of friction, we evaluated the force-distance relationship $F_{\mathrm{MT}}(d)$ for each bead type (**B-F**). The resulting calibration curves were heuristically fitted with a power law $F_{\mathrm{MT}}(d) = F_0 / \left(\frac{d-r_0}{d_0}\right)^c$, shown in red. The uncertainty of the calibration curves (shown in light red) was determined from the scattering of the data points, containing tracking errors and variations in size and magnetic moment of individual beads, and the uncertainty of the viscosity of the calibration. Sample size $N = 77{,}277$ (1 µm phagosomes), 501,545 (2 µm), 401,077 (2.8 µm), 389,425 (4 µm), 414,788 (5 µm).

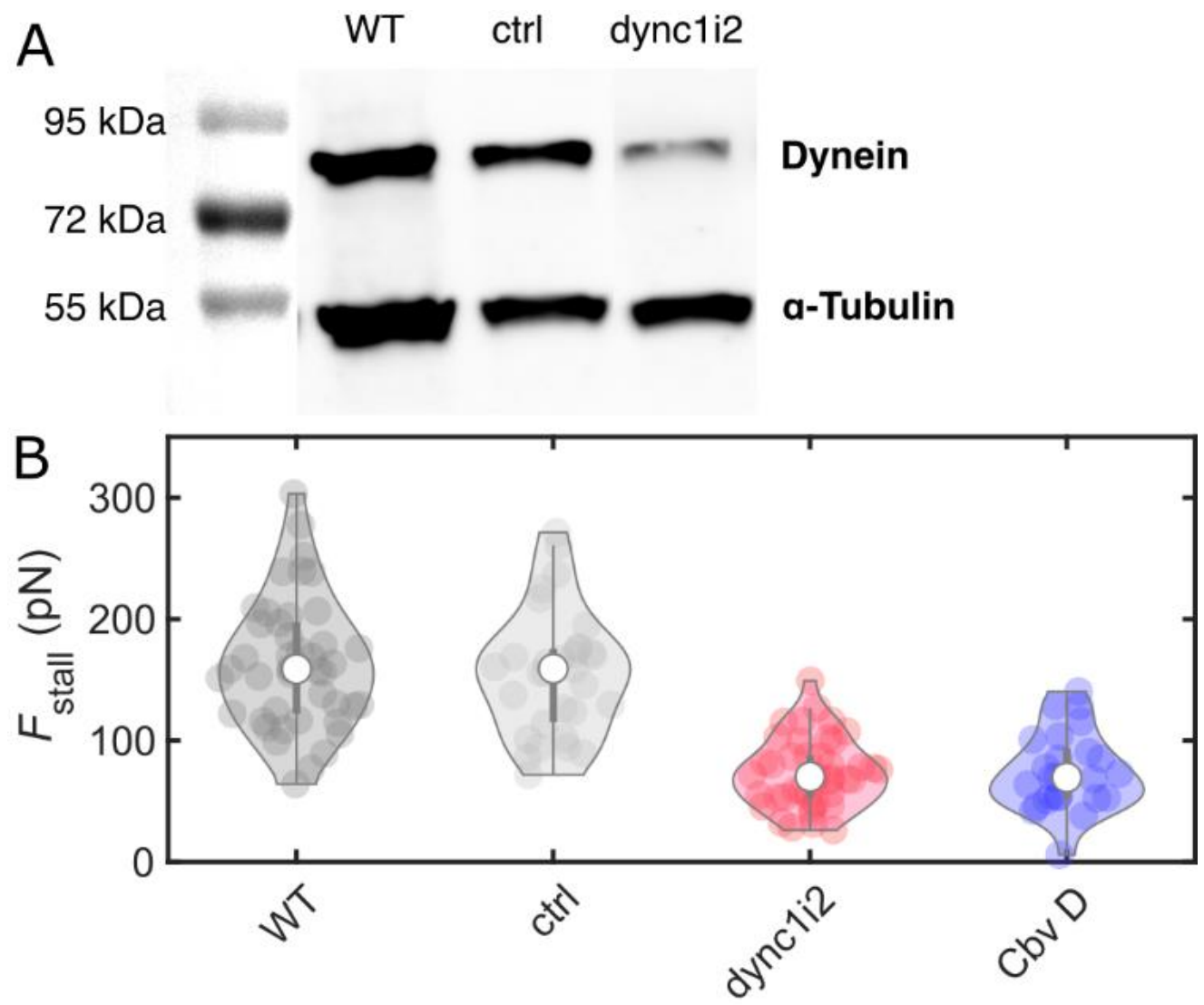

Figure S10: Dynein knockdown and inhibition experiments. To investigate the role of dynein in the transport of phagosomes, we treated cells with siRNA targeted to dync1i2 and the small-molecule dynein inhibitor ciliobrevin D, respectively. **A:** The Western blot indicates that the expression level of the dynein intermediate chain dync1i2 was reduced to (50 ± 10) % compared to the negative control treatment and the wild-type cells. **B:** Transport forces of phagosomes with a diameter of 5 μm for cells treated with siRNA targeted to dync1i2 and 50 μM ciliobrevin D (Cbv D). The forces in the case of both treatments were reduced to about 50% of the value of the negative control and the wild-type cells. This is consistent with a reduction of the expression level of dynein to about 50% in the siRNA-treated cells and the reduction of its ATPase activity to about 50% in the ciliobrevin-treated cells, respectively. Our results indicate that in this case mainly dynein is driving the centripetal transport of the organelles. Sample size $N = 44$ (WT), 31 (ctrl), 42 (dync1i2), 25 (CbvD).

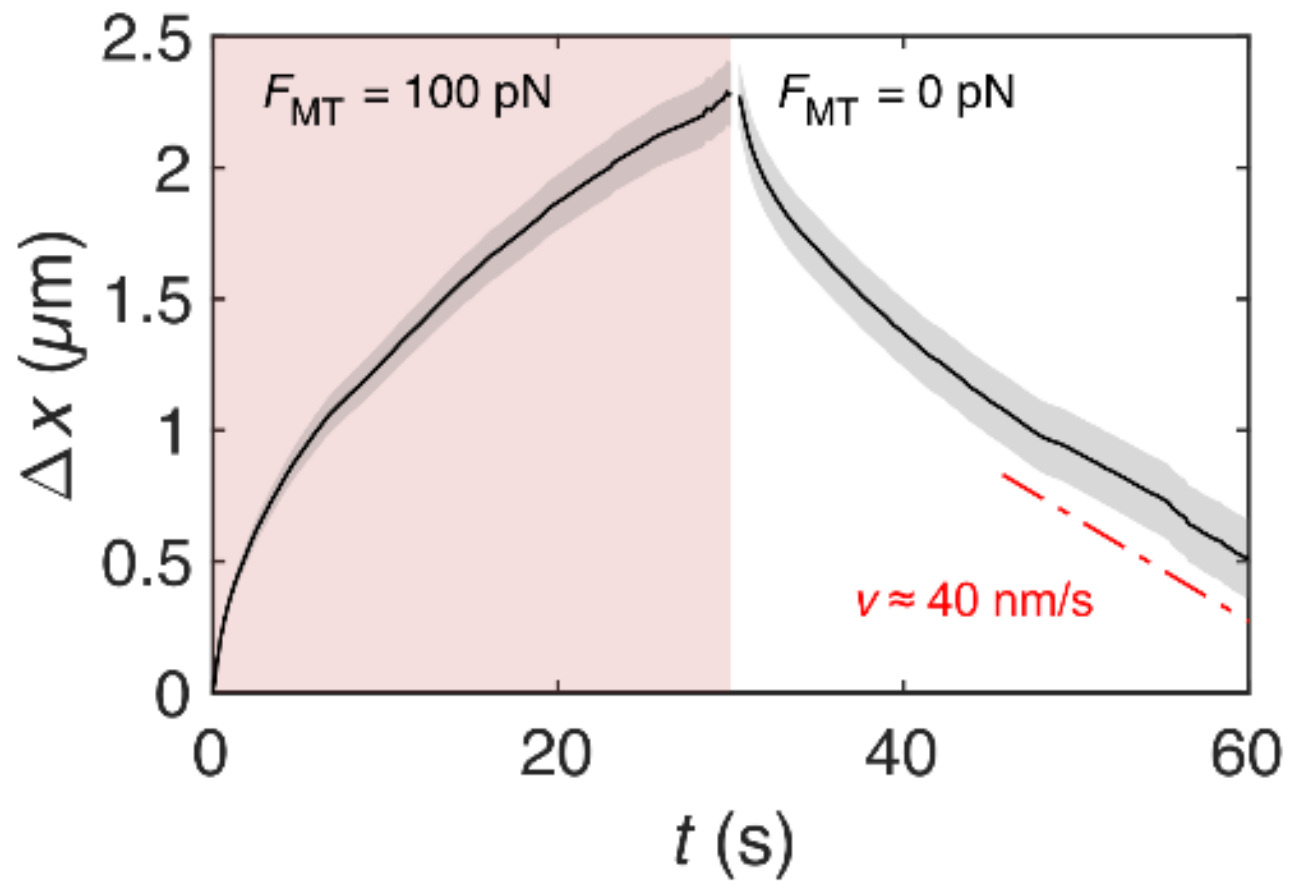

Figure S11: Measurement of cell rheology. To measure the rheological properties of the cells, we applied a high alternating magnetic force of 100 pN to the 2.8 μm phagosomes. The force was periodically switched on and off with a period of 60 s. Here, the mean displacement Δx of the phagosomes from their initial position over one switching cycle is shown. The magnetic force F_{MT} was turned on at *t* = 0 s and kept constant at 100 pN (red shaded area) until it was switched off again at *t* = 30 s. When the magnetic force was on, the particle was displaced from its original position. This displacement followed a power law, as described previously (9, 10). Once the magnetic force was turned off, the phagosome started to relax back towards its original position. However, the phagosomes did not fully reach their initial position, which indicates that there was an inelastic deformation of the cell cortex due to its viscoelastic properties. Interestingly, the creep function seemed to transition from a power law to a linear function 15-20 s after the magnetic force was turned off. Then, the phagosome moved with a constant velocity of approximately 40 nm s^{-1}, which is on the same order of magnitude as the free transport velocity observed during retrograde transport (Figure 2 F). To further analyze this behavior, we fitted a power law of the form $\Delta x(t) = x_0 t^{\varepsilon}$. In the context of power-law rheology, the scaling exponent ε of this power law shows whether the environment of the phagosome behaves like an elastic solid ($\varepsilon = 0$) or viscous fluid ($\varepsilon = 1$) (9, 10). Since cells are viscoelastic and have both elastic and viscous components, $0 < \varepsilon < 1$ should be expected (9). Consistently, we observe for the creep phase (*t* = 0-30 s) an exponent $\varepsilon = 0.521 \pm 0.004$. Interestingly however, the recovery phase can be divided into two parts: In the first half of the recovery phase (*t* = 30-45 s), the viscoelastic exponent is very similar to the creep phase, with $\varepsilon = 0.578 \pm 0.002$. However, in the second half (*t* = 45-60 s), the recovery has transitioned to a purely viscous-like motion, with a viscoelastic exponent of $\varepsilon = 1.02 \pm 0.02$. This result indicates that elastic forces no longer play a role, and the phagosome moves with a constant velocity, consistent with free transport by dyneins.

In total, we analyzed 97 phagosome trajectories in the creep phase and 86 phagosome trajectories in the recovery phase, originating from 14 individual cells. Shaded areas of uncertainty represent the standard error of the mean.

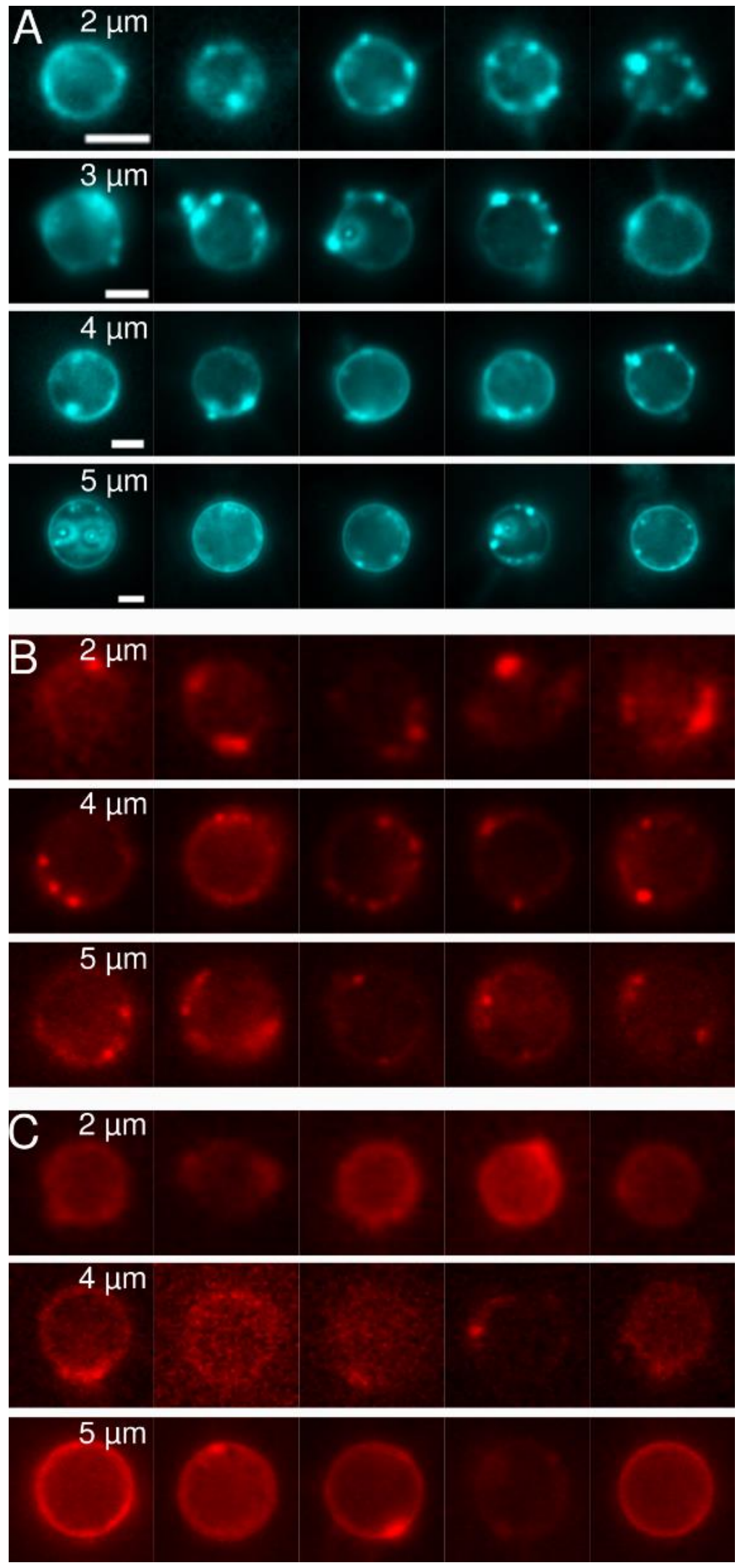

Figure S12: Replication of dynein immunofluorescence. To verify our immunofluorescence assay, we replicated the labeling of dynein with new phagosomes and different types of antibodies. The new phagosomes were isolated from the cells following the same procedure as before. However, instead of homogenizing the cells with a syringe we now used a dounce homogenizer. The original antibodies were Invitrogen Dynein Monoclonal Antibody (74.1), Thermo Fisher Scientific, Catalogue Number MA1-070 (primary) and Invitrogen Goat Anti-Mouse IgG, DyLight 488 conjugated, Thermo Fisher Scientific (secondary). For the replication, we used Dynein HC Antibody, Santa Cruz, Catalogue Number sc-9115 (primary), and Goat Anti-Rabbit IgG – Atto 488, Sigma Aldrich, Catalogue Number 18772 (secondary). The results obtained from the original and new immunofluorescence assay of dynein were remarkably similar. **A:** Examples of the original dynein labelling assay. Dynein localized predominantly to discrete spots, indicating clustering of dynein into teams. **B:** The same localization pattern was observed in the replicated dynein assay. **C:** We also labeled kinesin-2 on the new phagosomes, which was previously reported to be present on phagosomes (3, 11). Unlike dynein, kinesin was distributed homogeneously over the phagosomes' surface, indicating that the localization pattern observed for dynein was indeed not an artifact.

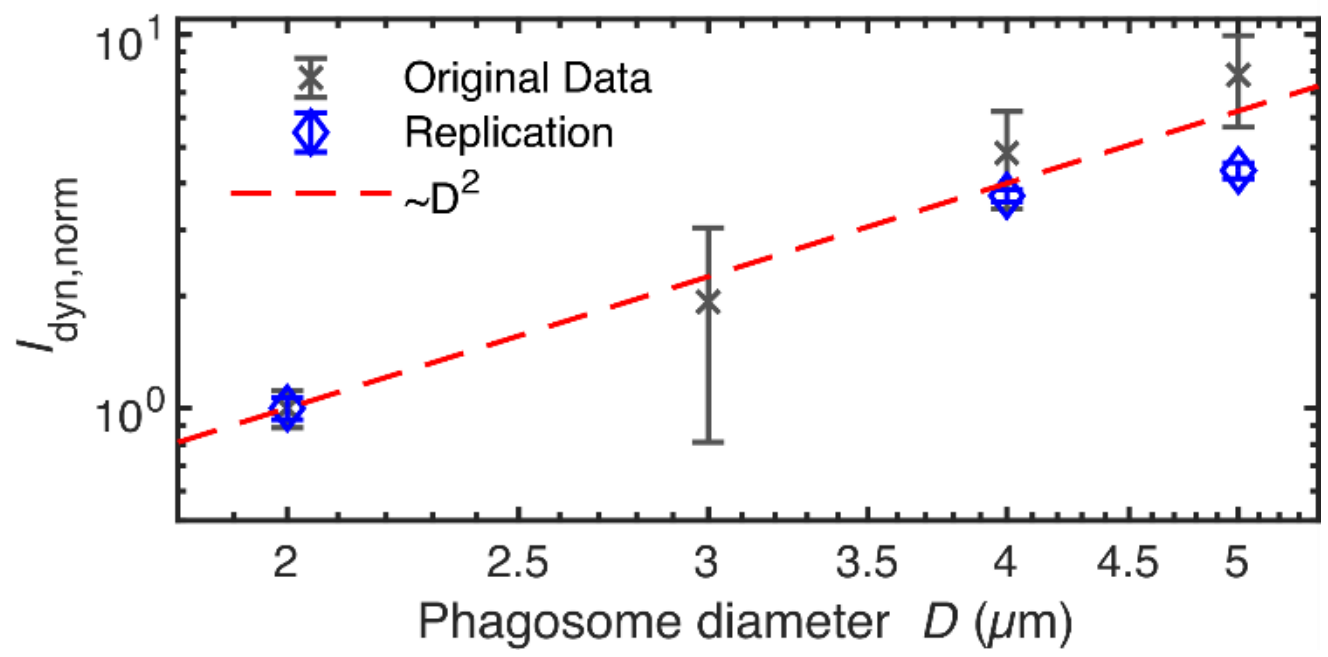

Figure S13: Comparison of original and replicated dynein intensities. We additionally determined the intensity of dynein for phagosomes of different size in the replicated immunofluorescence assay. In the replicated assay, we used a different batch of isolated phagosomes, different primary and secondary antibodies, different fluorophores, and a different microscopy setup. Like in the original data, the dynein intensity did not scale with $\sim D^3$, but rather with $\sim D^2$. Therefore, both the original and replicated immunofluorescence assays are consistent with a constant dynein surface density on the phagosomes, independent of phagosome size. The amount of dynein present on the phagosomes alone is thus not sufficient to explain the scaling $\sim D^3$ of the transport forces.

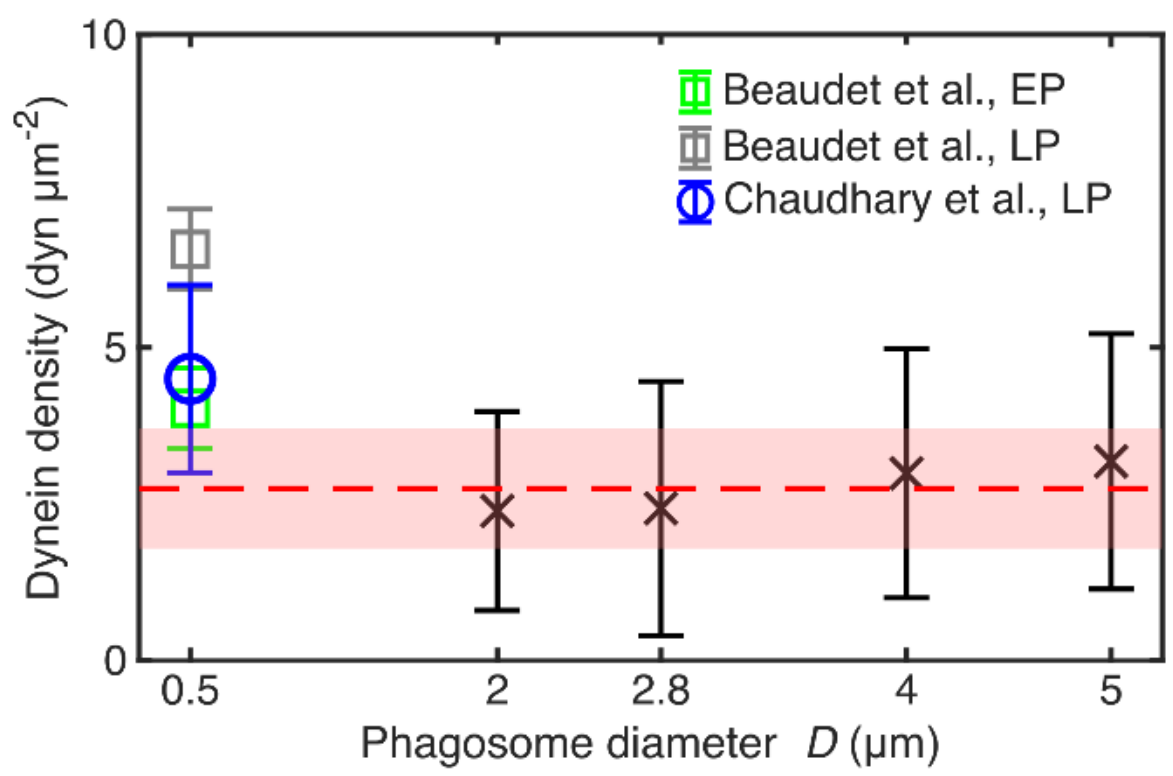

Figure S14: Comparison of the dynein surface density of phagosomes of different size with dynein densities published previously. Beaudet et al. (5) measured the dynein surface density on 0.5 µm phagosomes 30 min (early phagosomes, EP) and 90 min (late phagosomes, LP) after the particles were added to the cells. Chaudhary et al. (3) measured the dynein surface density of 0.5 µm phagosomes 90 min after the particles were added to the cells. The phagosomes used in this work were isolated 60 min after the beads were added to the cells. The dynein surface density of 3 ± 1 dyneins µm^{-2} we measured in this work were within the margins of uncertainty consistent with the dynein surface densities published earlier. The differences between our values and those of Beaudet et al. and Chaudhary et al. might be caused by the different duration of phagosomal maturation, slight differences in the treatment of the cells during phagosome isolation (e. g., homogenization with syringe instead of dounce homogenizer), and different procedures during purification and immunostaining of the phagosomes (e. g., magnetic separation of isolated phagosomes vs. ultracentrifugation). Overall, the agreement of the dynein surface density measured in this work and those published earlier underlines the validity of our procedures and reinforces our hypothesis that the dynein surface density is independent of phagosome size over a large range of diameters (0.5 µm – 5 µm).

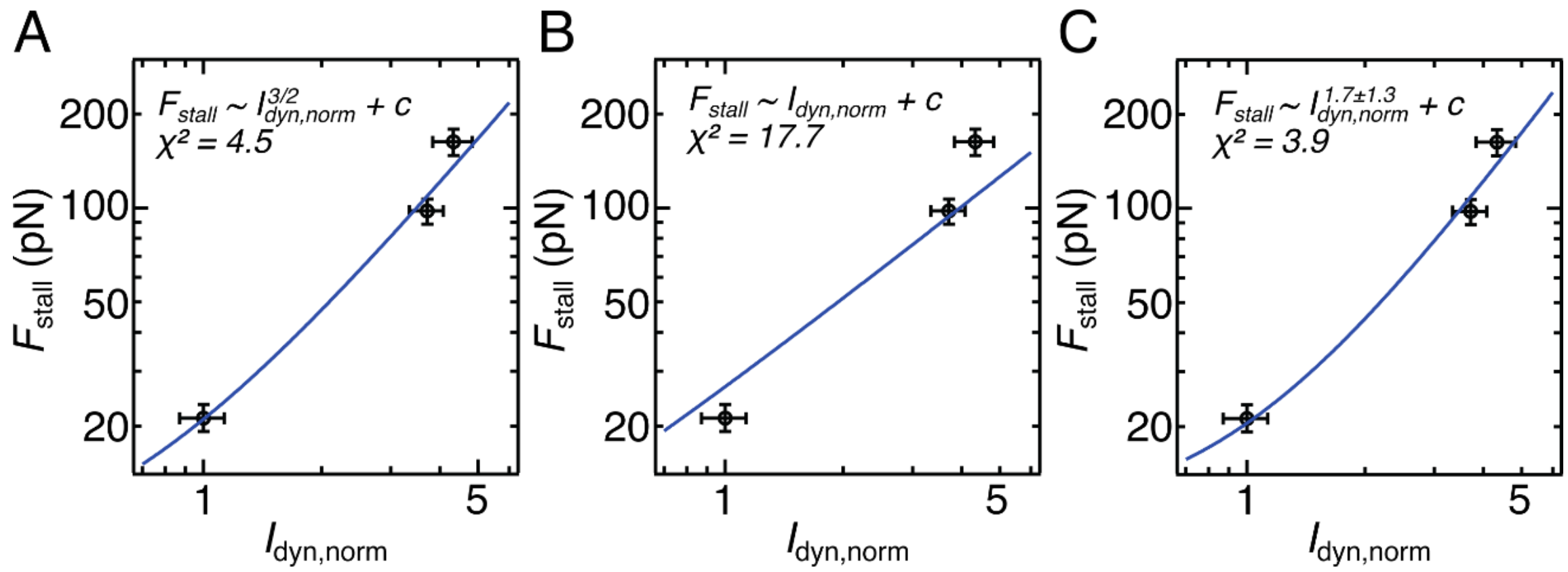

Figure S15: Stall forces as a function of the dynein fluorescence. To assess the force-motor scaling relationship, the stall phagosomal stall force F_{stall} was plotted against the corresponding normalized dynein intensity $I_{\mathrm{dyn,norm}}$. They were fitted with $F_{\mathrm{stall}} \sim I_{\mathrm{dyn,norm}}^{3/2} + c$ **(A)**, $F_{\mathrm{stall}} \sim I_{\mathrm{dyn,norm}} + c$ **(B)**, and a free scaling exponent **(C)**. The constant offset c was included to acknowledge that due to our measurement method, at least one force producing unit was active during stall-force measurements. The χ^2 goodness-of-fit values suggest a better fit for $F_{\mathrm{stall}} \sim I_{\mathrm{dyn,norm}}^{3/2}$, which is consistent with the scaling exponent of 1.7 ± 1.3 for the free fit.

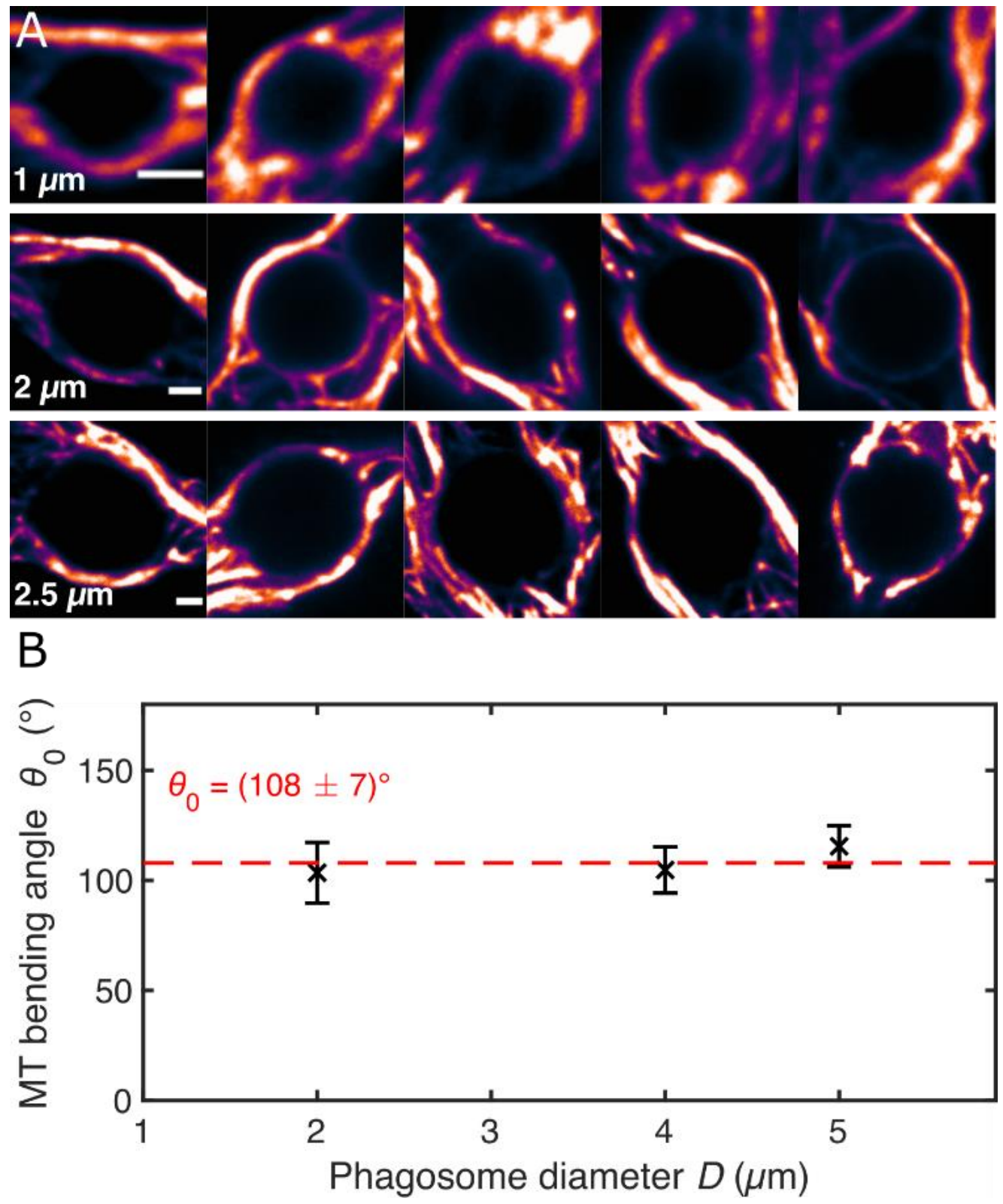

Figure S16: Analysis of microtubule binding angles. A: Examples of microtubules bending around phagosomes of different sizes. Scale bar: 1 μm. The confocal images show phagosomes in fixed cell which were not subjected to an external magnetic force. **B:** To investigate, whether the MT bending angle $\boldsymbol{\theta_0}$ depended on the organelle diameter D, we measured θ_0 using the angle tool of the image processing package Fiji. We always measured the interaction angle of microtubules that would run close to the microtubule center, $r \approx 0$ (compare Figure S8 B). We found that the microtubule bending angle was on average 108° ± 7° and that is was independent of the organelle radius. Therefore, we treated it as constant in our model. Sample size $N = 10$ (2 μm phagosomes), 14 (4 μm), 9 (5 μm). Error bars represent standard deviation.

Table S1: Resulting fit parameters from the cytoplasmic viscosity analysis. For the effective cytoplasmic drag η_{eff} see Table 1. The phagosome trajectories were analyzed using Stokes' law of friction: $v_{\text{avg}}(F_{\text{MT,avg}}) = \frac{1}{3\pi\,\eta_{\text{eff}}\,D}(F_{\text{stall}} - F_{\text{MT,avg}})$. The average free transport velocity corresponds to the phagosomes' average velocity without an external magnetic force: $v_0 = v_{\text{avg}}(F_{\text{MT,avg}} = 0)$.

D (µm)	F_{stall} (pN)	v_0 (nm s^{-1})
1	11 ± 10	23 ± 13
2	25 ± 10	20 ± 8
2.8	32 ± 15	24 ± 9
4	110 ± 40	25 ± 7
5	170 ± 40	37 ± 5

Table S2: Results for the fit parameters of the magnetic tweezers calibration. Results are shown for each particle size.

D (μm)	F_0 (pN)	r_0 (μm)	c	a (pN)	b (μm)	k (pN)
1	35	5.16	1.3466	1.35	15.61	0.4
2	180	-19.24	1.9962	24.8	27.22	0.5
2.8	929	-15.01	1.8582	44.1	34.47	1.0
4	399	-7.80	1.4488	154	29.31	3.8
5	1269	-7.33	1.7307	430	23.82	6.3

Table S3: Spectroscopic measurement of the degree of labeling (DOL) of the secondary antibody used to label dynein on isolated phagosomes. Measurements were performed in the storage buffer of the antibody (PBS @ pH 7.2 with 0.02% of sodium azide) and replicated 5 times. We the measured absorption at 280 nm (protein concentration) and 495 nm (dye concentration). Measurements were corrected for the absorption of the blank sample (PBS + 0.02% sodium azide). With the given spectroscopic properties of the DyLight 488 dye and IgG, we estimated that the degree of labeling of this antibody was 10 ± 1 fluorophores per antibody, which is in excellent agreement with the photobleaching measurements (11 ± 1 fluorophores per antibody). These measurements underline the validity of our stepwise photobleaching analysis.

	A_{280}	A_{495}	Degree of labeling
Individual replicates	0.838	0.254	9.52
	0.869	0.254	9.16
	0.860	0.289	10.6
	0.911	0.259	8.90
	0.845	0.254	9.43
**Average ± standard deviation**	**0.87 ± 0.03**	**0.26 ± 0.02**	**10 ± 1**

Table S4: Measured dynein intensities I_{dyn} on isolated phagosomes. Here, the dynein intensities measured on isolated phagosomes of the original assay and replication assay are provided in arb. u.

D (µm)	Original assay (95% CI)	Replication assay (95% CI)
2	6.9E+05 (5.5E+05 … 8.4E+05)	1.3E+06 (1.2E+06 … 1.5E+06)
3	1.3E+06 (0.2E+06 … 3.0E+06)	-
4	3.3E+06 (2.0E+06 … 4.8E+06)	5.0E+06 (4.5E+06 … 5.5E+06)
5	5.3E+06 (3.5E+06 … 7.3E+06)	5.8E+06 (5.1E+06 … 6.5E+06)

SI References

1. Keller, S., K. Berghoff, and H. Kress. 2017. Phagosomal transport depends strongly on phagosome size. *Sci. Rep.* 7:17068.
2. Crocker, J.C., and D.G. Grier. 1996. Methods of digital video microscopy for colloidal studies. *J. Colloid Interface Sci.* 179:298–310.
3. Chaudhary, A.R., F. Berger, C.L. Berger, and A.G. Hendricks. 2018. Tau directs intracellular trafficking by regulating the forces exerted by kinesin and dynein teams. *Traffic*. 19:111–121.
4. Chen, Y., N.C. Deffenbaugh, C.T. Anderson, and W.O. Hancock. 2014. Molecular counting by photobleaching in protein complexes with many subunits: best practices and application to the cellulose synthesis complex. *Mol. Biol. Cell*. 25:3630–3642.
5. Beaudet, D., C.L. Berger, and A.G. Hendricks. 2024. The types and numbers of kinesins and dyneins transporting endocytic cargoes modulate their motility and response to tau. *J. Biol. Chem.* 300:107323.
6. Holmes, K.L., and L.M. Lantz. 2001. Protein labeling with fluorescent probes. In: Methods in Cell Biology. . pp. 185–204.
7. Wu, K.-L., C. Yu, C. Lee, C. Zuo, Z.T. Ball, and H. Xiao. 2021. Precision Modification of Native Antibodies. *Bioconjug. Chem.* 32:1947–1959.
8. Lam, J., M. Herant, M. Dembo, and V. Heinrich. 2009. Baseline Mechanical Characterization of J774 Macrophages. *Biophys. J.* 96:248–254.
9. Berghoff, K., W. Gross, M. Eisentraut, and H. Kress. 2021. Using blinking optical tweezers to study cell rheology during initial cell-particle contact. *Biophys. J.* 120:3527–3537.
10. Kollmannsberger, P., and B. Fabry. 2011. Linear and nonlinear rheology of living cells. *Annu. Rev. Mater. Res.* 41.
11. Hendricks, A.G., E.L.F.F. Holzbaur, and Y.E. Goldman. 2012. Force measurements on cargoes in living cells reveal collective dynamics of microtubule motors. *Proc. Natl. Acad. Sci.* 109:18447–18452.
